# Alternative splicing events implicated in carcinogenesis and prognosis of thyroid gland cancer

**DOI:** 10.1038/s41598-021-84403-6

**Published:** 2021-03-01

**Authors:** Zeng-Hong Wu, Yun Tang, Yue Zhou

**Affiliations:** 1 Department of Otorhinolaryngology, Union Hospital, Tongji Medical College, Huazhong University of Science and Technology, Wuhan, Hubei China; 2 Department of Infectious Diseases, Union Hospital, Tongji Medical College, Huazhong University of Science and Technology, Wuhan, 430022 China; 3 Department of Critical Care Medicine, Union Hospital, Tongji Medical College, Huazhong University of Science and Technology, Wuhan, 430022 China

**Keywords:** Cancer genetics, Cancer genomics, Cancer

## Abstract

Alternative splicing (AS), a critical post-transcriptional regulatory mechanism, expands gene expression patterns, thereby leading to increased protein diversity. Indeed, more than 95% of human genes undergo alternative splicing events (ASEs). In this study, we drew an all-around AS profile of thyroid cancer cells based on RNA-seq data. In total, there were 45,150 AS in 10,446 thyroid cancer cell genes derived from 506 patients, suggesting that ASEs is a common process in TC. Moreover, 1819 AS signatures were found to be significantly associated with the overall survival (OS) of TC patients. Kaplan–Meier survival analyses suggested that seven types of ASEs were associated with poor prognosis of TC (*P* < 0.05). Among them, exon skipping (ES) was the most common, with alternate promoter (AP) and alternate terminator (AT) coming second and third, respectively. Our results indicated that acceptor sites (AA) (AUC: 0.937), alternate donor sites (AD) (AUC: 0.965), AT (AUC: 0.964), ES (AUC: 0.999), mutually exclusive exons (ME) (AUC: 0.999), and retained intron (RI) (AUC: 0.837) exhibited an AUC greater than 0.6. In addition, age and risk score (All) were risk factors for TC patients. We also evaluated whether TC-ASEs are regulated by various splicing factors (SFs). We found that the expression of 90 SFs was associated with 469 ASEs and OS of TC patients. Our findings provide an insight into the role of spliceosomes in TC, which may offer novel perspectives in tumor research.

## Introduction

Thyroid cancer (TC) is the most prevalent endocrine tumor, and is a common head and neck malignant tumor. Previous studies have reported that its incidence accounts for 1% of all malignant tumors^1,2^. Significant increase in the incidence of TC in recent decades has led to widespread public concern. Moreover, its distant metastases and lymph node metastases have been associated with high morbidity and mortality^3^. Thyroid cancer can be divided into four pathological types including papillary thyroid cancer (PTC), anaplastic thyroid cancer (ATC), follicular thyroid cancer (FTC), and medullary thyroid cancer (MTC)^4^, with papillary thyroid cancer accounting for about 90% of all TC cases^5^. One study reported that the 35-year or 40-year survival rate of most PTC patients exceeds 80% after effective treatment^6^. However, patients who are not sensitive to radioactive iodine therapy or have cervical lymph node metastasis at the time of diagnosis have a poor prognosis, with the 10-year survival rate being less than 10%^7^. This calls for the identification of prognostic biomarkers which can diagnose TC recurrence and metastasis. Gene regulation dysfunction is a key factor in tumor occurrence and development.

Alternative splicing (AS), a critical post-transcriptional regulatory mechanism, expands gene expression patterns, thereby leading to increased protein diversity. More than 95% of human genes undergo alternative splicing events (ASEs) and encode splice variants in the regular physiological processes^8^. Alternative splicing is widely involved in several biological processes such as cell differentiation, proliferation, and apoptosis. Previous studies have reported that aberrant ASEs modulate cancer metastasis, progression, immunotherapy, and therapeutic resistance, and they may provide opportunities for novel cancer therapeutics^9–13^. In addition, alternative mRNA processing may be a potential target for cancer immunotherapy^12^. Alternative splicing includes seven fundamental splicing patterns^14,15^: Alternate acceptor sites (AA), alternate promoter (AP), alternate donor sites (AD), alternate terminator (AT), mutually exclusive exons (ME), exon skipping (ES), and retained intron (RI). Several studies have reported that aberrant AS is a common event in the development and progression of numerous cancers including gastrointestinal adenocarcinomas and urogenital malignancies^16–19^. Xie et al.^20^ constructed a novel combined prognostic model for ASEs and clinicopathological parameters in esophageal carcinoma. Wang et al.^21^ analyzed ASEs using whole-genome methods and developed a prognostic model for endometrial cancer. Furthermore, Chen et al.^22^ used ASEs to develop a prognostic index which could accurately predict overall survival (OS) in hepatocellular carcinoma. However, despite the existence of several studies on ASEs^23^, the role of AS in thyroid cancer has not been fully elucidated. Therefore, we explored the combination of splicing and clinical parameters, and potential mechanism of the survival-related splicing events in TC. An all-around AS profile of thyroid cancer was drawn after analyzing RNA-seq data, and prognostic models were developed by combining splicing signatures and clinicopathological parameters. Finally, we constructed a splicing network with the overarching goal of providing functional insights into the role of AS in the initiation and development of thyroid cancer.

## Materials and methods

### Source of raw data

We downloaded the target RNA sequence data of TC patients from The Cancer Genome Atlas (TCGA) database (https://www.cancer.gov/tcga), a web-based resource which provides a user-friendly interface for detailed views of alternative mRNA splicing based on the TCGA database and Percent Spliced In (PSI) degrees ranging from 0 to 1 (PSI cutoff used based on FDR (false discovery rate) < 0.05). Thus, PSI was used in quantifying the ASEs retrieved from TCGA^24^. In total, we extracted sequence data of 495 thyroid cancer and 58 adjacent normal tissues, and the clinicopathological data of TC patients. Table 1 shows the characteristics of 506 thyroid cancer patients in the TCGA database. The data was then used to explore changes in ASEs, and its association with carcinogenesis and prognosis of TC. It is worth noting that all the TCs enrolled in this study were adenomas and adenocarcinomas.

**Table 1:** The characteristics of thyroid cancer patients in TCGA.

Variable	Number of samples
**Gender**	
Male/female	370/136
**Age at diagnosis**	
≤ 65/ > 65/NA	435/71
**Stage**	
I/II/III/IV/NA	285/52/112/55/2
**T**	
T1/T2/T3/T4/NA	144/166/170/24/2
**M**	
M0/M1/NA	284/9/213
**N**	
N0/N1/NA	230/226/50

### Survival-associated splicing events and clinical parameters

We only included the clinical data of TC patients with an OS of 90 days or longer. Each clinical parameter was classified into either a high-risk (≥ median number) or low-risk (< median number) group. Cox regression analysis was then used to determine the relationship between AS events and OS as well as the prognostic value of demographic and clinicopathological parameters of TC. The prognosis risk score was calculated as previously described^19^. We performed Kaplan–Meier survival analysis to determine the survival significance of the signatures, while ROC curves were used to assess the predictive value of the prognostic signatures. We then selected the top 20 from each type of splicing and seven combined events.

### Construction of gene network and correlation analysis

We explored whether splicing factors (SFs) regulate ASEs because previous studies have shown that AS is regulated by SF. The SFs used in this study were obtained from the dataset reported by Seiler et al.^25^. Spearman correlation method was used to determine the relationship between all SFs and PSI of ASEs. The association was considered to be significant if the correlation coefficient was |*R*^2^|> 0.6 at P < 0.001. Next, we constructed the connection network between SFs and ASEs using Cytoscape (version 3.7.1). Gene set enrichment analysis (GSEA) creates an arranged list of all genes in accordance with their connection with target gene expression. Thus, we performed GSEA to define the AS-ALL model in the Kyoto Encyclopedia of Genes and Genomes (KEGG). Normalized enrichment scores (NES) and nominal *P*-values were then used to classify the enriched pathways in each phenotype. Statistical significance was set at *P* < 0.05 and false discovery rate (FDR) at q < 0.25.

### Statistical analysis

All statistical analyses were performed using R software V. 3.5.3. The aggregates and intersections between the seven different types of AS were demonstrated graphically using the UpSetR package^26,27^. The hazard ratios (HRs) at 95% confidence intervals (CIs) were used to evaluate relative risk of TC patients based on the seven PSI of ASEs in the different risk groups. Moreover, univariate and multivariate cox regressions were performed in order to identify survival-associated SFs. Finally, differential analysis was conducted using one-way ANOVA, with disease state (Tumor or Normal) being a variable for calculating differential expression in the GEPIA database. *P* < 0.05 was considered to be statistically significant.

### Ethical approval

As the work is a bioinformatics analysis article, ethical approval was not necessary and all the data were retrieved from the free online databases.

## Results

### Overview of ASEs in TCGA-TC

**Figure 1:**
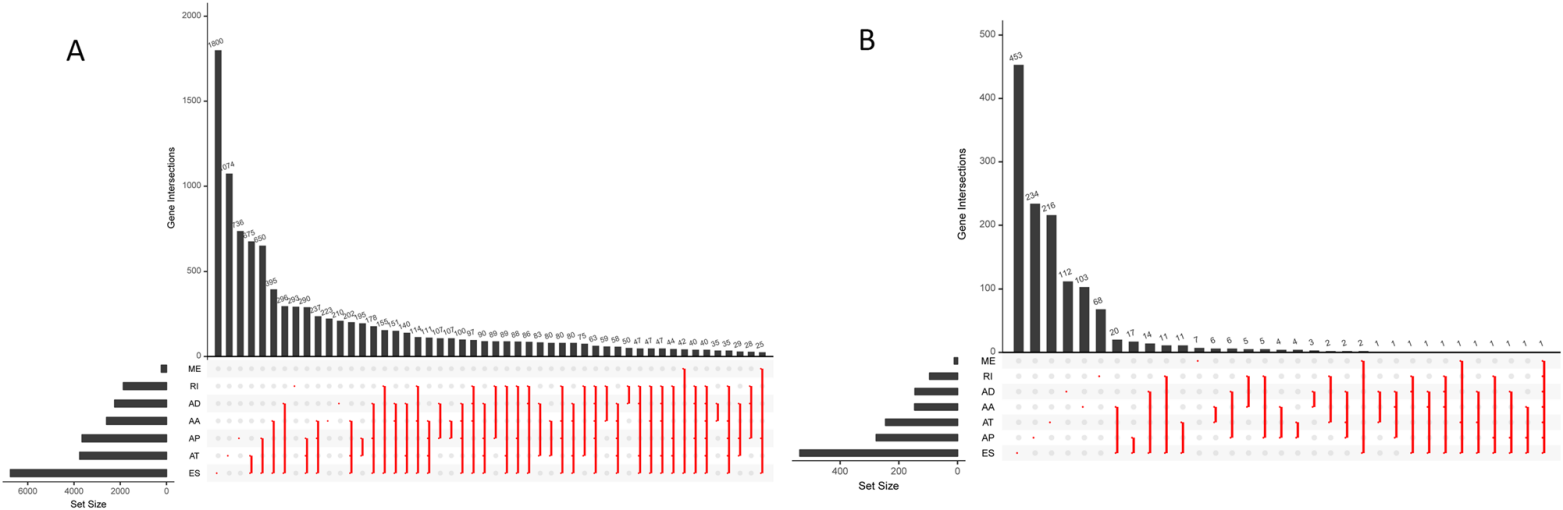
**(A)** Upset plots for the top 50 gene intersections of the seven types of ASEs identified in this study. The dark bar on the right of each drawing represents the number of each type of ASEs. The dark dots in the matrix at the bottom part of each drawing represents the intersections of AS events, while the dark bar on the top represents the number of gene intersections corresponding to the ASEs marked by the red line below. The red line indicates at least two ASEs. **(B)** A subset of overlapping overall survival associated ASEs among the seven types of AS in TC. The y-axis represents the number of genes in each gene. Set size represents the number of genes in each AS.

Each ASE was allocated a unique annotation, which was a combination of the gene name, identification (ID) number, and the AS type in the SpliceSeq database (AS ID). For instance, an annotation such as “*FNTA*-83754-AD” can be broken down as follows; *FNTA* is the gene name, 83,754 is the AS ID, and AD is the splicing pattern. It is worth noting that one gene can undergo different types of AS. The UpSet image was then used to match the genes with corresponding ASEs, which quantitatively assessed different interactive sets. Our results indicated that there were 45,150 ASEs from 10,446 genes and 506 TC patients, and the median AS for every gene was 4.322. Among the ASEs, 4481 genes underwent 8594 AT, 2449 genes underwent 3189 AD, 4793 genes underwent 9126 AP, 2799 genes underwent 3684 AA, 7485 genes underwent 17,536 ES, 2035 genes underwent 2786 RI, 2449 genes underwent 3189 AD, and 217 genes underwent 232 ME. Notably, one gene could have multiple survival associated AS events. Detailed information about the specific AS types of genes was visualized using an Upset plot (Fig. 1A), which can demonstrate quantitative results of multiple interactive sets more effectively than traditional Venn diagram. The Upset plot indicated that ES was the most common of the seven types of ASEs, with AP and AT coming second and third, respectively (Fig. 1B).

### Survival related ASEs in TCGA-TC

**Figure 2:**
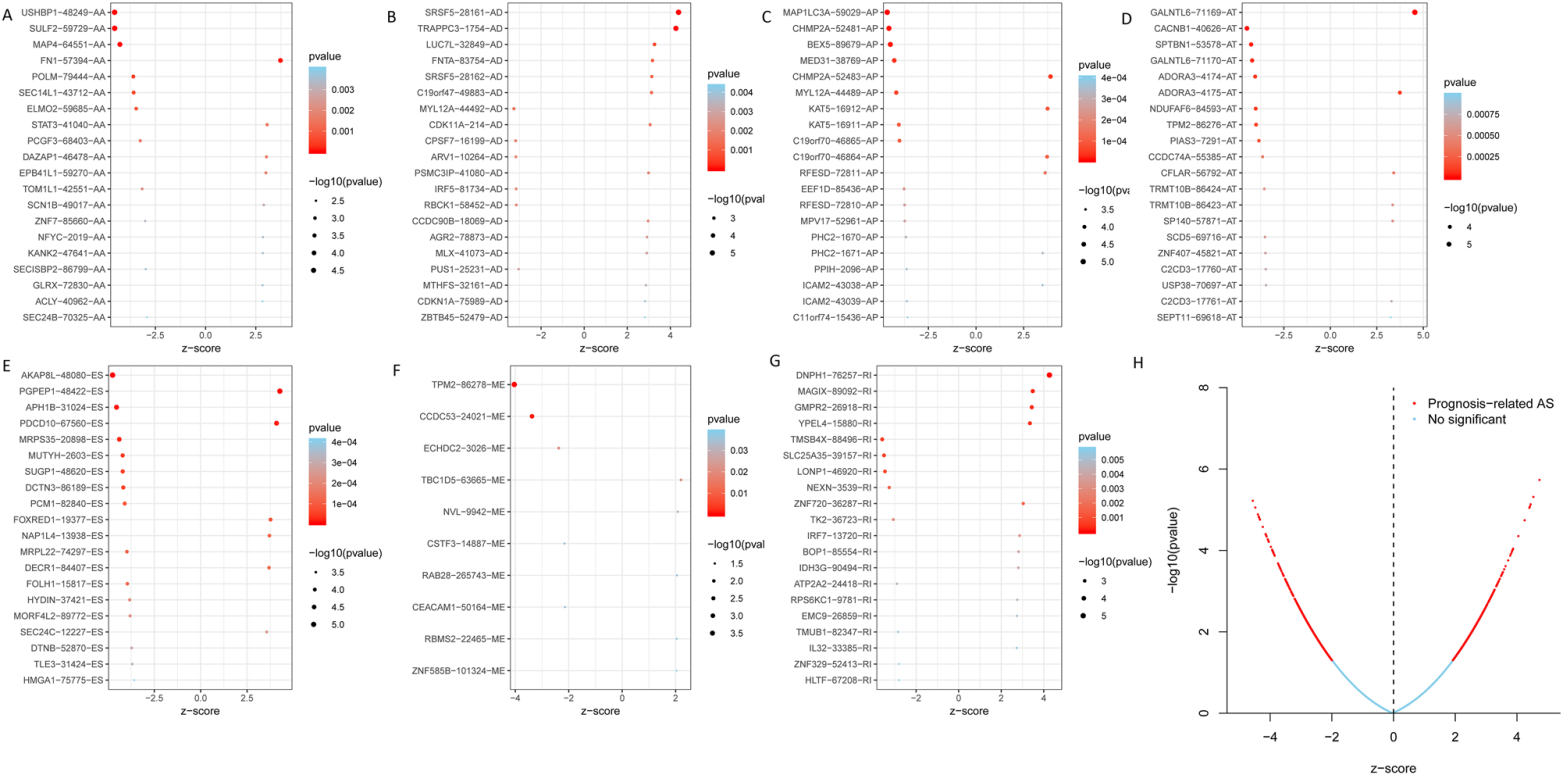
Bubble plots for subgroup analyses of survival associated ASEs in TC patients. (**A–G**) Forest plots of HRs for top 20 overall survival associated ASEs (AA, AD, AP, AT, ES, ME, and RI) in TC. (**H**) The volcano plot for prognosis-related ASEs.

**Figure 3:**
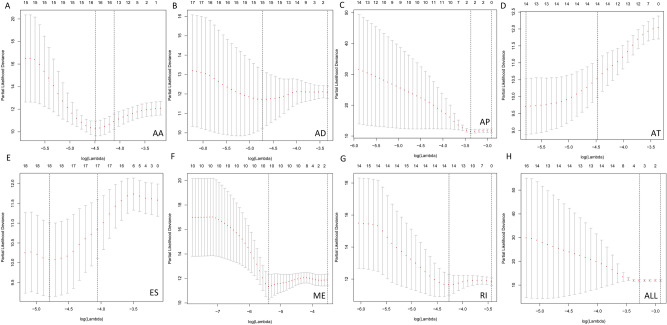
Lasso cox regression algorithm of ASEs in TC patients. (**A–H**) Lasso cox regression algorithm based cross validation plots for the seven AS events; AA, AD, AP, AT, ES, ME, RI, and ALL events in TC.

Univariate cox analysis identified 1819 ASEs which were significantly associated with OS of TC patients (Table S1, *P* < 0.05). The top 20 ASEs significantly associated with survival are shown in Fig. 2A–G. Among them, there were only 10 prognostic M events. Meanwhile, the volcano plot of prognosis-related ASEs is shown in Fig. 2H. Functional analysis and Lasso cox regression algorithm^26–28^ were used to develop a capability risk signature in order to determine the prognostic value of AS, and identify the ASEs significantly associated with survival (*P* < 0.05) (Fig. 3). Consequence analysis results indicated that there were only two AP ASEs and ES had the most ASEs (17).

### Prognostic predictors of ASEs in TCGA-TC

**Figure 4:**
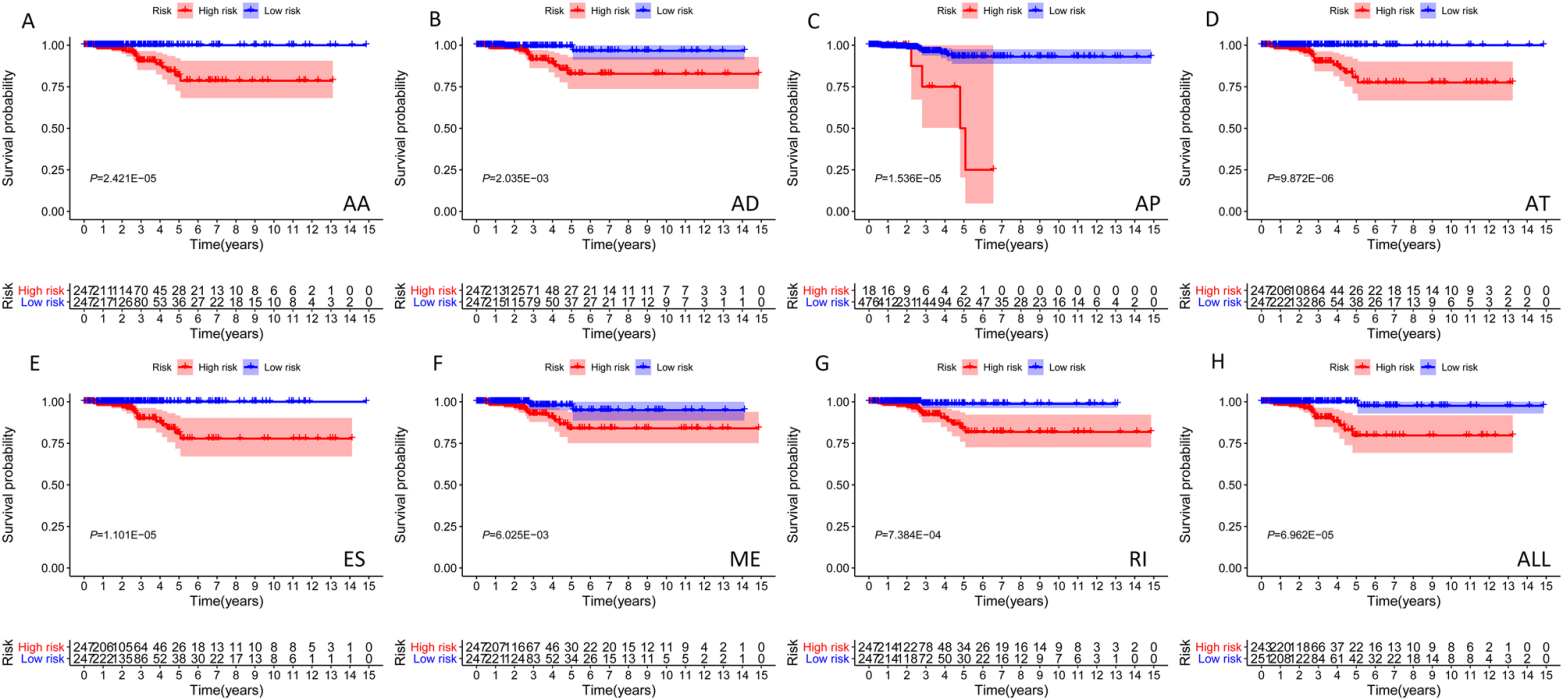
Kaplan–Meier curves for prognostic predictors of TC. (**A–G**) Kaplan–Meier plot for the survival probability over time for the prognostic predictor of the seven types of AS events in high (red) and low (blue) risk groups. (**H)** Kaplan–Meier plot for the survival probability over time for the final prognostic predictor with high (red) and low (blue) risk group.

**Figure 5:**
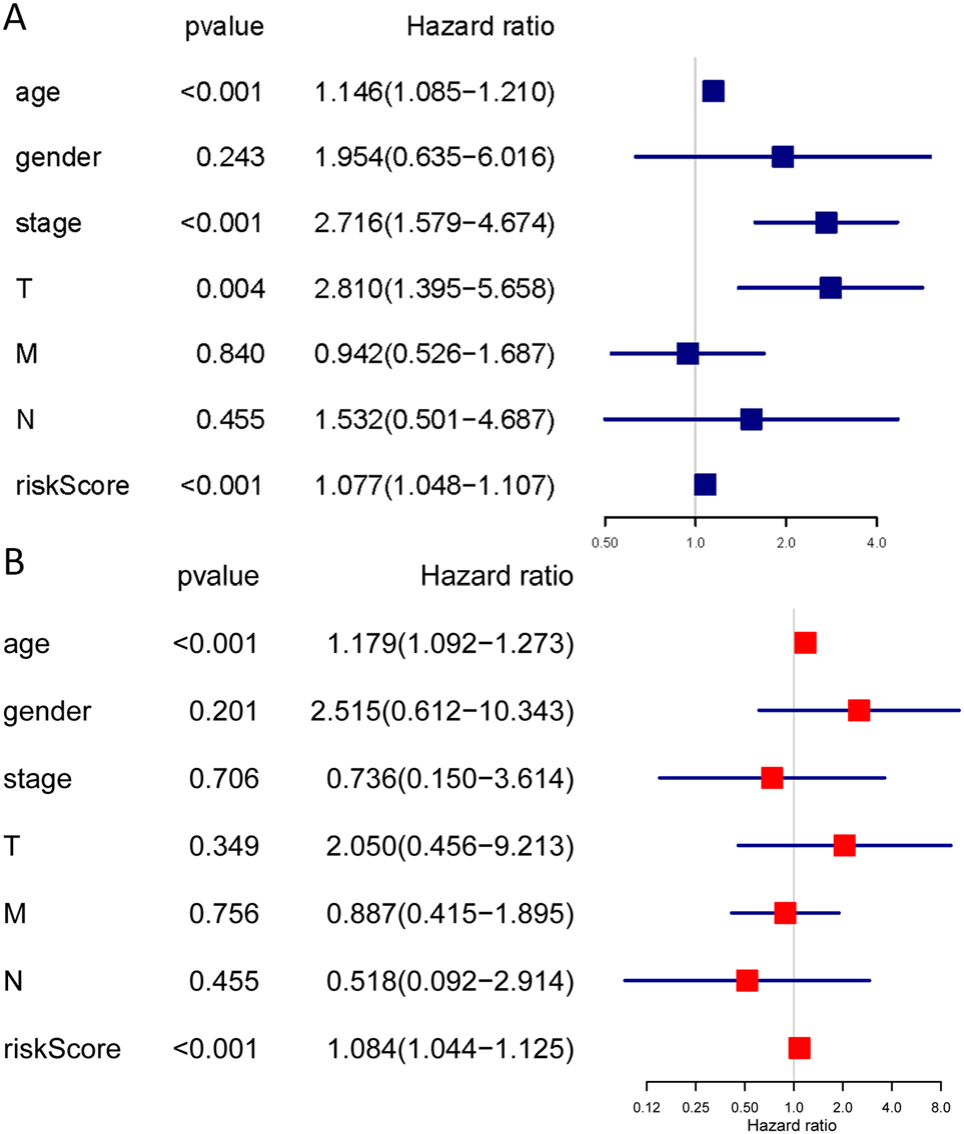
Cox regression analysis for OS-associated clinical features of ALL ASEs. (**A**) Univariate analysis; (**B**) Multivariate analysis. (*T* tumor; *N* lymph node; and *M* metastasis).

Figure 4 shows the seven prognostic models with significant prognostic predictive value for TC derived using different types of ASEs. The ROC curve revealed that AA (AUC: 0.937), AD (AUC: 0.965), AT (AUC: 0.964), ES (AUC: 0.999), ME (AUC: 0.999), and RI (AUC: 0.837) can predict the development of TC. Moreover, the integrated prediction model for TC exhibited an AUC of 0.882. Overall, aberrant active ASEs was a specific event in TC since most models exhibited a relatively high specificity value. Figures S1–S8 highlights various prognostic signatures for TC, with results showing that TC’s mortality rate was higher in the high-risk groups. Univariate and multivariate cox regression analyses indicated that the hazard ratios (HRs) for AS-ALL were 2.798 (95% CI: 2.286–3.424) and 2.603 (95% CI: 2.108–3.215), respectively (Fig. 5A,B). The samples were then divided into high‐ and low‐AS-ALL groups in order to distinguish the potential function and elucidate the significant survival difference using GSEA. GSEA results revealed that AS-ALL were mainly up-regulated in tumor and immune-related signaling pathway (Table 2).

**Table 2:** Gene sets enriched in phenotype low.

Gene set name	Size	NES	NOM p-val	FDR q-val
KEGG_APOPTOSIS	87	− 1.914	0	0.012
KEGG_LEISHMANIA_INFECTION	70	− 1.815	0	0.036
KEGG_B_CELL_RECEPTOR_SIGNALING_PATHWAY	75	− 1.772	0	0.053
KEGG_LEUKOCYTE_TRANSENDOTHELIAL_MIGRATION	116	− 1.757	0.0049	0.054
KEGG_TOLL_LIKE_RECEPTOR_SIGNALING_PATHWAY	102	− 1.750	0.0034	0.046
KEGG_RIG_I_LIKE_RECEPTOR_SIGNALING_PATHWAY	70	− 1.723	0.0034	0.062
KEGG_T_CELL_RECEPTOR_SIGNALING_PATHWAY	108	− 1.722	0.0016	0.053
KEGG_CHEMOKINE_SIGNALING_PATHWAY	188	− 1.701	0.0109	0.065
KEGG_CHRONIC_MYELOID_LEUKEMIA	73	− 1.681	0.0073	0.077
KEGG_NATURAL_KILLER_CELL_MEDIATED_CYTOTOXICITY	132	− 1.678	0.0083	0.073

*NES* normalized enrichment score; *NOM* nominal; *FDR* false discovery rate.

### Correlation between TC-ASEs and SFs expression

**Figure 6:**
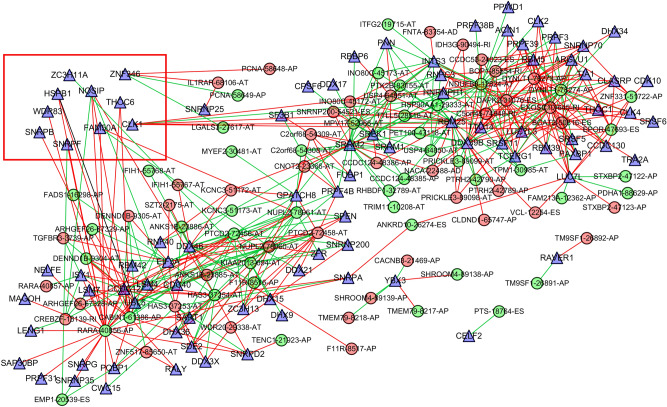
Correlation analysis between splicing factor expression and TC-ASEs. Triangles represent the splicing factors, while the oval nodes represent the TC-ASEs. Red ovals represent TC-ASEs associated with better OS, whereas the green ovals represent OS-ASEs associated with poor OS. The blue and red lines represent TC-ASEs associated with both better and poor OS.

**Figure 7:**
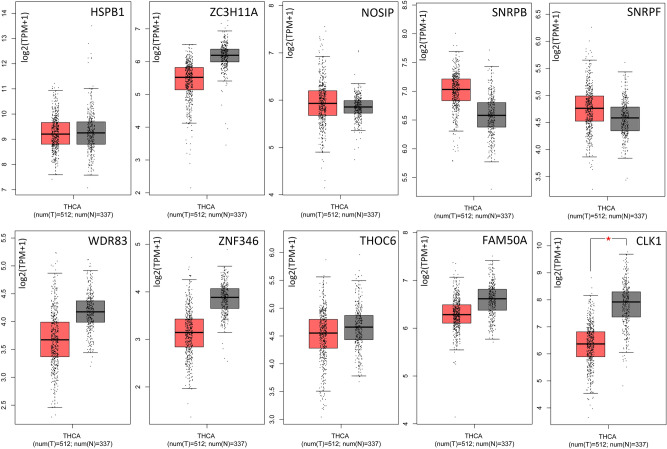
The expression of 10 SFs among normal and thyroid tumor tissues based on GEPIA^29^. The red box indicates tumor and the gray box indicates normal. We used log2 (TPM + 1) for y-axis log-scale, and one-way ANOVA for differential analysis using disease state (Tumor or Normal) as the variable for calculating differential expression. An asterisk indicates statistical significance, and each dot represents a distinct tumor or normal sample. (TPM: Transcripts Per Million).

**Figure 8:**
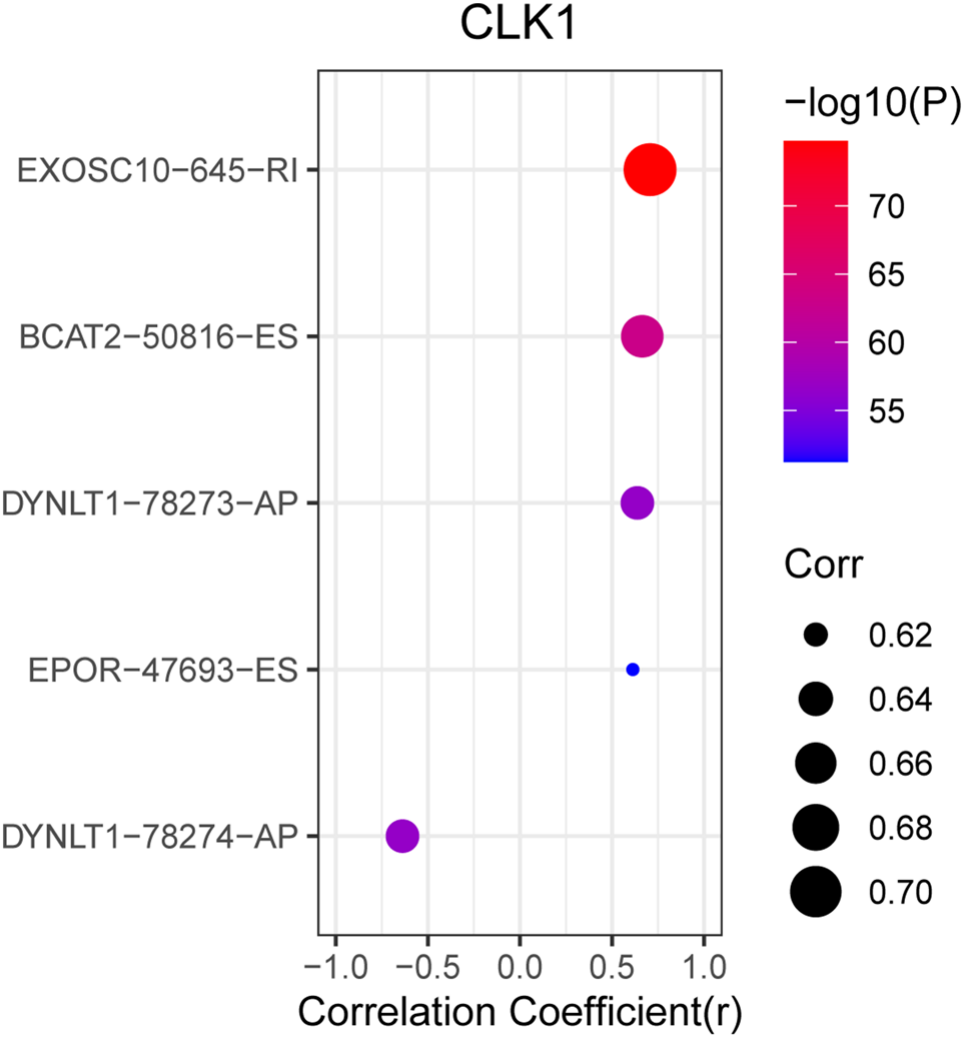
The correlation between *CLK1* and ASEs.

Univariate cox regression analysis identified 90 SFs associated with OS of TC patients (Table S2). Cytoscape correlation plots further revealed that the expression of 90 survival-associated SFs (triangular nodes) was associated with 469 TC-ASEs, of which, 260 were associated with poor OS (green ovals) and 209 were associated with better OS (red ovals). Most of the ASEs associated with better OS down-regulated the expression of SFs (blue lines), whereas most of ASEs associated with poor OS up-regulated the expression of SFs (red lines). The 10 most significant SFs were *HSPB1, ZC3H11A, NOSIP, SNRPB, SNRPF, WDR83, ZNF346, THOC6, FAM50A,* and *CLK1* (Fig. 6). SF *NOSIP* was positively correlated with the PSI of *CABIN1*-61386-AP but negatively correlated with that of *HAS3*-37253-AT. In addition, SF *ZNF346* demonstrated different connection between different ASEs types of the same gene (*PCNA*) (*P* < 0.001). This implies that SFs regulate different ASEs. The expression of these 10 SFs among normal and thyroid tumor tissues based on GEPIA are shown in Fig. 7. Our results indicated that there was a high expression of *CLK1* in normal tissue and the obtained correlation scatter plots are shown in Fig. 8. The plug-in Molecular Complex Detection (MCODE) package in Cytoscape identified *UBL5* and *PTCD2*-72456-AT as hub genes or an AS event with degrees ≥ 10.

## Discussion

Alternative splicing is a critical biological process which is involved in the production of numerous proteins. Aberrant ASEs in cancers are associated with cancer initiation and progression. Elucidation of the regulatory networks between ASEs and SFs is complicated by the fact that a gene can undergo various types of ASEs and can be regulated by a variety of SFs. This study identified ASEs and regulatory SFs in TC through the analysis of the TCGA program with the overarching goal of providing comprehensive knowledge into various RNA splicing patterns. Consequently, 1819 AS signatures were identified as being significantly associated with the OS of TC patients. Of the 469 OS-ASEs, 209 were linked with favorable OS and 260 with poor OS.

Recently, researchers have focused on aberrant regulation of AS in various tumors. Kozlovski et al.^30^ reported that AS serves as a molecular switch in many types of cancer by altering metabolism including regulation of the metabolic mTOR pathway and glycolytic pathway/TCA cycle in order to drive tumorigenesis. In addition, AS may significantly alter the coding region of drug targets, thereby leading to increased drug resistance in some cancer therapies^31^, such as BCR-ABL splice variant, imatinib resistance; BCL2-Like 11 (*BIM* or *BCL2L11*) splice variant and TKI resistance; *BRCA* splice variants leading to PARP inhibitor or cytotoxic drug resistance; and *TP53* splice variants and cisplatin resistance^32^. Over the last few years, an increasing number of AS events have been implicated in the progression of many types of cancers. *SRSF1* (also known as SF2/ASF) was the first SF to be identified as a proto-oncogene in human tumors. Studies have reported that *SRSF1* is up-regulated in various types of human tumors including colon, thyroid, breast, kidney, small intestine, and lung cancers^33,34^. Piqué et al.^35^ reported that the splicing RNA-binding protein *CELF2* is targeted by promoter hypermethylation-linked transcriptional silencing in breast cancer. On the other hand, Duan et al.^36^ proposed that aberrant splicing variants are involved in renal cell cancer. Alternative splicing was also found to regulate some apoptotic genes. Moreover, the *BCL2L1* pre-mRNA is associated with greater tumor cell survival in various cancer types including human lymphoma, breast cancer, prostate cancer, and human hepatocellular carcinoma^37,38^. A previous study found that AS of HIV-1 mRNAs increases viral coding potential, and controls the levels and timing of gene expression^39^. However, the previous studies have not fully explored the role of AS in thyroid cancer. Therefore, we aimed at elucidating the combination of splicing and clinical parameters, and potential mechanism of the survival-related ASEs in TC.

We downloaded seven types of AS from the TCGA SpliceSeq database. In total, there were 10,446 genes and 45,150 AS events in 506 TC patients, indicating that ASEs are universal in TC. Moreover, 1819 AS signatures were identified as being significantly associated with the OS of TC patients. Among the seven types of ASES, ES was the most common, followed by AP and AT. We also identified the top 20 significant survival-related ASEs of the seven AS types. Prognostic models were constructed based on risk score and ASEs types (AA, AP, AD, AT, ES, ME, RI, and ALL) in order to evaluate the diagnostic significance of aberrant ASEs in the prognosis of TC. We then plotted Kaplan–Meier survival curves of risk score and the risk scores of each type of ASEs. The results show that seven types of ASEs were associated with poor prognosis in TC patients (*P* < 0.05). We also used ROC curves to determine whether AS patterns can be used as an early predictor of the incidence of TC. Results indicated that AA (AUC: 0.937), AD (AUC: 0.965), AT (AUC: 0.964), ES (AUC: 0.999), ME (AUC: 0.999), and RI (AUC: 0.837) all had an AUC > 0.6, of which, ES and ME best predicted the incidence of TC. Moreover, the integrated predictor model of TC showed an AUC of 0.882. Cox regression was used to explore the impacts of clinicopathological parameters and risk scores on the prognosis of TC patients, with results indicating that age and risk score (All) were the risk factors for TC patients. We also investigated whether TC-ASEs are regulated by various SFs. The results showed that the expression of 90 SFs was associated with the OS of 469 ASEs in the TC cohort. The 10 most significant relations between genes and SFs were *HSPB1, ZC3H11A, NOSIP, SNRPB, SNRPF, WDR83, ZNF346, THOC6, FAM50A, and CLK1.* In addition, *UBL5* and *PTCD2*-72456-AT were identified as hub genes or AS events with degrees ≥ 10. Previous studies have reported that *UBL5* plays an evolutionarily conserved role in pre-mRNA splicing, the integrity of which is important for the fidelity of chromosome segregation^40^. Xu et al*.*^41^ found that the *PTCD2* protein regulates the processing of RNA transcripts involving cytochrome b derived from mitochondrial DNA. Our findings provide detailed information about the mechanisms through which ASEs function in TC development and progression.

## Conclusions

Although this study had some limitations (e.g. lack of therapeutic strategies, small sample size, TC subtype research, and lack of validation experiments), we have shown that ASEs are frequent in TC and are associated with patient prognosis. These ASEs may be part of a prognostic signature in TC. Our findings may provide a basis for splicing perturbations in TC and related SFs that might be implicated in these modifications. In addition, the methods used in this study can provide novel perspectives in other fields of tumor study, thereby enhancing future oncology research.

## Supplementary Information

Supplementary Information 1.

Supplementary Information 2.

Supplementary Information 3.

## Data Availability

RNA-seq data and corresponding clinical data were acquired from the data portal for TCGA (https://portal.gdc.cancer.gov/).

## Abbreviations

TC: Thyroid cancer
ASEs: Alternative splicing events
PTC: Papillary thyroid carcinoma
ATC: Anaplastic thyroid carcinoma
FTC: Follicular thyroid carcinoma
MTC: Medullary thyroid carcinoma
TCGA: Cancer genome atlas
SFs: Splicing factors
OS: Overall survival
