# Supplementary material for: Alternative splicing events implicated in carcinogenesis and prognosis of thyroid gland cancer
Source: Sci Rep. 2021 Mar 1;11:4841. doi: 10.1038/s41598-021-84403-6 (PMC7921437; doi:10.1038/s41598-021-84403-6)

Figure S1. Risk survival status plot of acceptor sites (AA) model in thyroid cancer patients.

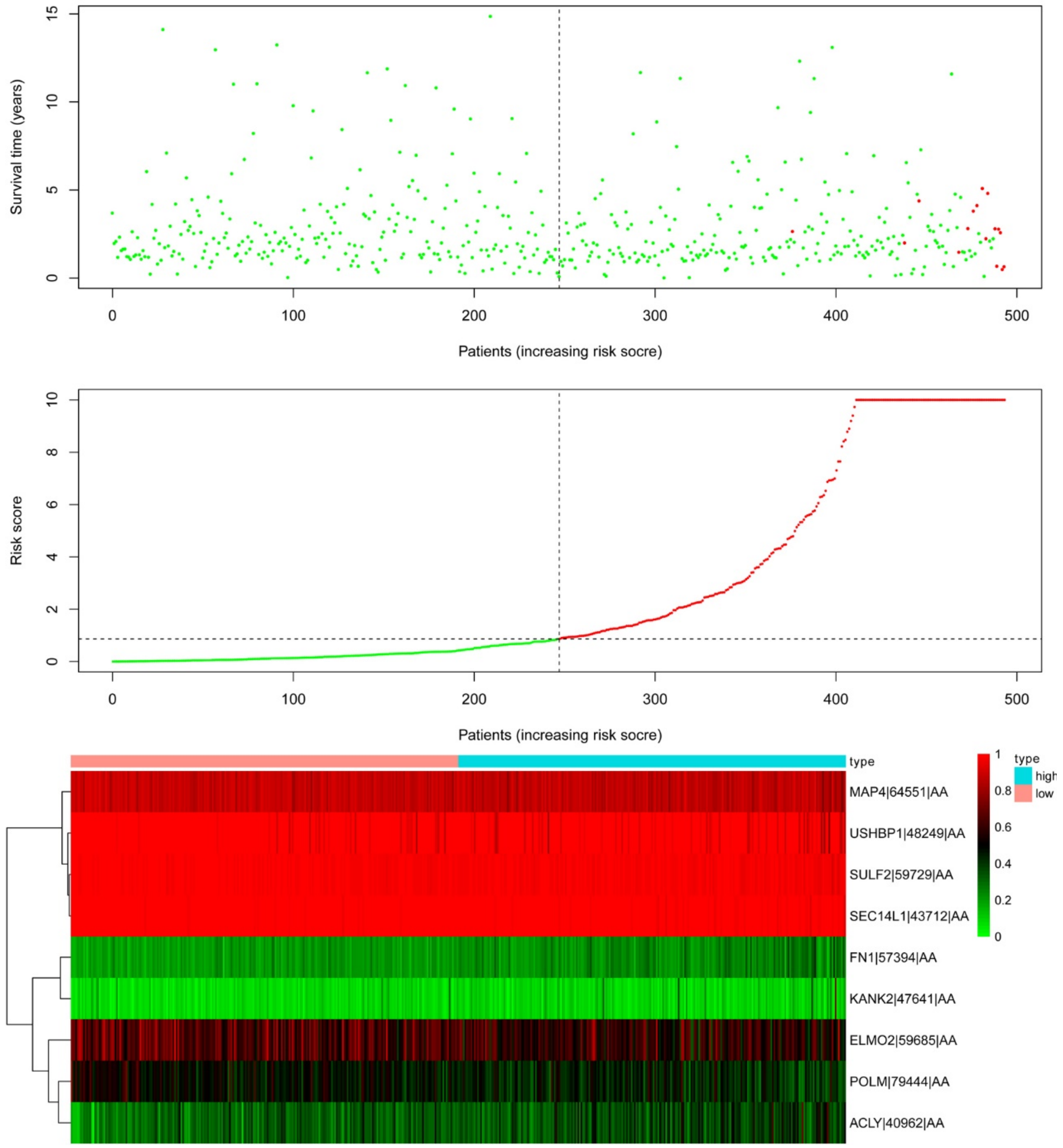

Figure S2. Risk survival status plot of alternate donor sites (AD) model in thyroid cancer patients.

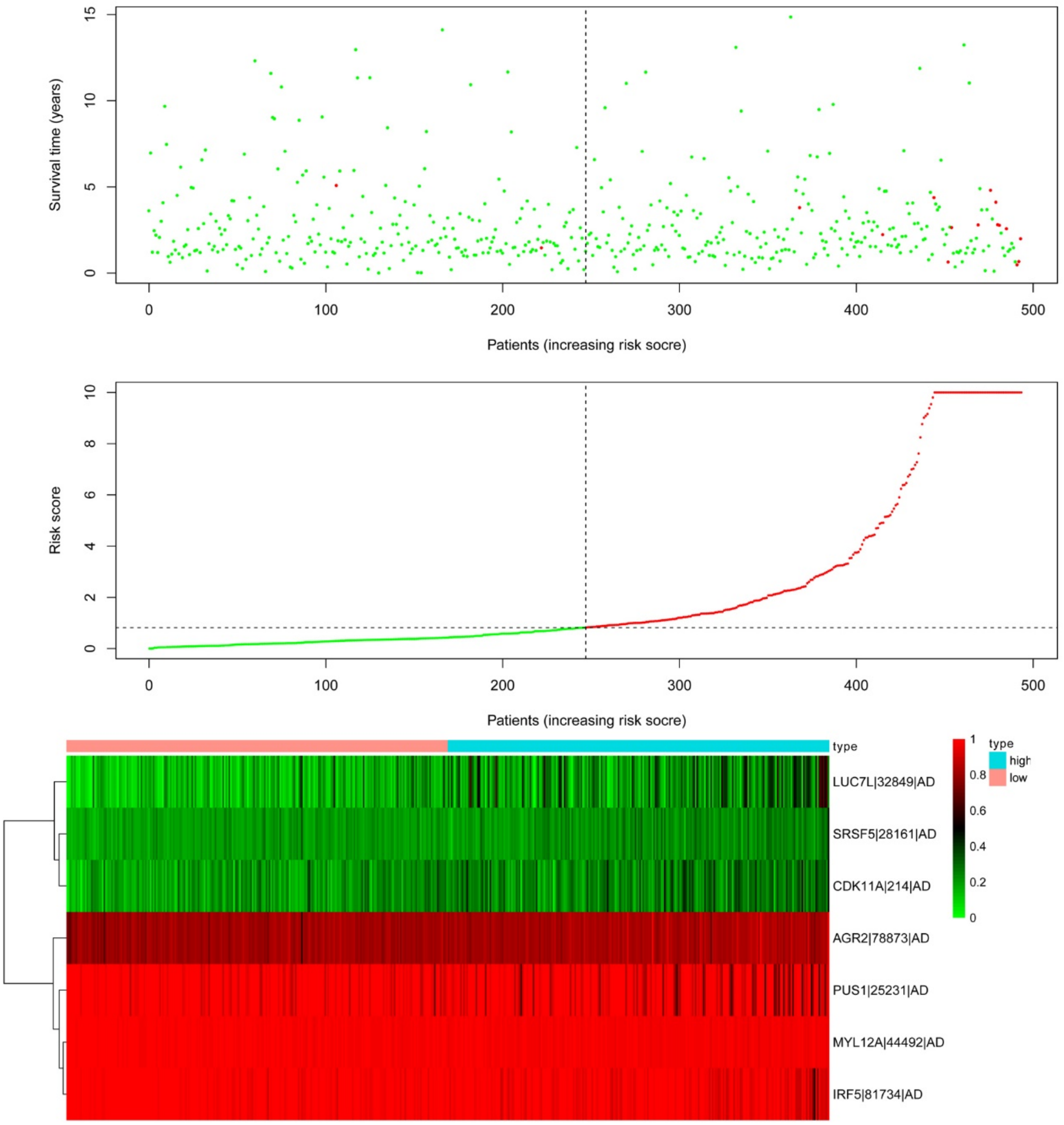

Figure S3. Risk survival status plot of alternate promoter (AP) model in thyroid cancer patients.

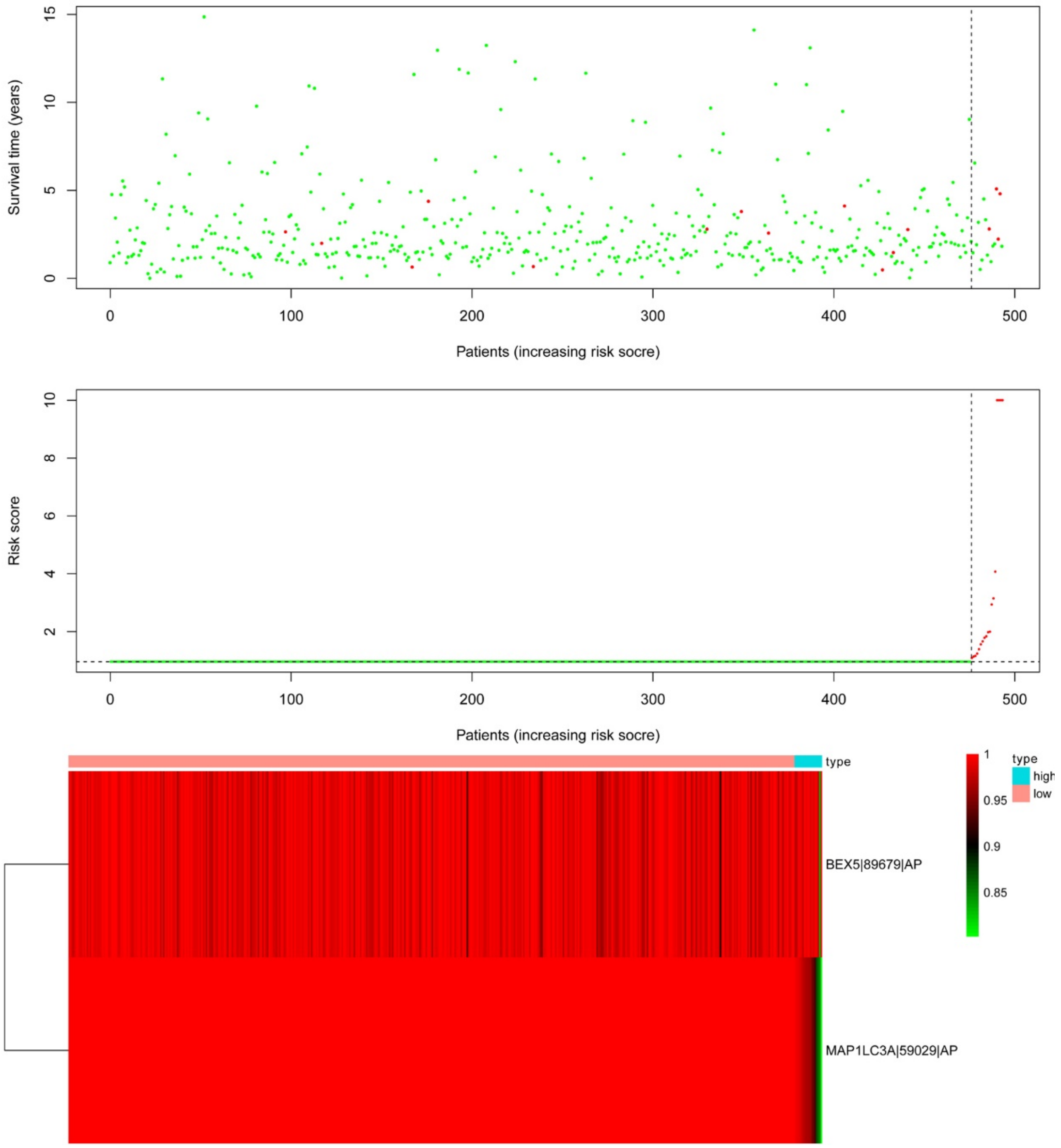

Figure S4. Risk survival status plot of alternate terminator (AT) model in thyroid cancer patients.

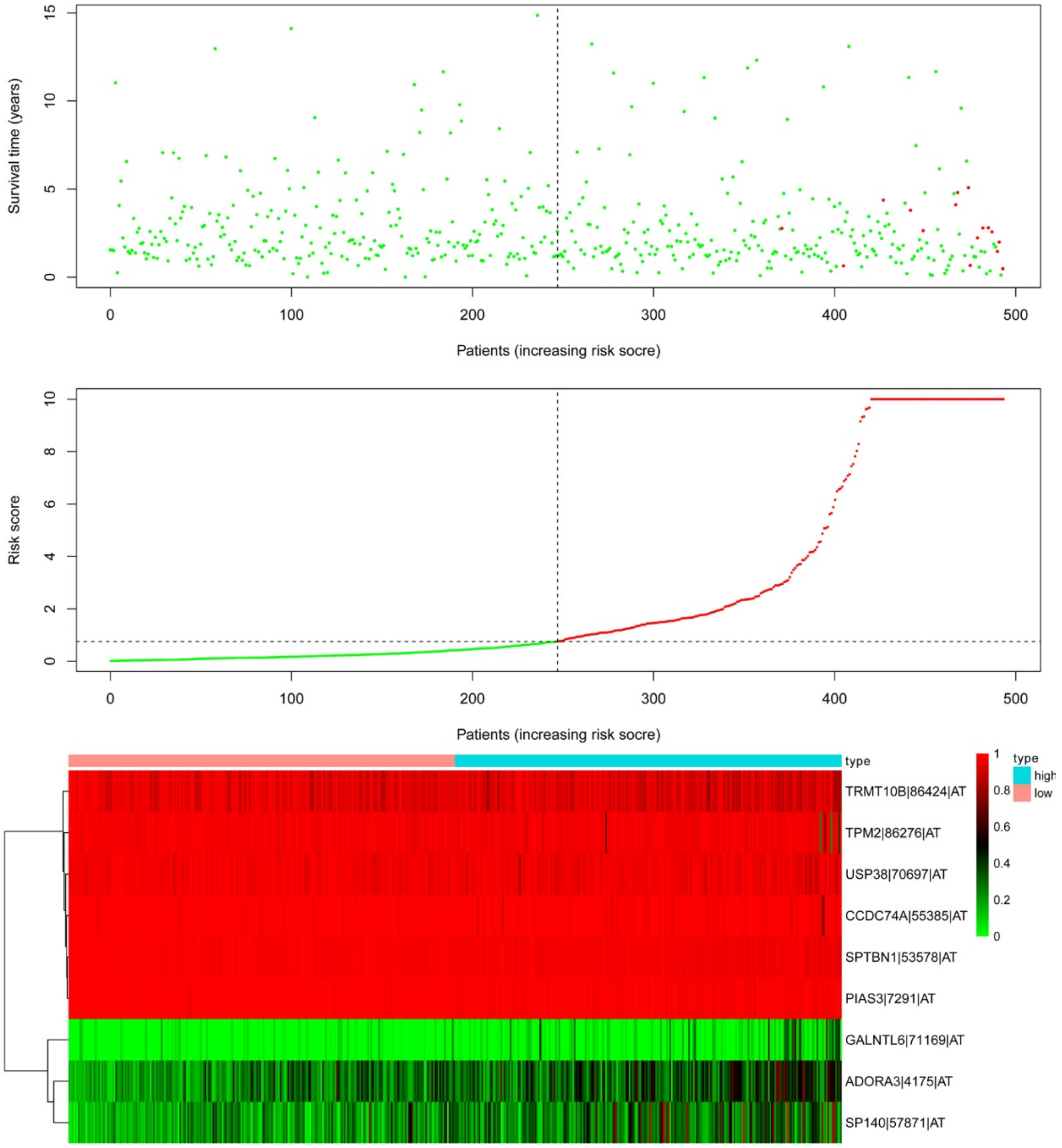

Figure S5. Risk survival status plot of exon skipping (ES) model in thyroid cancer patients.

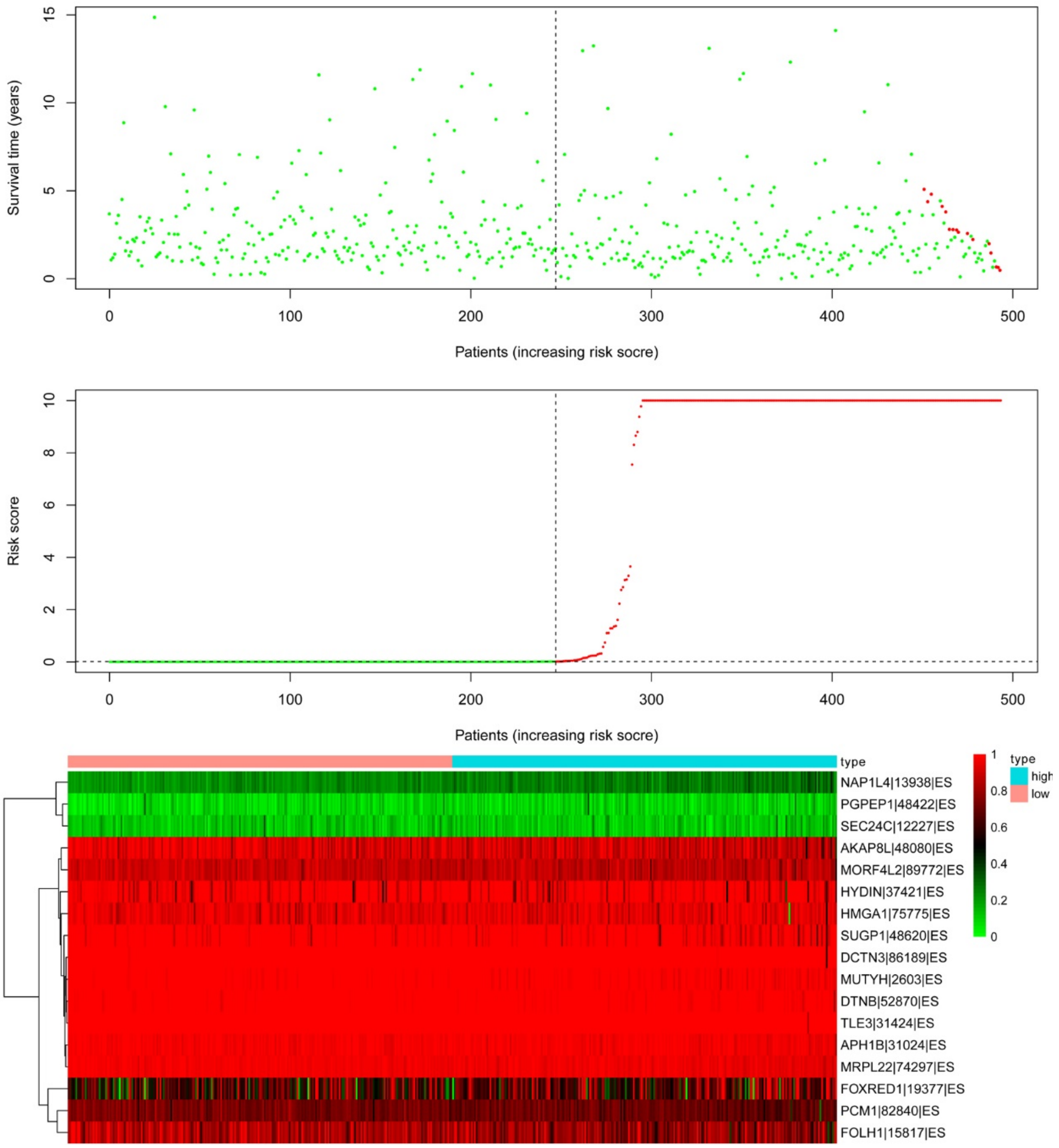

Figure S6. Risk survival status plot of exclusive exons (ME) model in thyroid cancer patients.

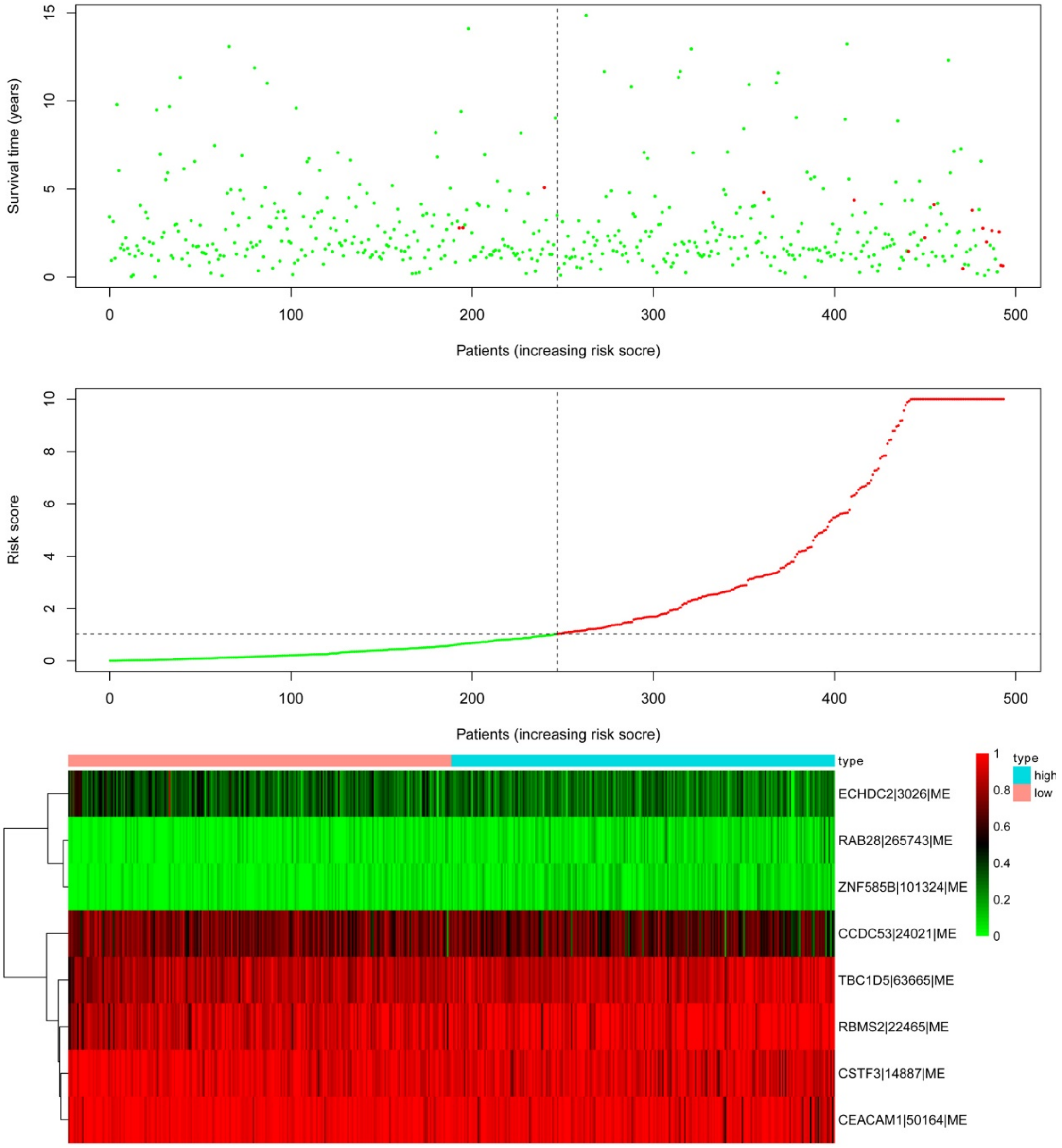

Figure S7. Risk survival status plot of retained intron (RI) model in thyroid cancer patients.

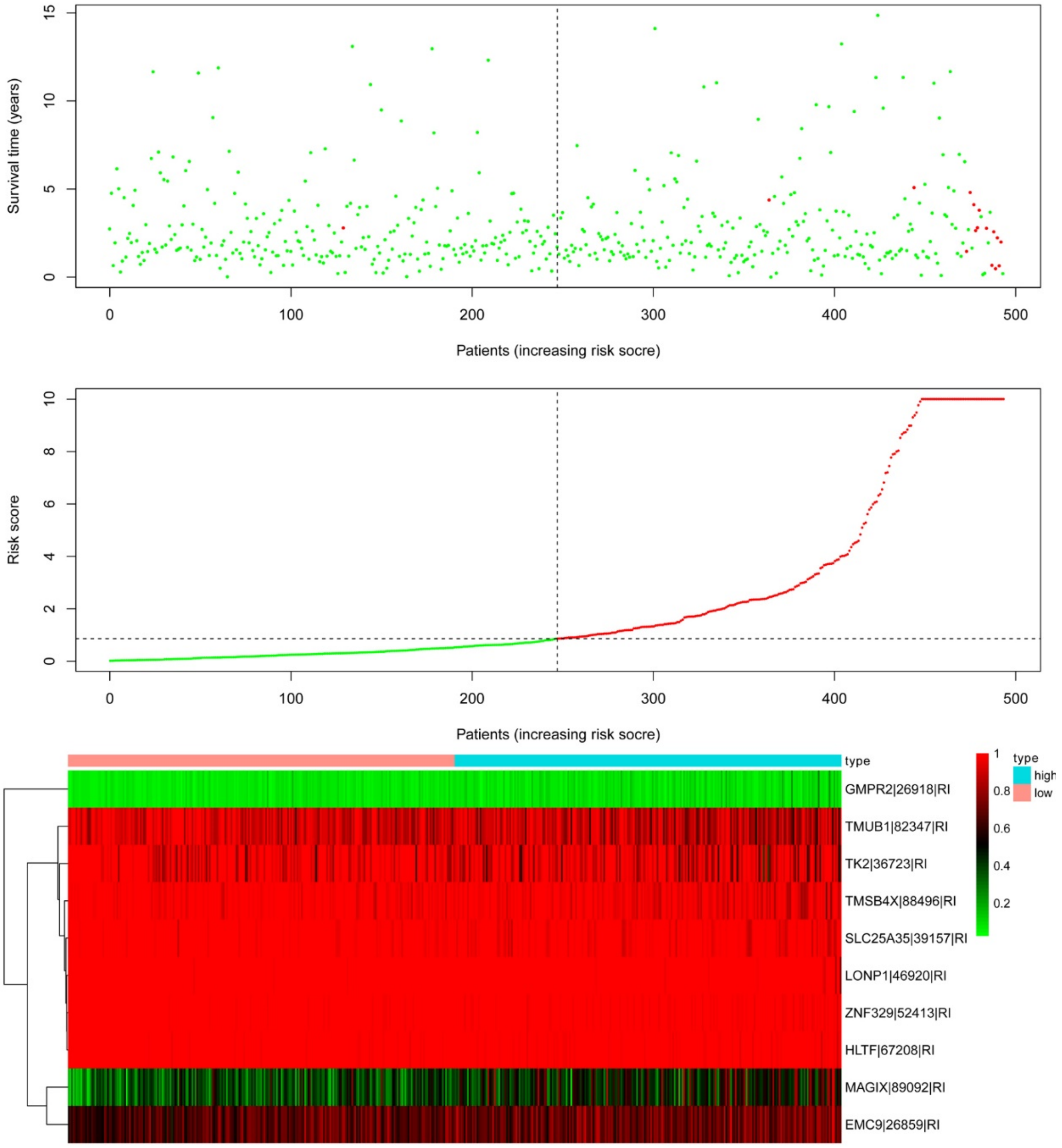

Figure S8. Risk survival status plot of all model in thyroid cancer patients.

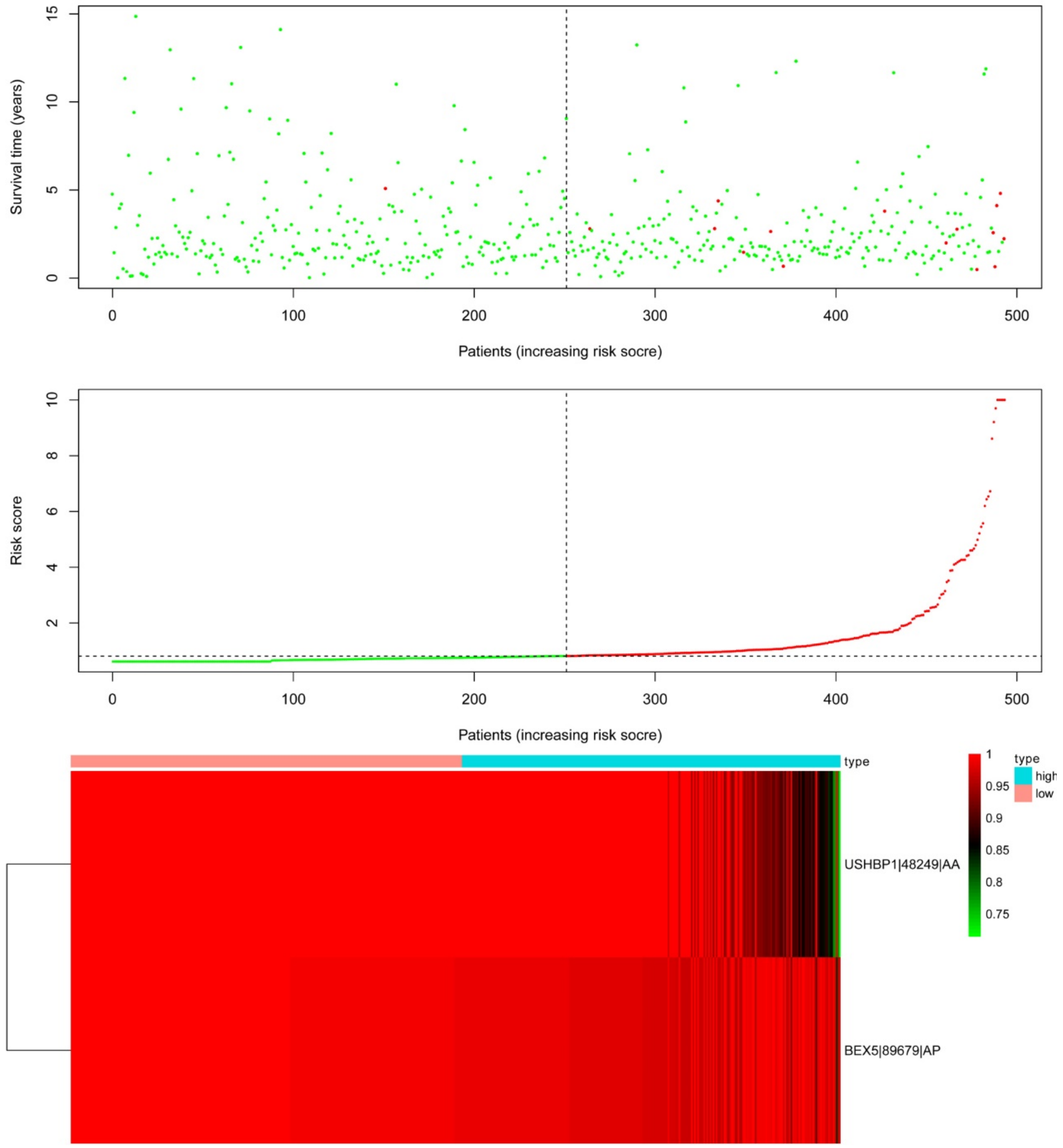

Supplement: Supplementary file 1 — Supplementary Information 1. [file 41598_2021_84403_MOESM1_ESM.pdf]
