# Supplementary material for: Alternative splicing events implicated in carcinogenesis and prognosis of thyroid gland cancer
Source: Sci Rep. 2021 Mar 1;11:4841. doi: 10.1038/s41598-021-84403-6 (PMC7921437; doi:10.1038/s41598-021-84403-6)
Supplement: Supplementary file 2 — Supplementary Information 2. [file 41598_2021_84403_MOESM2_ESM.pdf]

id	z	HR	HR.95L	HR.95H	pvalue
GALNTL6|71169|AT	4.768496	664.3407	45.95384	9604.173	1.86E-06
SRSF5|28161|AD	4.571637	1.59E+08	48524.59	5.24E+11	4.84E-06
AKAP8L|48080|ES	-4.52571	1.58E-05	1.32E-07	0.001898	6.02E-06
PGPEP1|48422|ES	4.482982	14397452	10682.59	1.94E+10	7.36E-06
DNPH1|76257|RI	4.458243	1.51E+17	4.24E+09	5.41E+24	8.26E-06
MAP1LC3A|59029|AP	-4.44407	3.83E-09	7.44E-13	1.97E-05	8.83E-06
TRAPPC3|1754|AD	4.440502	1.68E+10	515781.3	5.49E+14	8.97E-06
CHMP2A|52481|AP	-4.35995	4.05E-09	6.83E-13	2.40E-05	1.30E-05
APH1B|31024|ES	-4.33299	3.19E-16	3.13E-23	3.26E-09	1.47E-05
USHBP1|48249|AA	-4.3258	1.33E-06	2.88E-09	0.00061	1.52E-05
SULF2|59729|AA	-4.32368	8.18E-29	1.52E-41	4.41E-16	1.53E-05
CACNB1|40626|AT	-4.30182	0.000154	2.81E-06	0.008397	1.69E-05
BEX5|89679|AP	-4.29459	9.55E-10	7.30E-14	1.25E-05	1.75E-05
PDCD10|67560|ES	4.28643	4369.352	94.58917	201833.2	1.82E-05
MRPS35|20898|ES	-4.20204	6.28E-16	5.09E-23	7.74E-09	2.65E-05
MED31|38769|AP	-4.11002	8.38E-18	6.03E-26	1.17E-09	3.96E-05
SPTBN1|53578|AT	-4.09106	1.43E-31	2.40E-46	8.57E-17	4.29E-05
CHMP2A|52483|AP	4.082702	4.38E+08	31128.6	6.17E+12	4.45E-05
MAP4|64551|AA	-4.07677	2.16E-09	1.47E-13	3.17E-05	4.57E-05
GALNTL6|71170|AT	-4.04031	0.002844	0.000166	0.04887	5.34E-05
MUTYH|2603|ES	-4.03795	4.96E-11	4.93E-16	4.98E-06	5.39E-05
SUGP1|48620|ES	-4.03559	6.27E-05	5.71E-07	0.006894	5.45E-05
MYL12A|44489|AP	-4.01658	0.001133	4.14E-05	0.031022	5.90E-05
DCTN3|86189|ES	-4.0079	1.18E-05	4.61E-08	0.003036	6.13E-05
PCM1|82840|ES	-3.93907	2.96E-05	1.65E-07	0.005302	8.18E-05
FOXRED1|19377|ES	3.914589	468.3061	21.54982	10176.91	9.06E-05
FN1|57394|AA	3.903323	62738494	7623.973	5.16E+11	9.49E-05
KAT5|16912|AP	3.902028	22060.32	145.1587	3352590	9.54E-05
KAT5|16911|AP	-3.89967	4.54E-05	2.98E-07	0.006916	9.63E-05
ADORA3|4175|AT	3.892528	526.7705	22.4508	12359.79	9.92E-05
ADORA3|4174|AT	-3.89253	0.001898	8.09E-05	0.044542	9.92E-05
TPM2|86278|ME	-3.88442	0.00411	0.000257	0.065737	0.000103
C19orf70|46865|AP	-3.87183	2.49E-11	1.07E-16	5.80E-06	0.000108
C19orf70|46864|AP	3.871695	4.02E+10	172229.5	9.39E+15	0.000108
NDUFAF6|84593|AT	-3.86624	2.38E-23	8.09E-35	7.01E-12	0.000111
TPM2|86276|AT	-3.84791	0.005143	0.000351	0.07534	0.000119
NAP1L4|13938|ES	3.845235	2.77E+08	13788.68	5.58E+12	0.00012
MRPL22|74297|ES	-3.83525	6.57E-16	1.14E-23	3.77E-08	0.000125
DECR1|84407|ES	3.818454	119.2356	10.24739	1387.391	0.000134
FOLH1|15817|ES	-3.81746	0.00068	1.61E-05	0.028762	0.000135
RFESD|72811|AP	3.751415	54.03907	6.721054	434.4885	0.000176
HYDIN|37421|ES	-3.7082	0.007876	0.000609	0.10191	0.000209
PIAS3|7291|AT	-3.70772	2.32E-26	6.51E-40	8.24E-13	0.000209
MORF4L2|89772|ES	-3.69012	2.80E-06	3.14E-09	0.00249	0.000224
SEC24C|12227|ES	3.66828	156142.4	262.1923	92986883	0.000244
EEF1D|85436|AP	-3.66804	3.12E-08	3.05E-12	0.00032	0.000244
RFESD|72810|AP	-3.64609	0.019659	0.002378	0.162504	0.000266
MPV17|52961|AP	-3.63823	9.46E-14	9.12E-21	9.82E-07	0.000275
DTNB|52870|ES	-3.6268	1.44E-12	5.75E-19	3.61E-06	0.000287
MAGIX|89092|RI	3.622995	137.6679	9.588907	1976.497	0.000291
TLE3|31424|ES	-3.59974	3.61E-11	7.44E-17	1.75E-05	0.000319
PHC2|1670|AP	-3.5965	0.00273	0.000109	0.068136	0.000323
PHC2|1671|AP	3.58153	363.3206	14.42752	9149.31	0.000342
GMPR2|26918|RI	3.574447	3545390	908.4677	1.38E+10	0.000351
PPIH|2096|AP	-3.56651	5.17E-09	1.45E-13	0.000185	0.000362
ICAM2|43038|AP	3.560748	33818.82	108.6791	10523755	0.00037
ICAM2|43039|AP	-3.56071	2.96E-05	9.50E-08	0.009202	0.00037

C11orf74|15436|AP	-3.53995	4.97E-05	2.06E-07	0.012002	0.0004
C11orf74|15435|AP	3.539924	20110.13	83.31554	4854044	0.0004
HMGA1|75775|ES	-3.53511	0.030435	0.00439	0.210976	0.000408
FAM84A|52720|ES	3.534721	2157.861	30.57341	152301.1	0.000408
CCDC74A|55385|AT	-3.53367	4.80E-06	5.38E-09	0.004277	0.00041
LDHA|14634|ES	-3.52517	9.76E-09	3.43E-13	0.000277	0.000423
CFLAR|56792|AT	3.523132	1.82E+13	764352.2	4.33E+20	0.000426
YPEL4|15880|RI	3.48036	11.69854	2.928286	46.7358	0.000501
PLEKHA4|50820|ES	-3.47873	0.000279	2.77E-06	0.028065	0.000504
MORF4L2|89774|ES	-3.47483	0.004597	0.000221	0.095707	0.000511
MASTL|11063|ES	-3.4705	0.000368	4.23E-06	0.032	0.000519
POLM|79444|AA	-3.4578	4.54E-05	1.57E-07	0.013149	0.000545
TMSB4X|88496|RI	-3.45766	1.26E-05	2.10E-08	0.00754	0.000545
TRMT10B|86424|AT	-3.45204	1.81E-07	2.69E-11	0.001218	0.000556
TRMT10B|86423|AT	3.452011	5526526	820.8418	3.72E+10	0.000556
SP140|57871|AT	3.449762	41.9161	5.019192	350.0483	0.000561
SEC14L1|43712|AA	-3.44294	9.64E-24	7.61E-37	1.22E-10	0.000575
PTS|18764|ES	-3.43826	5.56E-09	1.09E-13	0.000282	0.000585
PLCB2|29965|ES	-3.43404	1.46E-05	2.55E-08	0.008408	0.000595
C11orf74|15441|ES	3.43381	138.9292	8.312296	2322.019	0.000595
RAP1A|4176|AP	3.424157	3124.737	31.21806	312767	0.000617
SCD5|69716|AT	-3.41915	9.82E-11	1.80E-16	5.36E-05	0.000628
IQCA1|58093|ES	-3.40816	3.00E-07	5.31E-11	0.001691	0.000654
SCRN2|42115|AP	-3.40204	1.38E-05	2.18E-08	0.008699	0.000669
SCRN2|42116|AP	3.402036	72614.97	114.9518	45870820	0.000669
ZNF407|45821|AT	-3.40116	2.78E-16	3.02E-25	2.56E-07	0.000671
MKRN1|81998|AP	-3.39559	3.81E-09	5.26E-14	0.000276	0.000685
DENND3|85329|ES	-3.38896	2.55E-06	1.49E-09	0.004381	0.000702
SHROOM4|89139|AP	3.388611	14.70205	3.105772	69.59635	0.000702
C2CD3|17760|AT	-3.38627	1.04E-06	3.58E-10	0.00302	0.000709
LUC7L|32849|AD	3.380954	135.5039	7.871245	2332.707	0.000722
SLC25A35|39157|RI	-3.37871	4.60E-09	6.71E-14	0.000316	0.000728
USP38|70697|AT	-3.37665	1.11E-08	2.67E-13	0.000459	0.000734
TM4SF18|67217|AP	3.376637	5264.799	36.41759	761118.9	0.000734
C2CD3|17761|AT	3.373388	820706.5	300.6074	2.24E+09	0.000742
CENPM|62469|ES	-3.37302	7.98E-07	2.28E-10	0.002789	0.000743
BTBD11|24190|AP	3.368431	39.19647	4.636562	331.3583	0.000756
MMAB|24330|ES	3.366334	43.5433	4.838382	391.8704	0.000762
MORF4L2|89771|ES	-3.3629	0.011637	0.000868	0.155996	0.000771
LONP1|46920|RI	-3.33785	1.55E-11	6.94E-18	3.45E-05	0.000844
CYB5R2|14205|AP	-3.33388	0.000525	6.19E-06	0.044493	0.000856
TPM3|7785|AP	-3.32924	4.66E-05	1.31E-07	0.01653	0.000871
ELP2|45235|ES	-3.32841	3.64E-05	8.85E-08	0.014958	0.000873
ELMO2|59685|AA	-3.32832	0.000444	4.72E-06	0.041863	0.000874
DPYSL3|73958|AP	3.313645	68.95911	5.637625	843.5038	0.000921
DPYSL3|73957|AP	-3.31288	0.014507	0.001186	0.177511	0.000923
NSL1|9746|ES	3.311022	34779.74	71.29877	16965658	0.00093
VPS13D|706|AP	3.305177	18.54388	3.282161	104.7711	0.000949
SEPT11|69618|AT	3.297266	1.44E+10	13179.85	1.57E+16	0.000976
CCDC91|99554|ES	-3.28343	1.22E-05	1.43E-08	0.010463	0.001026
ATG13|15599|ES	3.28174	14205.1	47.03991	4289651	0.001032
FNTA|83754|AD	3.278049	34188.86	66.53491	17567898	0.001045
NAT9|43295|ES	-3.27394	1.10E-11	3.02E-18	4.00E-05	0.001061
FN1|57397|ES	3.273291	1242.332	17.43758	88509.38	0.001063
XRN2|58833|AP	-3.27191	4.40E-15	1.10E-23	1.75E-06	0.001068
WDR6|64798|ES	-3.26906	2.54E-07	2.82E-11	0.002286	0.001079
DMKN|49196|ES	-3.26711	4.70E-05	1.19E-07	0.018549	0.001087
VPS13D|704|AP	-3.26588	0.011487	0.000787	0.167625	0.001091

MMADHC|55560|AP	-3.26524	2.78E-08	8.09E-13	0.000954	0.001094
NFATC1|46245|ES	-3.26387	1.04E-11	2.63E-18	4.09E-05	0.001099
ARIH1|31555|ES	-3.25646	1.65E-17	1.31E-27	2.08E-07	0.001128
CYB5R2|14204|AP	3.254309	1702.966	19.28219	150402.8	0.001137
SHROOM4|89138|AP	-3.25314	0.075395	0.015884	0.35787	0.001141
CASP8|56824|ES	-3.25207	0.000163	8.51E-07	0.031272	0.001146
CCDC53|24021|ME	-3.24934	0.009606	0.000583	0.15829	0.001157
CKAP2|26001|AT	-3.24456	1.71E-09	8.62E-15	0.000338	0.001176
ERG|60587|AP	3.244089	173.8469	7.70437	3922.804	0.001178
SRSF5|28162|AD	3.243106	55432.99	75.31511	40799477	0.001182
SEPT11|69616|AT	-3.23706	1.51E-10	1.71E-16	0.000133	0.001208
ZNF223|50275|ES	3.235686	16.52802	3.022062	90.39374	0.001214
WDR74|16457|ES	-3.23518	8.65E-24	9.22E-38	8.11E-10	0.001216
TPM1|30979|AP	3.234242	98.99982	6.113245	1603.234	0.00122
ARMCX4|89644|AP	3.231204	46.23641	4.519063	473.0639	0.001233
C19orf47|49883|AD	3.228169	443.5202	10.96113	17946.15	0.001246
STXBP5|78057|ES	3.227956	36.19022	4.094862	319.8476	0.001247
ATP5C1|10726|ES	-3.22362	1.33E-07	8.74E-12	0.002014	0.001266
TMEM161B|72731|AT	-3.22229	0.000915	1.30E-05	0.06452	0.001272
TMEM161B|72730|AT	3.222242	1092.471	15.49757	77011.57	0.001272
S100A5|7709|AP	3.217233	42.7841	4.340025	421.767	0.001294
ANK3|11842|AP	-3.21679	0.024276	0.002519	0.233925	0.001296
IL1R1|54773|AP	3.214664	152.0707	7.106632	3254.071	0.001306
ARMCX4|89643|AP	-3.21264	0.021793	0.002111	0.224943	0.001315
UGP2|53757|ES	3.205183	6924.188	31.04489	1544357	0.00135
DIP2A|60939|AT	3.199402	77546022	1138.649	5.28E+12	0.001377
PDE4D|72138|AP	3.194542	32.28732	3.829901	272.1926	0.001401
NR1H3|15694|AP	3.181302	3172.586	22.09185	455611.6	0.001466
STAT3|41040|AA	3.173961	12244.19	36.60817	4095263	0.001504
SMIM12|1697|AP	-3.17044	1.92E-14	6.35E-23	5.79E-06	0.001522
MYL12A|44492|AD	-3.16571	1.97E-23	1.73E-37	2.25E-09	0.001547
MAGIX|89091|ES	-3.16393	0.01353	0.000941	0.194492	0.001557
TM4SF18|67218|AP	-3.15886	0.000277	1.71E-06	0.044622	0.001584
CDK11A|214|AD	3.15882	1064.059	14.08684	80374.38	0.001584
NEXN|3539|RI	-3.15421	1.40E-05	1.35E-08	0.014525	0.001609
LCMT1|35668|ES	-3.1468	2.97E-28	2.13E-45	4.15E-11	0.001651
EIF4H|80063|ES	3.145897	13427371	486.5115	3.71E+11	0.001656
PCGF3|68403|AA	-3.14541	8.96E-09	8.67E-14	0.000927	0.001659
AUTS2|79911|ES	-3.14458	0.001287	2.03E-05	0.081501	0.001663
FAM65B|75542|AT	-3.14105	0.001436	2.42E-05	0.085327	0.001683
ZNF720|36287|RI	3.138432	399.1222	9.477733	16807.66	0.001699
PLK3|2578|ES	-3.13796	5.22E-06	2.62E-09	0.0104	0.001701
COQ2|69778|ES	-3.13619	1.14E-20	3.92E-33	3.31E-08	0.001712
DAZAP1|46478|AA	3.135122	2.31E+11	18187.18	2.94E+18	0.001718
BCAS3|42872|ES	-3.13507	0.000155	6.41E-07	0.037296	0.001718
SMAP1|76647|ES	3.132244	546.9766	10.58566	28263.09	0.001735
LUC7L|32850|ES	3.128692	36.32551	3.826658	344.8289	0.001756
CCDC66|65343|AT	-3.12747	0.000618	6.02E-06	0.063395	0.001763
FN1|57396|ES	3.126625	4506.673	23.08825	879672.5	0.001768
INADL|3238|AT	3.117651	127319	78.6349	2.06E+08	0.001823
FGD4|21032|AT	-3.11437	7.89E-08	2.67E-12	0.002329	0.001843
USP36|43913|AT	-3.10662	3.45E-06	1.24E-09	0.009641	0.001892
EPB41L1|59270|AA	3.105602	1173.03	13.55969	101477.1	0.001899
RGS6|28207|AT	-3.09686	4.79E-08	1.11E-12	0.002055	0.001956
FAM129B|87639|AP	-3.0933	2.40E-17	7.07E-28	8.14E-07	0.001979
CPSF7|16199|AD	-3.09013	5.80E-161	1.35E-262	2.49E-59	0.002001
ARV1|10264|AD	-3.08102	1.15E-38	8.47E-63	1.57E-14	0.002063
PSMC3IP|41080|AD	3.078103	43.16368	3.926147	474.5374	0.002083

USHBP1|48250|ES	-3.07234	0.000274	1.46E-06	0.05132	0.002124
GSTT1|61366|ES	-3.07168	1.25E-14	1.67E-23	9.28E-06	0.002129
MORF4L2|89775|ES	-3.07139	1.50E-07	6.62E-12	0.003392	0.002131
IRF5|81734|AD	-3.06858	3.35E-05	4.64E-08	0.024168	0.002151
SULT1A1|35820|ES	3.068368	172.8294	6.431425	4644.381	0.002152
KLHL7|78948|AP	-3.06747	1.03E-08	8.16E-14	0.001308	0.002159
PHF19|87400|AP	3.067154	61.6975	4.428493	859.5657	0.002161
TOM1L1|42551|AA	-3.0625	4.81E-05	8.28E-08	0.027886	0.002195
RBCK1|58452|AD	-3.06226	4.77E-11	1.18E-17	0.000193	0.002197
S100A5|7710|AP	-3.06165	0.026557	0.002602	0.270997	0.002201
EIF3E|84876|ES	-3.06145	0.055145	0.008626	0.352535	0.002203
PHF19|87399|AP	-3.05789	0.01632	0.001167	0.228181	0.002229
CCDC90B|18069|AD	3.055456	17695.22	33.34349	9390764	0.002247
MTMR11|7415|ES	-3.05506	2.29E-07	1.26E-11	0.00417	0.00225
GPR97|36593|AT	-3.05214	2.24E-08	2.75E-13	0.001832	0.002272
NAPA|50659|ES	-3.05162	4.75E-32	3.62E-52	6.23E-12	0.002276
TMEM66|83266|ES	-3.04724	0.000806	8.26E-06	0.078743	0.002309
MAP2|57224|ES	-3.04195	0.000662	5.93E-06	0.074016	0.002351
USP4|64850|AT	-3.03956	3.14E-10	2.34E-16	0.000422	0.002369
WWP2|37322|ES	3.037062	1186.071	12.30942	114283.5	0.002389
DNAJC19|67760|ES	-3.03686	4.66E-08	8.64E-13	0.002513	0.002391
CITED1|89450|AP	3.033108	26421.66	36.68879	19027728	0.00242
SOX7|82607|AP	-3.03294	0.00201	3.63E-05	0.111156	0.002422
GUSB|79857|ES	-3.02929	0.001622	2.54E-05	0.103562	0.002451
ELMO1|79267|AP	-3.02902	0.031754	0.003407	0.295956	0.002453
CREB1|57176|AT	-3.02739	2.17E-12	6.10E-20	7.72E-05	0.002467
FLNB|65418|ES	-3.02698	2.00E-17	3.07E-28	1.30E-06	0.00247
PRSS23|18202|AT	3.017357	20743.39	32.56819	13211914	0.00255
PRSS23|18201|AT	-3.01618	4.83E-05	7.58E-08	0.030807	0.00256
PLCD4|57506|ES	-3.01593	0.044739	0.00594	0.336948	0.002562
SPTBN4|49912|AT	3.015783	130.1044	5.498053	3078.756	0.002563
ELMO1|79266|AP	3.014488	13.52601	2.487162	73.55889	0.002574
ZNF415|51674|ES	3.006671	33.13955	3.382794	324.6518	0.002641
TPM1|30980|AP	-3.00657	0.00971	0.000473	0.199225	0.002642
ACOT8|59626|ES	3.005682	28816.53	35.60989	23319156	0.00265
RAP1A|4178|AP	-3.00485	0.000779	7.32E-06	0.083011	0.002657
ZDHHC4|78750|ES	3.000422	95661.12	53.35466	1.72E+08	0.002696
CABIN1|61387|AP	2.998667	305.4924	7.257296	12859.55	0.002712
C8orf59|84338|ES	2.998652	2901915	173.2115	4.86E+10	0.002712
SOX7|82608|AP	2.997333	457.885	8.334528	25155.43	0.002724
AGO3|1741|AT	-2.99725	0.000276	1.30E-06	0.058622	0.002724
AGR2|78873|AD	2.996036	556527.5	97.02236	3.19E+09	0.002735
SPATA9|72817|AT	-2.99155	0.022075	0.001815	0.268485	0.002776
SPATA9|72815|AT	2.990047	45.24301	3.718296	550.5021	0.002789
GRAMD3|73157|AP	-2.98803	4.93E-06	1.63E-09	0.014927	0.002808
FBXW7|70849|AP	-2.98788	0.019337	0.001453	0.257316	0.002809
CEP57|18385|AP	-2.98519	7.45E-05	1.45E-07	0.038224	0.002834
MLX|41073|AD	2.984921	1.25E+08	602.4397	2.58E+13	0.002837
PAM16|33651|AT	-2.98051	4.50E-11	7.06E-18	0.000286	0.002878
PAM16|33652|AT	2.980474	2.22E+10	3490.029	1.42E+17	0.002878
STXBP2|47123|AP	2.979008	76.17515	4.402722	1317.97	0.002892
STXBP2|47122|AP	-2.97901	0.013128	0.000759	0.227133	0.002892
TK2|36723|RI	-2.9785	0.017497	0.001221	0.250705	0.002897
SCN1B|49017|AA	2.978051	6E+18	2628074	1.37E+31	0.002901
RFXANK|48606|AP	-2.9773	1.34E-07	3.99E-12	0.004478	0.002908
RFXANK|48607|AP	2.977295	7483878	223.2873	2.51E+11	0.002908
TMEM116|24566|ES	2.976419	127.2451	5.232876	3094.153	0.002916
PUS1|25231|AD	-2.9696	0.00344	8.14E-05	0.145358	0.002982

GALK1|43493|AT	2.969535	683.0633	9.197058	50730.95	0.002983
GALK1|43494|AT	-2.96953	0.001464	1.97E-05	0.108731	0.002983
USP15|22830|ES	2.9682	1625.313	12.32222	214380.3	0.002995
ZNF773|52278|AT	2.966175	69503.53	43.90828	1.1E+08	0.003015
ACAA2|45491|AP	-2.96616	8.36E-09	3.84E-14	0.001819	0.003015
ZNF773|52279|AT	-2.96604	1.44E-05	9.09E-09	0.022786	0.003017
ACLY|40961|ES	-2.96494	4.33E-20	6.85E-33	2.73E-07	0.003027
WFDC8|59591|AT	-2.9631	0.000201	7.19E-07	0.056011	0.003046
USP36|43912|AT	2.961239	157465.4	57.18859	4.34E+08	0.003064
MYO5C|30648|ES	-2.95903	2.78E-18	6.52E-30	1.18E-06	0.003086
TRA2A|78976|ES	2.957816	211.9163	6.09196	7371.765	0.003098
GPN3|24424|AP	-2.95659	1.10E-12	1.31E-20	9.32E-05	0.003111
SNX17|52998|ES	-2.95491	0.000691	5.54E-06	0.086274	0.003128
INO80C|45173|AT	-2.95184	1.73E-14	1.26E-23	2.38E-05	0.003159
INO80C|45172|AT	2.951637	5.76E+13	41996.03	7.91E+22	0.003161
GOLT1B|20705|ES	-2.9508	0.005867	0.000193	0.178099	0.003169
ADAMTSL1|85950|AT	2.949844	623.4832	8.666831	44852.77	0.003179
ZNF346|74698|AT	-2.94981	9.72E-08	2.13E-12	0.004435	0.00318
ZNF346|74699|AT	2.949117	10260030	224.6905	4.69E+11	0.003187
IRF7|13720|RI	2.948615	331.3492	6.998988	15686.88	0.003192
ZNF707|85473|AP	-2.9473	3.08E-05	3.08E-08	0.030814	0.003206
CACNB3|21469|AP	2.946074	42.46944	3.507115	514.284	0.003218
CABIN1|61386|AP	-2.94319	1.62E-05	1.05E-08	0.025118	0.003249
PHPT1|88225|ES	2.942615	1230930	108.0838	1.4E+10	0.003255
CD63|22242|AP	-2.94027	1.14E-17	5.82E-29	2.25E-06	0.003279
TARBP2|22086|ES	-2.93882	3.50E-18	7.98E-30	1.53E-06	0.003295
FAM86C1|17437|ES	2.937057	103.7284	4.684294	2296.95	0.003313
MTHFS|32161|AD	2.936624	36234.58	32.82988	39992377	0.003318
MKL2|34066|AP	-2.93559	0.000739	6.00E-06	0.091071	0.003329
ZNF7|85660|AA	-2.93351	0.000648	4.80E-06	0.087464	0.003352
PLEKHA5|20649|ES	2.928399	243.1828	6.152322	9612.288	0.003407
NFYC|2019|AA	2.92639	29177.56	29.82364	28545489	0.003429
IQCK|34339|ES	-2.92374	2.09E-11	1.44E-18	0.000301	0.003459
CCDC66|65341|AT	2.922051	795.4801	9.016409	70181.89	0.003477
SEC31A|69731|ES	-2.92064	0.00366	8.48E-05	0.157967	0.003493
KANK2|47641|AA	2.916102	513.5416	7.740152	34072.33	0.003544
IFT74|86038|AP	2.915018	5887	17.18457	2016737	0.003557
SECISBP2|86799|AA	-2.90922	0.000334	1.52E-06	0.073413	0.003623
MYPN|11921|AT	-2.90642	0.002078	3.23E-05	0.133818	0.003656
ABCB8|82312|ES	2.905291	128.866	4.859588	3417.256	0.003669
GLRX|72830|AA	2.902224	418126.5	66.84358	2.62E+09	0.003705
PRPSAP2|39673|ES	-2.90083	0.000348	1.60E-06	0.075523	0.003722
NEDD1|23836|ES	-2.89968	0.002012	3.03E-05	0.133723	0.003735
DTNA|45112|ES	-2.89917	6.97E-06	2.27E-09	0.021349	0.003742
EMC3|63298|AT	-2.8956	5.98E-23	5.42E-38	6.59E-08	0.003784
SYTL3|78285|ES	-2.89547	0.000528	3.20E-06	0.087331	0.003786
TTC9C|16416|ES	2.894232	271176.4	56.73783	1.3E+09	0.003801
RPS6KC1|9783|ES	-2.89402	0.013098	0.000695	0.246786	0.003803
EVL|29239|AP	2.893832	16.28663	2.460764	107.7935	0.003806
BOP1|85554|RI	2.893563	215.2773	5.658936	8189.584	0.003809
RARA|40857|AP	2.88461	766.478	8.406415	69885.74	0.003919
IDH3G|90494|RI	2.882418	1.52E+09	867.3089	2.65E+15	0.003946
PACSIN2|62559|ES	-2.88157	0.000269	1.01E-06	0.072165	0.003957
ACLY|40962|AA	2.879099	553.4423	7.5112	40778.88	0.003988
SEC24B|70325|AA	-2.87868	0.001133	1.12E-05	0.114797	0.003993
PCGF2|40588|ES	-2.87858	3.76E-10	1.44E-16	0.000983	0.003995
CDKN1A|75989|AD	2.878506	97488.62	39.08356	2.43E+08	0.003996
PAM|72901|ES	-2.87793	2.37E-09	3.17E-15	0.001774	0.004003

CBWD1|85691|ES	-2.87643	2.21E-16	4.75E-27	1.03E-05	0.004022
UBAC2|26180|ES	-2.87636	3.26E-10	1.12E-16	0.000949	0.004023
IWS1|55221|ES	-2.87574	5.44E-11	5.50E-18	0.000539	0.004031
CTNNAL1|87155|AA	-2.87395	8.37E-16	4.37E-26	1.60E-05	0.004054
ZNF74|61154|ES	-2.8732	0.005055	0.000137	0.186264	0.004063
AKT2|49868|AP	-2.86754	0.022596	0.001694	0.301334	0.004137
AKT2|49869|AP	2.867537	44.25599	3.318552	590.1949	0.004137
LRRC23|19994|AP	-2.86679	0.000769	5.72E-06	0.103519	0.004147
LRRC23|19997|AP	2.866763	1299.758	9.659626	174889.8	0.004147
ROGDI|33859|ES	-2.86422	1.42E-06	1.42E-10	0.014265	0.00418
SEC11A|32313|ES	2.858297	293.9297	5.966823	14479.18	0.004259
TTLL5|28518|AT	-2.85785	1.30E-07	2.48E-12	0.006871	0.004265
EIF4E|70009|AP	2.856943	193.7948	5.225702	7186.869	0.004277
EIF4E|70010|AP	-2.85621	0.005163	0.000139	0.191573	0.004287
BIN1|55202|ES	2.855643	5814.605	15.16178	2229924	0.004295
ZBTB45|52479|AD	2.854409	1263.605	9.373626	170339.4	0.004312
TOP3B|61268|AD	2.852906	871.1037	8.321927	91183.4	0.004332
HIST1H2BD|75606|AT	-2.85184	0.000389	1.77E-06	0.08583	0.004347
IFT74|86040|AP	-2.85147	0.000416	1.98E-06	0.087711	0.004352
DMKN|49144|ES	-2.84576	0.000112	2.13E-07	0.058915	0.004431
FAM185A|81134|ES	-2.84454	0.001915	2.57E-05	0.142833	0.004448
NOLC1|12939|AD	2.842289	7162.993	15.72996	3261831	0.004479
KLC1|29478|AD	-2.84197	3.98E-05	3.68E-08	0.043103	0.004484
IFIH1|55768|AT	-2.84184	1.11E-07	1.77E-12	0.006948	0.004485
IFIH1|55767|AT	2.841836	9005757	143.9165	5.64E+11	0.004485
LEF1|70290|AT	-2.84001	1.39E-17	3.25E-29	5.98E-06	0.004511
MIA3|9888|AP	-2.83919	0.004708	0.000116	0.190251	0.004523
MIA3|9887|AP	2.838702	212.5278	5.25371	8597.367	0.00453
BTBD11|24189|AP	-2.83779	0.022914	0.001688	0.310971	0.004543
EIF3E|84875|ES	-2.83379	0.005818	0.000166	0.204525	0.0046
TJP2|86531|AP	2.833494	486.1368	6.73431	35093.28	0.004604
IL1R1|54771|AP	-2.83325	0.010522	0.000451	0.245672	0.004608
ATP2A2|24418|RI	-2.8332	0.00015	3.41E-07	0.066324	0.004608
CIRBP|46427|AA	-2.83093	0.001015	8.59E-06	0.119953	0.004641
LIPT1|54676|ES	2.830848	80.95949	3.864213	1696.19	0.004642
NDRG2|26511|ES	2.829869	1227.235	8.903075	169166.8	0.004657
PAM|72902|ES	-2.8298	5.89E-05	6.93E-08	0.050097	0.004658
C11orf80|17121|AA	2.828712	10849.97	17.35212	6784291	0.004674
METTL21A|57191|AT	2.82543	486.4601	6.653916	35564.53	0.004722
TSR1|38388|AD	-2.82454	4.57E-18	4.25E-30	4.93E-06	0.004735
C19orf44|48172|AA	-2.82361	3.73E-10	1.07E-16	0.001307	0.004749
FAM86C1|17436|ES	2.822222	451.0436	6.470369	31441.84	0.004769
YAF2|21146|ES	2.818367	16.52414	2.349676	116.2063	0.004827
TMEM134|17233|AA	2.818307	8763.092	15.87725	4836591	0.004828
RPS6KC1|9781|RI	2.816097	383.6051	6.102916	24111.89	0.004861
PDLIM5|69971|AT	-2.81422	0.00034	1.31E-06	0.088577	0.00489
ELP4|14825|ES	2.813912	1076.147	8.319415	139203.7	0.004894
PFKFB2|9614|AP	2.812449	2750.051	11.02815	685770.3	0.004917
SP140|57870|AT	-2.80694	0.01664	0.000953	0.290565	0.005002
HOXB3|42198|AT	-2.80591	1.84E-05	9.02E-09	0.03733	0.005018
HOXB3|42197|AT	2.805885	54492.74	26.7865	1.11E+08	0.005018
USP4|64851|AT	2.805583	1.68E+10	1206.67	2.33E+17	0.005023
MKNK2|46567|AT	-2.80481	6.83E-20	2.77E-33	1.69E-06	0.005035
ZNF384|19927|AD	2.800156	28.29965	2.726526	293.7328	0.005108
CHEK2|61544|ES	2.800051	18.18593	2.387553	138.5218	0.005109
C12orf57|20020|AD	-2.79831	2.90E-13	4.79E-22	0.000175	0.005137
EMC9|26859|RI	2.797697	1428.151	8.803486	231682.8	0.005147
HNRNPH1|74913|AD	-2.7973	1.22E-06	8.74E-11	0.016965	0.005153

ANK3|11843|AP	2.795923	29.74136	2.75755	320.7734	0.005175
PCM1|82839|ES	-2.79243	8.99E-05	1.30E-07	0.062188	0.005231
TMUB1|82347|RI	-2.78972	0.003942	8.06E-05	0.192704	0.005275
C6orf1|75776|ES	-2.78799	3.20E-21	1.25E-35	8.19E-07	0.005304
MCRS1|21591|ES	2.785898	2240159	76.32471	6.57E+10	0.005338
IL32|33385|RI	2.784667	11896.8	16.10548	8787929	0.005358
KDM4C|85835|ES	-2.78153	1.28E-09	6.89E-16	0.00236	0.00541
GPR64|88618|ES	-2.77937	0.002929	4.79E-05	0.17912	0.005446
RBM38|59898|ES	-2.77876	9.37E-08	1.03E-12	0.008492	0.005457
ZNF438|11137|ES	2.777176	59.75817	3.332138	1071.696	0.005483
PKP4|55684|ES	-2.77674	2.85E-10	5.23E-17	0.001558	0.005491
PPIA|97441|ES	2.774926	436.2771	5.960275	31934.39	0.005521
SLC44A5|3480|AT	2.768846	13.1313	2.121773	81.26738	0.005626
SNX3|77149|AD	-2.7671	6.39E-21	3.18E-35	1.29E-06	0.005656
ZNF205|33452|AA	-2.7664	0.003642	6.82E-05	0.194586	0.005668
NKX2-1|102505|AA	2.765791	815.9791	7.052322	94411.73	0.005678
SEMA4B|32468|AP	2.764866	131.8082	4.141459	4194.993	0.005695
PIGG|68356|AT	-2.76344	6.93E-10	2.21E-16	0.002172	0.00572
PIGG|68354|AT	2.763434	1.44E+09	460.2813	4.52E+15	0.00572
ZNF329|52413|RI	-2.76268	7.58E-17	2.77E-28	2.07E-05	0.005733
HLTF|67208|RI	-2.7624	1.63E-12	7.05E-21	0.000377	0.005738
DPH6|29892|AT	-2.7614	2.55E-09	2.02E-15	0.003204	0.005755
DPH6|29893|AT	2.76139	3.93E+08	312.091	4.95E+14	0.005756
CPSF7|16201|ES	-2.75895	0.007371	0.000225	0.241231	0.005799
CD320|47212|ES	2.758078	894.8817	7.147624	112039.1	0.005814
ZSCAN30|45156|AA	2.75326	148.723	4.225971	5233.954	0.005901
SLC44A5|3481|AT	-2.75257	0.076753	0.012337	0.477488	0.005913
PRICKLE4|76140|AA	2.750151	132.4201	4.070901	4307.423	0.005957
ZNF263|33514|ES	-2.74906	0.013397	0.000619	0.289984	0.005977
NOSTRIN|55840|ES	-2.74539	3.77E-06	5.06E-10	0.028072	0.006044
STAG2|90031|AP	2.740489	10179344	99.05695	1.05E+12	0.006135
CCDC92|25124|ES	-2.73923	1.99E-07	3.19E-12	0.012406	0.006158
RHOC|4236|ES	2.73833	29.47931	2.616424	332.1441	0.006175
DUSP4|83257|AP	-2.73697	2.88E-08	1.14E-13	0.00723	0.006201
CCDC120|89057|AP	2.733465	78.76652	3.44046	1803.295	0.006267
SMIM12|1702|AD	2.732487	10193753	95.80368	1.08E+12	0.006286
EIF5A|38907|AP	-2.7284	2.00E-06	1.61E-10	0.024829	0.006364
CKMT2|72660|ES	2.728335	11.1764	1.973465	63.29574	0.006365
TM9SF1|26891|AP	-2.72775	0.001677	1.70E-05	0.165504	0.006377
TM9SF1|26892|AP	2.727751	596.1623	6.042112	58822.07	0.006377
LYRM1|34421|ES	-2.72757	0.014084	0.000658	0.301303	0.00638
C2orf81|54053|RI	-2.72756	8.09E-06	1.78E-09	0.0369	0.00638
RRM1|14037|ES	-2.72671	3.96E-07	9.91E-12	0.01584	0.006397
FBXL12|47421|ES	2.724614	96.41181	3.604432	2578.836	0.006438
TSC22D4|80951|AA	2.72425	2.57E+15	21055.98	3.14E+26	0.006445
DALRD3|64806|AP	-2.72289	0.000123	1.89E-07	0.080287	0.006471
LAT2|80066|ES	-2.71946	3.62E-05	2.27E-08	0.057477	0.006539
CALCRL|56490|ES	-2.71753	0.00186	2.00E-05	0.173315	0.006577
ZNF816|51644|AT	2.716173	408675.2	36.49739	4.58E+09	0.006604
EIF4A2|96628|ES	2.714255	100.0185	3.596021	2781.88	0.006642
GTPBP10|80390|ES	-2.71403	5.43E-07	1.63E-11	0.018171	0.006647
PARP9|66442|AT	-2.71371	5.21E-15	2.52E-25	0.000108	0.006653
COPS7A|19945|ES	2.712145	89.35904	3.476363	2296.952	0.006685
FXR1|67746|ES	-2.70908	1.89E-06	1.37E-10	0.026146	0.006747
FBXO7|61931|AP	-2.70813	0.001382	1.18E-05	0.162174	0.006766
FBXO7|61930|AP	2.708127	723.8096	6.166187	84963.42	0.006766
MKL2|34070|AT	-2.70744	1.39E-06	7.96E-11	0.024136	0.006781
MED8|2172|AA	2.706574	201.3058	4.32036	9379.778	0.006798

TMEM182|54811|AP	-2.70487	0.000914	5.74E-06	0.145567	0.006833
FLAD1|7863|AT	-2.70438	2.51E-13	1.85E-22	0.00034	0.006843
HNRNPA2B1|79039|ES	2.702662	5.66E+08	254.2268	1.26E+15	0.006879
TIMM10B|14145|AT	-2.70212	4.10E-17	5.32E-29	3.16E-05	0.00689
FOXK2|44408|ES	2.702018	911899.2	43.32884	1.92E+10	0.006892
UBE2W|84194|ES	-2.69985	1.65E-09	6.96E-16	0.00392	0.006937
ADCK5|85593|RI	2.699178	366.206	5.036378	26627.63	0.006951
PTS|18758|AP	-2.69771	5.61E-11	2.00E-18	0.001572	0.006982
BACH1|60322|AP	2.697568	142.4539	3.88048	5229.534	0.006985
UBBP4|39829|RI	-2.69324	0.000148	2.43E-07	0.090693	0.007076
CLP1|15883|AP	-2.69157	0.001372	1.13E-05	0.166677	0.007112
SLC39A1|7765|AP	2.691075	416310	33.62659	5.15E+09	0.007122
TRMT10B|86427|AA	-2.69019	0.000902	5.46E-06	0.149116	0.007141
COMMD5|85672|AP	2.690101	1516.394	7.300011	314992.8	0.007143
COMMD5|85671|AP	-2.6901	0.000659	3.17E-06	0.136986	0.007143
TTLL3|63203|AP	2.689276	12481.53	12.90857	12068623	0.007161
TTLL3|63199|AP	-2.68921	8.01E-05	8.29E-08	0.077484	0.007162
FBXL12|47431|RI	2.688735	236.6263	4.400281	12724.64	0.007172
CXorf40A|90313|AD	-2.68617	0.005144	0.00011	0.240572	0.007228
ZNF395|83211|AP	2.683564	1491.771	7.174004	310200.5	0.007284
SLC35F5|55070|ES	-2.6825	2.84E-05	1.35E-08	0.059623	0.007307
MTFR1L|1216|AD	2.680844	42.39173	2.738901	656.1241	0.007344
CYP3A5|80708|AT	2.676141	16.94441	2.132586	134.6314	0.007448
ADAMTSL1|85946|AT	-2.67576	0.005719	0.00013	0.25122	0.007456
PPFIBP1|20888|AT	-2.67573	3.53E-14	4.94E-24	0.000252	0.007457
RGS6|28206|AT	2.673936	150.5057	3.814449	5938.462	0.007497
TMA16|71020|ES	-2.67353	2.12E-11	3.17E-19	0.001416	0.007506
NDUFA7|47217|AT	-2.6724	0.001393	1.12E-05	0.173225	0.007531
PPIE|1904|AD	2.671734	2062.292	7.63779	556843	0.007546
TCF3|46537|RI	-2.6713	7.27E-08	4.21E-13	0.012563	0.007556
WDR55|73716|RI	-2.66715	6.83E-06	1.09E-09	0.042689	0.00765
CRTAC1|12748|AT	-2.66492	8.25E-07	2.77E-11	0.02459	0.007701
SFTA3|27259|ES	2.664625	18235494	83.20545	4E+12	0.007707
RPLP0|24727|AA	-2.66297	0.023586	0.001496	0.37187	0.007745
TLE3|31425|ES	-2.66255	0.002903	3.94E-05	0.214035	0.007755
WDR90|32931|AA	-2.66234	0.000152	2.36E-07	0.098417	0.00776
MTUS1|82815|AP	2.659789	36.29668	2.57289	512.0503	0.007819
TSPAN4|13794|AP	2.65891	434039.1	30.33423	6.21E+09	0.007839
TPM3|7789|AT	-2.65675	1.62E-09	5.27E-16	0.004945	0.00789
BAIAP2|44102|ES	-2.65487	1.29E-07	1.06E-12	0.015723	0.007934
FBXL20|40641|AT	-2.65386	4.97E-05	3.30E-08	0.07496	0.007958
FBXL20|40642|AT	2.653563	20083.39	13.32579	30267821	0.007965
ZNF772|52255|AT	2.651782	528955.7	31.12947	8.99E+09	0.008007
ZNF772|52253|AT	-2.65178	1.89E-06	1.11E-10	0.032124	0.008007
NWD1|48207|AD	-2.6511	0.025094	0.001646	0.382623	0.008023
GGT1|100018|ES	2.649631	15054.84	12.22924	18533303	0.008058
PRICKLE4|76139|AA	-2.64931	1.22E-07	9.32E-13	0.015877	0.008066
ERLIN2|83343|AP	2.649239	16798.59	12.56969	22450250	0.008067
RNH1|13672|AA	-2.64748	0.006822	0.00017	0.273836	0.008109
DZIP3|66036|ES	-2.64719	2.68E-07	3.65E-12	0.019674	0.008116
GOLGA4|63986|ES	-2.6452	3.09E-10	2.77E-17	0.003438	0.008164
INADL|3239|AT	-2.6438	5.31E-05	3.60E-08	0.07841	0.008198
GGT2|121589|ES	2.64199	126.5183	3.488734	4588.166	0.008242
TG|319464|ES	2.641864	105.4949	3.328303	3343.798	0.008245
MAGI1|65529|AT	-2.64157	1.49E-23	1.72E-40	1.29E-06	0.008252
ZNF20|47779|AA	-2.64074	0.008668	0.000256	0.294038	0.008273
XBP1|61563|RI	-2.64043	0.009548	0.000302	0.301584	0.00828
FANCD2|63305|AT	2.638685	70130804	104.2606	4.72E+13	0.008323

ZNF181|48997|AP	2.636876	71.31195	2.990371	1700.59	0.008367
ZNF181|48996|AP	-2.63685	0.013613	0.000558	0.331877	0.008368
TRIM11|10208|AT	-2.63684	6.84E-18	1.19E-30	3.92E-05	0.008368
ARL6IP4|25034|AD	2.636379	30598.23	14.15449	66145194	0.00838
PTCD2|72456|AT	-2.63596	0.00019	3.26E-07	0.111166	0.00839
PTCD2|72458|AT	2.635933	5249.105	8.994833	3063215	0.008391
PCNA|58649|AP	-2.63529	0.000176	2.83E-07	0.109067	0.008407
PCNA|58648|AP	2.635273	5690.518	9.168248	3531972	0.008407
FBLIM1|775|AA	2.634954	97.71861	3.234205	2952.48	0.008415
MAP9|70916|AA	-2.63441	0.000393	1.15E-06	0.134346	0.008428
PTK2B|83155|AT	-2.63074	8.66E-13	8.92E-22	0.00084	0.00852
LRP2BP|71336|AP	2.629944	218.708	3.945318	12124.03	0.00854
FAM185A|81137|AD	-2.62887	0.037265	0.003207	0.432985	0.008567
LRP2BP|71337|AP	-2.62837	0.004581	8.25E-05	0.254191	0.008579
PRSS16|75702|ES	-2.62814	0.002293	2.46E-05	0.213254	0.008585
SNX15|16730|ES	-2.62704	1.17E-18	4.88E-32	2.80E-05	0.008613
ACOT9|88692|AT	-2.62554	2.87E-08	6.72E-14	0.012247	0.008651
MFF|57806|ES	-2.62534	0.003075	4.10E-05	0.230835	0.008656
DTX2|80174|ES	-2.62499	0.000502	1.73E-06	0.145962	0.008665
PCNXL4|27766|AT	2.623179	11775516	61.34042	2.26E+12	0.008711
EFCAB6|62586|AT	-2.62183	0.045416	0.004502	0.458162	0.008746
NMRAL1|33737|AD	-2.62004	0.022343	0.001301	0.38379	0.008792
FAM86B1|82688|ES	2.619488	39.71578	2.526855	624.2318	0.008806
ICA1|78786|AT	-2.61825	1.01E-09	1.86E-16	0.005474	0.008838
CLCN3|71153|ES	-2.61722	0.003952	6.27E-05	0.249176	0.008865
FAM86B1|82686|ES	2.616739	27.86325	2.305092	336.8024	0.008877
TSTD1|8524|AP	-2.61369	9.20E-14	1.54E-23	0.000549	0.008957
LOXL3|54112|ES	-2.61265	0.007473	0.00019	0.294279	0.008984
PCNXL4|27767|AT	-2.6126	6.91E-08	2.93E-13	0.016265	0.008986
FAM49B|85146|ES	-2.61158	0.000314	7.40E-07	0.133691	0.009012
MAGED2|89250|RI	2.60942	574755.8	27.13136	1.22E+10	0.00907
ZNF720|36292|AD	2.609213	51.0307	2.660472	978.8236	0.009075
FGFR1|83430|ES	-2.60907	0.045668	0.004495	0.464009	0.009079
RAB17|58121|ES	-2.60904	1.01E-11	5.54E-20	0.001838	0.00908
FAM193A|68579|ES	2.608617	2827.559	7.21474	1108160	0.009091
EFCAB6|62585|AT	2.607174	21.84153	2.150138	221.8706	0.009129
FKBP11|21497|AA	-2.60682	0.000109	1.14E-07	0.103869	0.009139
LIN9|10012|AT	-2.60545	9.18E-10	1.46E-16	0.005769	0.009175
ARMC6|48572|ES	2.605411	3833.85	7.722985	1903202	0.009176
SYNC|1630|ES	2.604591	28.93057	2.29975	363.943	0.009198
PRMT5|26673|AD	-2.60359	5.22E-06	5.51E-10	0.049449	0.009225
ELMOD3|54217|RI	-2.60331	1.41E-05	3.16E-09	0.06333	0.009233
ABLIM2|68748|AA	-2.6026	0.002525	2.79E-05	0.228323	0.009252
DDX11|20968|ES	2.598777	510.9495	4.631851	56363.93	0.009356
CXorf23|88651|ES	2.598322	44.39919	2.539385	776.2858	0.009368
TSPAN14|12373|ES	-2.59785	1.85E-07	1.54E-12	0.022222	0.009381
ASPSCR1|44251|AP	2.594437	1778.859	6.234852	507524.2	0.009475
ASPSCR1|44250|AP	-2.59437	0.000562	1.97E-06	0.160415	0.009476
CUL4A|26367|AP	-2.59412	0.008435	0.000229	0.311179	0.009483
FAM219B|31801|AD	2.591775	207.8961	3.67311	11766.81	0.009548
CDCP1|64397|AT	-2.59175	7.41E-08	3.00E-13	0.018277	0.009549
SFTA3|121940|ES	2.590395	153.3066	3.403342	6905.836	0.009587
TANGO2|61112|AP	2.590208	1809.48	6.203282	527820.5	0.009592
HNRNPH1|74914|AD	-2.58852	1.77E-06	7.78E-11	0.040092	0.009639
DCLRE1C|10837|AT	-2.5883	1.55E-07	1.09E-12	0.02224	0.009645
DCLRE1C|10838|AT	2.588289	6436830	44.96265	9.21E+11	0.009645
SIAH1|36338|AP	2.587594	16233.75	10.5015	25094950	0.009665
TCEAL3|89760|AD	2.585421	2190842	34.19012	1.4E+11	0.009726

KLC1|29487|AD	-2.58433	0.000302	6.46E-07	0.141094	0.009757
PHKA1|89509|ES	-2.58393	1.78E-07	1.35E-12	0.023456	0.009768
TXN2|62049|AP	2.583583	32595.87	12.28503	86486616	0.009778
TXN2|62048|AP	-2.58355	3.07E-05	1.16E-08	0.081411	0.009779
CYP3A5|80710|AT	-2.58228	0.062106	0.007536	0.511861	0.009815
CRCP|79875|ES	-2.58211	0.004698	8.03E-05	0.274825	0.00982
DACT3|50576|AT	-2.58147	0.004606	7.75E-05	0.273801	0.009838
DACT3|50577|AT	2.58147	217.0861	3.652241	12903.42	0.009838
APOD|68181|ES	-2.58041	0.001019	5.44E-06	0.190803	0.009868
PLIN5|46809|AT	2.580184	8137.836	8.709653	7603560	0.009875
PLIN5|46808|AT	-2.58018	0.000123	1.32E-07	0.114817	0.009875
GLRX|72826|AT	-2.58013	2.83E-22	1.21E-38	6.62E-06	0.009876
MANBAL|59340|ES	2.580056	958.4239	5.207014	176411.4	0.009878
MUTYH|2652|ES	-2.58003	2.63E-08	4.58E-14	0.015072	0.009879
AP4B1|4309|RI	2.579895	45.36057	2.500856	822.751	0.009883
MTA3|53367|AT	-2.57983	0.000539	1.77E-06	0.163934	0.009885
MTA3|53366|AT	2.579766	1855.306	6.098933	564387.2	0.009887
TPM1|30985|AT	-2.57871	9.94E-06	1.57E-09	0.063051	0.009917
FAM228B|52815|ES	-2.57819	0.005117	9.28E-05	0.282249	0.009932
SIX5|95264|AA	2.575477	104.6748	3.038938	3605.476	0.01001
CCPG1|30723|AP	2.574938	208.2987	3.579099	12122.7	0.010026
FASTK|82341|ES	2.574739	3.48E+22	241122.4	5.02E+39	0.010032
ERLIN2|83344|AP	-2.57215	0.000484	1.44E-06	0.16257	0.010107
PPP1R1A|22198|ES	-2.57178	0.002749	3.07E-05	0.245925	0.010118
RPS15|46485|AA	-2.57051	0.011795	0.000399	0.348328	0.010155
PEX26|61024|RI	-2.57005	0.001372	9.00E-06	0.209142	0.010168
SPTBN4|49913|AT	-2.56847	0.002429	2.46E-05	0.240202	0.010215
RAD23B|87146|AP	2.56752	898.6952	4.999394	161550.2	0.010243
MSANTD1|68631|AT	2.567408	3064.145	6.681205	1405283	0.010246
C11orf74|15439|ES	-2.56736	2.51E-07	2.30E-12	0.027454	0.010248
ARHGEF26|67329|AP	-2.56734	0.034399	0.002626	0.450588	0.010248
RAD23B|87147|AP	-2.56727	0.001114	6.20E-06	0.200162	0.01025
HIST4H4|20554|AT	-2.5669	0.006228	0.000129	0.300938	0.010261
HIST4H4|20553|AT	2.566898	160.5716	3.322943	7759.163	0.010261
INSIG2|55092|ES	-2.56408	9.73E-08	4.25E-13	0.022285	0.010345
P4HA2|73261|ES	-2.56276	4.30E-07	5.81E-12	0.031806	0.010384
TMEM126B|18123|ES	2.561943	93047.63	14.706	5.89E+08	0.010409
KIF1B|602|AT	-2.56064	2.79E-05	9.10E-09	0.085425	0.010448
KIF1B|601|AT	2.560636	35858.41	11.70611	1.1E+08	0.010448
PHLDB2|66063|AT	-2.56008	2.80E-07	2.69E-12	0.029101	0.010465
ZFYVE26|28102|AT	-2.55637	4.91E-09	2.09E-15	0.011522	0.010577
FAM71D|28041|AT	2.553772	16.59092	1.921528	143.2499	0.010656
DDX11|20980|ES	-2.55353	0.000104	9.17E-08	0.118712	0.010664
AVL9|79198|ES	-2.55238	0.000103	8.95E-08	0.118756	0.010699
CROT|80321|AP	-2.5521	2.38E-05	6.69E-09	0.084566	0.010708
NKX2-1|102506|ES	2.551043	393.3259	3.992144	38752.43	0.01074
FAM13A|69911|ES	-2.55068	0.001716	1.29E-05	0.22885	0.010751
ABCB8|82291|AD	2.550002	2255.491	5.968939	852285.4	0.010772
PDCD6|71426|AD	-2.54913	7.26E-05	4.76E-08	0.110485	0.010799
CCNDBP1|30222|ES	-2.5483	2.08E-12	2.16E-21	0.00201	0.010825
PANK1|12494|AP	2.548152	53.78798	2.508954	1153.129	0.01083
ZNF562|47394|ES	-2.54711	0.000139	1.50E-07	0.12916	0.010862
PRDM1|77110|AP	2.546224	43.36692	2.382047	789.5266	0.01089
TST|62070|RI	2.545303	846665.5	23.07732	3.11E+10	0.010918
SPATA13|25474|AP	2.545089	83.39521	2.764886	2515.388	0.010925
CACNB3|21470|AP	-2.54447	0.002338	2.20E-05	0.248638	0.010944
ZMIZ2|79561|AA	-2.54434	0.000163	1.96E-07	0.134855	0.010949
CYB5RL|3129|AT	2.544322	58.47164	2.545757	1342.993	0.010949

CYB5RL|3128|AT	-2.5443	0.017103	0.000745	0.392828	0.01095
CASP8|56825|ES	-2.54354	0.008045	0.000196	0.330716	0.010974
GGT1|61437|AA	-2.54351	0.027077	0.001678	0.43692	0.010975
FAM49B|85136|AP	2.543102	138.4348	3.097359	6187.271	0.010987
CMTM7|63816|ES	-2.54296	1.81E-07	1.15E-12	0.028471	0.010992
FADS1|16298|AP	-2.54171	0.007911	0.000189	0.330328	0.011031
PNPLA6|47115|ES	2.541288	227.8866	3.462044	15000.48	0.011044
SSSCA1|16871|RI	2.541119	5.59E+09	169.5282	1.84E+17	0.01105
IFNGR1|77930|AP	-2.53975	1.24E-06	3.42E-11	0.044819	0.011093
KRIT1|80426|ES	-2.53975	7.54E-11	1.16E-18	0.004888	0.011093
OSMR|71851|AD	2.538343	92.37298	2.804551	3042.472	0.011138
GPR110|76434|AP	-2.53793	7.03E-05	4.36E-08	0.113284	0.011151
TASP1|58706|ES	-2.53516	3.65E-14	1.49E-24	0.000894	0.01124
SNX4|66556|ES	-2.53479	1.52E-08	1.37E-14	0.01687	0.011251
EVA1C|60349|ES	2.533503	1.26E+17	7440.098	2.15E+30	0.011293
APLP2|19479|ES	2.532421	809.9121	4.544189	144350.9	0.011328
SPATA6L|85760|AD	2.532383	1068.866	4.837901	236151.1	0.011329
C18orf21|45196|RI	2.532379	862.4421	4.608798	161388.4	0.011329
BCAS3|42871|ES	-2.53088	0.005885	0.00011	0.313976	0.011378
RANBP1|61139|AA	2.529161	1.71E+08	71.23332	4.09E+14	0.011434
ITGB1BP1|52625|ES	-2.52915	0.00047	1.24E-06	0.178301	0.011434
AHNAK|16349|ES	-2.5276	8.12E-05	5.47E-08	0.120612	0.011485
CDK2AP1|25064|AP	-2.52598	0.001078	5.37E-06	0.216314	0.011538
PANK1|12493|AP	-2.52491	0.019024	0.000878	0.412089	0.011573
ST7L|4209|ES	-2.52397	0.020948	0.001041	0.421543	0.011604
SACM1L|64410|ES	2.522877	378540.1	17.56333	8.16E+09	0.01164
CDK2AP1|25065|AP	2.522025	37772.81	10.4731	1.36E+08	0.011668
C19orf66|47453|ES	2.521158	17753867	41.08167	7.67E+12	0.011697
C16orf93|94142|ES	2.520322	150.0667	3.046953	7390.992	0.011725
CD9|19819|AP	2.520279	1678.254	5.211457	540450.7	0.011726
CD9|19817|AP	-2.52028	0.000596	1.85E-06	0.191886	0.011726
IMPDH1|81676|ES	-2.51984	4.41E-10	2.33E-17	0.008343	0.011741
NR4A2|55614|AP	2.518148	12.15601	1.739641	84.942	0.011797
CREBZF|18139|RI	2.517195	307.3721	3.553809	26584.88	0.011829
ASMTL|88387|AP	-2.517	3.00E-10	1.15E-17	0.007809	0.011836
FAM122C|90162|AT	-2.51563	0.009086	0.000233	0.354022	0.011882
FAM122C|90163|AT	2.515608	110.0589	2.824564	4288.435	0.011883
INTU|70537|AA	-2.51502	0.038568	0.003051	0.487513	0.011903
EFEMP2|16933|ES	2.513239	5.19E+32	15922689	1.69E+58	0.011963
DCTD|96838|ES	2.512588	311.2793	3.53467	27412.68	0.011985
MS4A6A|16065|AD	2.512268	3.46E+14	1571.85	7.63E+25	0.011996
MAP3K2|55209|AP	-2.50751	0.001787	1.27E-05	0.251157	0.012159
ZNF720|36288|AA	2.506877	197.2611	3.167362	12285.29	0.01218
COL1A1|196922|ES	-2.50591	9.88E-18	4.95E-31	0.000197	0.012214
PUS1|25229|AD	-2.5053	0.003451	4.09E-05	0.291125	0.012235
ETFA|31944|ES	-2.50461	0.047791	0.004425	0.516197	0.012259
ZMIZ1|12300|ES	2.503918	3485.084	5.881851	2064964	0.012283
NUPL2|78961|AT	-2.50326	2.57E-06	1.08E-10	0.061198	0.012305
NUPL2|78960|AT	2.503254	389167.6	16.33983	9.27E+09	0.012306
CTNND1|15932|RI	-2.50303	2.21E-10	6.06E-18	0.008034	0.012314
TG|319468|ES	2.502966	73.28273	2.538632	2115.453	0.012316
GNAL|44641|AP	2.502194	7.079003	1.528236	32.79092	0.012343
AGAP3|82352|AT	2.50149	3818025	26.59773	5.48E+11	0.012367
AGAP3|82351|AT	-2.50148	2.62E-07	1.82E-12	0.0376	0.012368
NRG1|83314|AT	-2.50095	1.06E-07	3.60E-13	0.030964	0.012386
EIF4G1|67875|AP	2.500681	471.5383	3.785204	58741.44	0.012395
METTL21A|57189|AT	-2.50034	0.001357	7.67E-06	0.240049	0.012407
HAS3|37253|AT	2.500278	44916.69	10.12495	1.99E+08	0.01241

HAS3|37254|AT	-2.50027	2.23E-05	5.02E-09	0.098769	0.01241
CCAR2|83036|AP	-2.49997	0.000127	1.12E-07	0.143973	0.01242
CCAR2|83035|AP	2.499967	7884.199	6.945693	8949515	0.01242
SFTA3|121939|ES	2.498913	219.389	3.198434	15048.46	0.012458
COPA|8458|AA	2.498771	2116704	23.12031	1.94E+11	0.012462
USP3|31052|ES	-2.49853	0.005559	9.46E-05	0.326531	0.012471
TNFRSF10C|83066|ES	-2.49816	2.37E-16	1.30E-28	0.00043	0.012484
ZSCAN9|75724|AD	2.497793	214.2859	3.176242	14456.85	0.012497
PTPRE|13458|AP	-2.49759	0.090663	0.013781	0.596456	0.012504
ZDHHC4|78748|AD	2.496758	16.99973	1.838825	157.1606	0.012533
AASDHPPT|18561|ES	-2.49558	3.28E-15	1.38E-26	0.000779	0.012575
ASPSCR1|44258|ES	2.494044	3.92E+08	69.20369	2.22E+15	0.01263
PSEN2|10024|AP	-2.49403	0.056673	0.005939	0.540818	0.01263
ARHGEF26|67328|AP	2.492363	26.20007	2.008938	341.695	0.01269
NDUFAF6|84599|AD	2.492047	205.4071	3.117253	13535.02	0.012701
PSEN2|10023|AP	2.491749	17.38355	1.839367	164.289	0.012712
PRPSAP2|39671|ES	-2.49113	2.43E-05	5.69E-09	0.103784	0.012734
DMPK|50526|AA	-2.48955	0.006257	0.000115	0.339816	0.012791
EVL|29241|AP	-2.48711	0.085118	0.012213	0.59322	0.012878
ACAD8|19554|ES	-2.48635	1.14E-05	1.45E-09	0.089855	0.012906
PADI4|852|AT	-2.48454	0.020425	0.000949	0.439755	0.012972
SULT1A1|235342|ES	2.483189	83.7666	2.542169	2760.179	0.013021
PADI4|851|AT	2.48247	48.88132	2.267398	1053.8	0.013048
EPS8L1|52013|RI	2.480871	33672.15	8.924596	1.27E+08	0.013106
RPS6|214602|AA	2.480844	96251.37	11.12552	8.33E+08	0.013107
ZMYND8|59712|ES	-2.48028	0.000904	3.55E-06	0.229846	0.013128
TNRC6B|62335|AP	2.479694	54.83909	2.314747	1299.203	0.01315
TMEFF1|87084|AP	2.478424	347.0312	3.399523	35425.74	0.013196
PTGR2|28317|AD	2.477762	16.28173	1.791508	147.9729	0.013221
PRR13|22026|AP	-2.47301	5.00E-10	2.13E-17	0.011762	0.013398
RGS11|32860|AT	2.472968	368.4161	3.40701	39838.58	0.0134
RHBDF1|32789|AT	-2.46981	9.82E-11	1.12E-18	0.008591	0.013519
CPNE1|59205|AD	2.468657	1613.578	4.581411	568304	0.013562
DDT|61355|AP	2.468572	480.6575	3.569011	64732.69	0.013565
BAIAP2|44097|RI	2.468418	26.99405	1.971597	369.588	0.013571
LGALS3|27617|AT	-2.4678	0.00041	8.36E-07	0.200877	0.013594
PCDHA2|73770|AT	-2.46584	0.03883	0.002936	0.513524	0.013669
NRSN2|58447|ES	2.462648	150.741	2.783735	8162.723	0.013792
ARMCX3|89663|AA	-2.4626	0.005033	7.46E-05	0.339561	0.013793
CKLF|36732|ES	-2.46246	3.63E-10	1.11E-17	0.01185	0.013799
RARRES2|82226|RI	2.461791	12.74963	1.680164	96.74841	0.013825
APH1A|7444|RI	2.460392	35127.82	8.40537	1.47E+08	0.013879
TMEM151B|97224|AT	2.458844	13.25329	1.689315	103.9769	0.013939
TMEM79|8218|AP	2.458668	6.202981	1.447995	26.57259	0.013945
MVP|233980|ES	-2.45802	0.003207	3.29E-05	0.31238	0.013971
RPL7|84149|AD	-2.45781	3.18E-15	8.75E-27	0.001157	0.013979
TJP2|86538|ES	-2.45778	0.02856	0.001676	0.486654	0.01398
TMEM232|72938|ES	2.457415	22.46893	1.877582	268.8845	0.013994
PCK2|26813|AP	-2.45686	2.85E-13	2.79E-23	0.002902	0.014016
FAM76A|1342|ES	-2.45354	0.009071	0.000212	0.388277	0.014146
COL16A1|1493|ES	2.4529	43.48202	2.134202	885.8985	0.014171
SPARCL1|69872|ES	2.452866	30365590	31.88448	2.89E+13	0.014172
GAS7|39265|ES	-2.45284	5.03E-06	2.93E-10	0.086157	0.014173
PAM|72899|ES	-2.45282	8.44E-06	7.45E-10	0.095619	0.014174
SIPA1L1|28199|ES	2.451278	536.546	3.524524	81679.59	0.014235
TMEM151B|97223|AT	-2.45044	0.073913	0.009202	0.593697	0.014268
RAD51|30018|ES	-2.45006	0.00179	1.14E-05	0.282134	0.014283
FNTA|83748|AP	-2.44906	0.002294	1.77E-05	0.297089	0.014323

FNTA|83749|AP	2.449065	435.9147	3.36599	56453.4	0.014323
RCAN2|76415|AP	2.448408	7.953279	1.512356	41.82525	0.014349
ZNF562|47387|ES	-2.4484	0.000289	4.26E-07	0.196841	0.014349
ZNF720|94157|ES	-2.44838	0.003027	2.91E-05	0.314411	0.01435
NT5C3A|79208|AP	2.448042	860.5799	3.847011	192512.5	0.014363
TMEM108|66827|AT	-2.44597	0.000673	1.94E-06	0.234315	0.014446
TMEM108|66828|AT	2.445966	1484.807	4.26772	516587.7	0.014446
POM121C|80119|AP	-2.4446	0.001128	4.89E-06	0.260403	0.014501
POM121C|80118|AP	2.444327	885.6208	3.837257	204397.1	0.014512
RAMP2|41122|ES	-2.44419	8.01E-05	4.16E-08	0.154341	0.014518
UEVLD|14672|ES	-2.44366	5.23E-06	3.04E-10	0.090075	0.014539
ARRB2|38574|ES	-2.442	1.79E-06	4.37E-11	0.073376	0.014606
PUM2|52777|ES	-2.44153	0.000202	2.19E-07	0.186833	0.014625
PPP6R2|62824|AA	2.440703	2.76E+08	45.98394	1.66E+15	0.014659
SCML4|77135|AT	2.439531	38.25856	2.047084	715.0256	0.014706
TMEFF1|87085|AP	-2.43939	0.003049	2.90E-05	0.320289	0.014712
NT5C3A|79207|AP	-2.43903	0.001172	5.17E-06	0.265651	0.014727
C1orf63|1146|AA	-2.43858	0.073583	0.009036	0.599215	0.014745
CCPG1|30722|AP	-2.43857	0.005887	9.49E-05	0.365014	0.014746
PTBP1|46321|ES	-2.43789	1.81E-08	1.08E-14	0.030363	0.014773
TCF3|46538|AA	-2.4375	0.002423	1.91E-05	0.307303	0.014789
ALDH1A3|32741|AT	-2.43712	8.70E-20	4.08E-35	0.000185	0.014805
CCDC148|55661|AT	2.43579	24.01217	1.860647	309.8838	0.014859
TMEM79|8217|AP	-2.43406	0.163525	0.038049	0.702789	0.014931
IAH1|52628|AP	2.432705	2.46E+09	66.84539	9.09E+16	0.014986
IAH1|52629|AP	-2.43245	4.07E-10	1.11E-17	0.014999	0.014997
WBP2NL|62480|AT	-2.43198	0.05902	0.006033	0.577387	0.015017
SERF1A|72415|AT	-2.43139	8.18E-05	4.15E-08	0.161254	0.015041
PTBP2|3832|ES	2.430373	13.94393	1.665331	116.7535	0.015083
CXorf40A|90314|AD	-2.42997	0.007515	0.000145	0.3883	0.0151
RGN|88903|ES	2.429054	787.6727	3.625194	171143.5	0.015138
METTL21A|57190|AT	-2.42869	8.94E-12	1.08E-20	0.007373	0.015153
SLC36A1|74171|AT	-2.42855	0.000119	8.14E-08	0.174985	0.015159
ABCC9|20715|AT	-2.42807	3.86E-11	1.51E-19	0.009825	0.015179
SMAGP|21836|RI	-2.42793	0.020035	0.000853	0.470635	0.015185
CCDC130|47933|AP	-2.42735	0.001373	6.71E-06	0.281114	0.01521
CCDC130|47932|AP	2.42735	728.1226	3.557272	149036.3	0.01521
ZNF415|51676|ES	2.426816	16.54497	1.715684	159.5492	0.015232
SUMF2|79803|ES	-2.42627	0.002484	1.95E-05	0.315761	0.015255
UBALD1|33778|AD	-2.42603	2.83E-10	5.47E-18	0.014646	0.015265
RBM6|64939|ES	2.425949	92.39618	2.385446	3578.808	0.015268
KCNJ11|14528|AP	-2.42577	8.61E-08	1.69E-13	0.043994	0.015276
MUTYH|2650|ES	-2.42508	0.006334	0.000106	0.378768	0.015305
CAV1|81536|AD	2.424137	2499.972	4.473376	1397124	0.015345
CNRIP1|53837|AT	-2.42402	1.28E-08	5.30E-15	0.030821	0.01535
SPTAN1|87770|ES	-2.42347	9.44E-09	3.06E-15	0.029188	0.015373
ARHGEF26|67332|AA	-2.42329	0.012668	0.00037	0.433753	0.015381
NDRG2|26503|RI	-2.42161	0.0703	0.008198	0.60282	0.015452
CPNE1|59196|ES	-2.42154	1.07E-06	1.57E-11	0.072764	0.015455
NEDD1|23841|ES	-2.42133	0.010333	0.000255	0.418432	0.015464
AXL|50027|ES	2.42125	599.8727	3.3827	106378.7	0.015467
PIGV|1299|AP	-2.41891	0.01337	0.000405	0.441032	0.015567
CCL2|40220|RI	2.418488	11800.93	5.915602	23541471	0.015585
CYP4B1|2838|AA	2.418322	14335.36	6.134614	33498839	0.015592
ETFA|31939|ES	-2.41796	0.057613	0.005699	0.582405	0.015608
SCRN2|42120|RI	2.416612	245.0231	2.827933	21229.75	0.015666
CDK2AP2|17244|AD	2.416547	3784031	17.49202	8.19E+11	0.015669
ATG16L2|17663|ES	-2.41587	0.000909	3.10E-06	0.266722	0.015698

PRDM1|77111|AP	-2.41443	0.026081	0.001351	0.503388	0.01576
PIGV|1300|AP	2.413776	73.98941	2.246057	2437.353	0.015788
BBIP1|13092|ES	-2.41364	0.001045	3.97E-06	0.27525	0.015794
WDFY3|69811|ES	-2.41248	0.013505	0.000409	0.446	0.015844
ZNF227|50300|ES	-2.41235	0.012133	0.000337	0.43721	0.01585
PLD4|29579|RI	2.411802	43.44098	2.026989	930.996	0.015874
BCAT2|50814|ES	2.411178	21.38491	1.773817	257.8138	0.015901
WDR20|29338|AT	2.410738	99012719	31.2666	3.14E+14	0.01592
LRRC23|20002|ES	2.409977	626.562	3.328698	117938	0.015954
SYTL3|78284|ES	-2.40806	3.09E-15	4.80E-27	0.001996	0.016038
GRAP|39686|AP	-2.40724	2.53E-05	4.59E-09	0.139961	0.016074
VAV3|3931|AT	-2.40707	1.09E-08	3.60E-15	0.033207	0.016081
GPATCH2L|28540|AT	2.406491	3244.698	4.482316	2348801	0.016107
GPR162|19974|ES	-2.40545	4.48E-05	1.28E-08	0.156511	0.016153
C2orf68|54308|AT	-2.40509	6.55E-06	3.91E-10	0.10981	0.016169
C2orf68|54309|AT	2.405079	152610	9.106262	2.56E+09	0.016169
FDPS|8074|AA	-2.40139	5.75E-05	1.99E-08	0.166163	0.016333
ARID4A|27700|ES	-2.4008	6.31E-08	8.37E-14	0.04764	0.016359
MTUS1|82818|AP	-2.4008	0.043869	0.003417	0.563212	0.016359
HSFX2|90316|ES	-2.40019	0.041466	0.003083	0.557783	0.016386
YAF2|21105|AT	-2.39885	2.22E-11	4.39E-20	0.011242	0.016447
YAF2|21104|AT	2.398851	4.5E+10	88.94964	2.28E+19	0.016447
MSMO1|71042|ES	-2.39833	0.002085	1.34E-05	0.323581	0.01647
TSTD1|8525|RI	2.396377	26623693	22.50307	3.15E+13	0.016558
NARG2|30965|AA	-2.39636	0.001661	8.85E-06	0.311743	0.016559
SLMAP|65408|ES	2.39605	199.5502	2.621887	15187.63	0.016573
CCDC120|89058|AP	-2.39575	0.020268	0.000835	0.492047	0.016586
AURKAIP1|150|RI	2.395599	65383103	26.37717	1.62E+14	0.016593
MKL2|34068|AP	2.394986	30.93521	1.865194	513.0766	0.016621
TMEM44|68154|AP	2.394829	43.35805	1.982748	948.1391	0.016628
HCFC1R1|33353|AA	-2.39439	0.00405	4.46E-05	0.368049	0.016648
RNF167|38613|AD	-2.39324	4.29E-14	4.85E-25	0.003801	0.0167
SETD5|63091|ES	-2.39296	0.000902	2.89E-06	0.281205	0.016713
RCAN2|76416|AP	-2.39052	0.097899	0.014566	0.658006	0.016825
CLDND1|65747|AP	2.389879	451.6467	3.003114	67924.4	0.016854
MFSD9|54807|ES	-2.38917	0.003349	3.12E-05	0.359221	0.016886
PTPRE|13459|AP	2.388754	21.83657	1.739356	274.145	0.016906
ANKRD26|11054|AP	2.388259	66.70364	2.123887	2094.921	0.016928
ZFP64|59809|AP	-2.3876	0.047654	0.003917	0.579746	0.016959
MRPL27|42371|AP	-2.38752	1.80E-09	1.19E-16	0.027165	0.016962
MPPE1|44651|ES	-2.38668	0.002783	2.22E-05	0.349221	0.017001
SFTA3|27261|ES	2.386526	16741.18	5.687877	49274494	0.017008
GPR89A|7317|ES	2.384823	1650.859	3.743164	728083.7	0.017087
ZSCAN18|52401|AT	2.384498	6.64E+25	39565.76	1.11E+47	0.017102
GOLGA7|83514|AD	-2.38434	4.05E-05	9.94E-09	0.165291	0.01711
THOC7|65512|ES	2.383832	1.53E+15	501.2026	4.67E+27	0.017133
MBOAT7|51802|AT	2.383476	1.1E+18	1605.729	7.53E+32	0.01715
RFX5|7603|AP	2.382379	122.9825	2.347177	6443.786	0.017201
SERPINA1|29123|AA	-2.38234	0.075731	0.009062	0.632853	0.017203
ZNF655|80688|ES	2.380627	13.34277	1.580644	112.631	0.017283
PEMT|39496|AD	2.379049	3.64E+16	826.5552	1.60E+30	0.017357
FASTK|82340|ES	2.378893	152859.1	8.184035	2.86E+09	0.017365
SFTA3|121942|ES	2.376692	199.656	2.531225	15748.31	0.017469
CREM|11230|AP	2.375653	63.12678	2.065376	1929.426	0.017518
ZNF266|47339|AD	-2.37428	0.040447	0.002863	0.571343	0.017583
ZDHHC4|78753|AD	2.373097	55.23496	2.010493	1517.489	0.01764
CLUAP1|33575|AP	-2.37243	0.000816	2.29E-06	0.290471	0.017671
KIAA2013|694|AT	-2.37197	3.52E-22	6.60E-40	0.000188	0.017693

ZSCAN31|75728|AP	2.371506	7.667031	1.424016	41.28	0.017716
CCDC53|24023|ES	2.370419	46305.15	6.425217	3.34E+08	0.017768
TPM2|98133|ES	-2.36915	0.075933	0.009	0.640669	0.017829
C11orf49|15609|RI	2.368733	579.7272	2.998096	112099	0.017849
ARL13B|65700|ES	-2.36803	0.028008	0.001453	0.540047	0.017883
SLC25A19|43434|ES	2.367603	32.47817	1.820774	579.3313	0.017904
LONRF3|89946|AP	-2.36753	5.15E-12	2.33E-21	0.011398	0.017908
NDUFAF6|84598|ES	2.36707	59.86903	2.021418	1773.162	0.01793
IL1RN|55030|AP	2.366448	14.00873	1.57368	124.7041	0.01796
PAK6|29958|AP	2.366271	18.45497	1.649687	206.4548	0.017968
RASA4|81121|AP	2.366236	26.61737	1.756687	403.3071	0.01797
RASA4|81120|AP	-2.36621	0.037571	0.00248	0.569275	0.017971
DENND1B|9304|AT	-2.36611	0.000147	9.81E-08	0.219796	0.017976
DENND1B|9305|AT	2.366107	6810.995	4.549584	10196460	0.017976
LRRC6|85182|ES	2.364347	442.0797	2.834457	68949.51	0.018062
NR4A2|55613|AP	-2.36409	0.098797	0.014499	0.67322	0.018074
CCT7|53965|ES	-2.3638	0.057334	0.005357	0.613603	0.018089
SSBP1|82055|AD	2.362095	1185003	10.81462	1.3E+11	0.018172
CCDC124|48386|AP	2.362072	1215.512	3.350692	440944.8	0.018173
CCDC124|48385|AP	-2.36205	0.000823	2.27E-06	0.298466	0.018174
TCOF1|74070|ES	2.361936	511756.7	9.367254	2.8E+10	0.01818
HNRNPUL1|50036|AA	-2.36121	3.64E-11	7.86E-20	0.016827	0.018215
ZNF517|85650|AT	2.3611	46621745	20.08369	1.08E+14	0.018221
KBTBD3|18557|AT	2.360076	2741.701	3.827013	1964175	0.018271
DMKN|49178|ES	-2.35994	2.18E-05	2.92E-09	0.162095	0.018278
ATP11A|26310|ES	2.359618	77.2233	2.088009	2856.04	0.018294
KBTBD3|18556|AT	-2.35962	0.000365	5.09E-07	0.261679	0.018294
TRPT1|16581|ES	-2.35809	0.016299	0.000532	0.499058	0.018369
ITFG2|19715|AT	-2.35731	1.76E-10	1.36E-18	0.022679	0.018408
MROH7|3156|AP	2.356231	87.37602	2.120802	3599.85	0.018461
TRMT44|68771|AT	-2.35603	4.72E-11	1.21E-19	0.018372	0.018471
CENPK|72212|AT	-2.35574	0.021384	0.000872	0.524141	0.018486
CENPK|72213|AT	2.355735	46.76281	1.90787	1146.179	0.018486
KCNH6|42934|AT	2.355552	129.5581	2.263414	7415.922	0.018495
ZSCAN18|52406|AD	2.355252	132.3216	2.270278	7712.279	0.01851
ZNF263|33511|ES	-2.35496	0.050833	0.004259	0.606713	0.018525
IL15RA|10681|ES	-2.35292	0.007714	0.000134	0.443774	0.018627
HSP90AA1|29333|AT	-2.35109	1.13E-14	2.67E-26	0.004784	0.018718
TMEM44|68155|AP	-2.35084	0.024534	0.001115	0.539843	0.018731
ZNF331|51722|AP	-2.35065	0.002496	1.69E-05	0.369333	0.01874
FBXW7|70846|AP	2.350615	24.0192	1.696041	340.158	0.018742
SFTPB|54324|AA	-2.35034	0.001253	4.77E-06	0.329618	0.018756
ATG13|15597|AA	-2.34999	0.008677	0.000166	0.454808	0.018774
TTLL3|63225|ES	2.348154	198.0206	2.397097	16358.18	0.018867
XRCC6|62440|AP	-2.34793	0.001539	6.90E-06	0.342935	0.018878
SETD4|60518|ES	2.347307	40.81204	1.844189	903.1732	0.01891
TMC6|43757|AT	2.34724	56.78041	1.947282	1655.649	0.018913
TMC6|43758|AT	-2.34723	0.017612	0.000604	0.513543	0.018913
GTF2H2C|72395|ES	-2.34713	2.44E-07	7.33E-13	0.081134	0.018919
VPS53|38220|AD	-2.34688	5.80E-22	1.07E-39	0.000315	0.018931
TANGO2|61114|AP	-2.34662	0.000924	2.70E-06	0.31622	0.018944
HIATL1|86935|ES	-2.34615	2.10E-06	3.81E-11	0.116301	0.018969
CTNND1|15935|ES	2.346006	89.87487	2.096414	3853.004	0.018976
KBTBD3|18559|ES	-2.34481	0.000171	1.21E-07	0.240808	0.019037
EPOR|47690|AA	2.344523	3155.803	3.749207	2656319	0.019051
SELP|8933|ES	-2.3441	0.000205	1.70E-07	0.248723	0.019073
RARA|40856|AP	-2.34343	0.000133	7.58E-08	0.232005	0.019107
PDGFD|18490|AA	2.343416	1778.755	3.402635	929858.3	0.019108

PKIG|59473|AP	-2.34296	5.62E-06	2.28E-10	0.138585	0.019132
PAM|72898|ES	-2.34153	4.57E-05	1.06E-08	0.196231	0.019205
SIRT3|13596|AD	2.339184	30863.13	5.343331	1.78E+08	0.019326
PIGT|59561|ES	-2.33771	0.003766	3.50E-05	0.40579	0.019402
SERPINA1|29104|RI	-2.33691	0.002316	1.43E-05	0.375769	0.019444
STAU2|84165|ES	-2.33681	1.06E-07	1.50E-13	0.075046	0.019449
EEF1E1|75283|AT	2.336435	88000359	19.05893	4.06E+14	0.019469
SIDT2|18891|ES	2.335802	3157.558	3.656428	2726752	0.019502
SRP68|43546|AP	-2.3354	0.034548	0.00205	0.582158	0.019523
SRP68|43547|AP	2.335236	28.94009	1.717366	487.6822	0.019531
ST7L|4216|AA	-2.33466	5.80E-07	3.38E-12	0.099799	0.019561
MRPL27|42369|AP	2.33391	3.5E+08	23.3906	5.25E+15	0.0196
RANBP17|74506|ES	2.333897	9.573886	1.436112	63.82461	0.019601
UBE2V1|59758|ES	-2.33379	7.84E-07	5.84E-12	0.105191	0.019607
PXN|24748|ES	2.333752	24.84779	1.672931	369.0603	0.019609
SMARCB1|61329|AD	-2.33293	4.50E-06	1.45E-10	0.139712	0.019652
FRMD3|86681|AP	-2.33147	0.001676	7.77E-06	0.361154	0.019729
MRI1|95017|ES	2.330926	168.1192	2.260489	12503.52	0.019757
CD22|49112|ES	-2.33076	1.79E-05	1.82E-09	0.175672	0.019766
WDR45|89077|AA	2.329597	3300149	10.82143	1.01E+12	0.019827
SESN1|77156|AP	-2.32821	0.012523	0.000314	0.500172	0.019901
ANKRD16|10668|ES	2.327574	79798579	17.70175	3.6E+14	0.019935
STX16|59983|ES	-2.32696	0.007469	0.000121	0.461935	0.019968
U2AF1L4|49269|AA	-2.32685	4.72E-08	3.19E-14	0.069973	0.019973
NCBP2|68272|AD	2.326848	24960.04	4.935507	1.26E+08	0.019973
MFSD11|43691|AD	-2.32672	0.10871	0.016767	0.704839	0.01998
VCL|12254|ES	2.325098	44.61872	1.815685	1096.462	0.020067
COPS3|39475|AA	2.324464	12290.24	4.378101	34501279	0.020101
PBX1|8787|AT	-2.32437	4.17E-16	4.47E-29	0.003879	0.020106
MKNK1|2815|AD	-2.32427	0.013546	0.00036	0.509546	0.020111
TNFRSF12A|33347|ES	-2.32412	0.019716	0.000719	0.540535	0.020119
KSR1|39851|AA	2.323508	676.3355	2.77215	165009	0.020152
VAMP8|54289|AP	-2.32094	1.66E-20	3.30E-37	0.000839	0.02029
NVL|9944|ES	2.320885	172.0575	2.226743	13294.66	0.020293
ACOT7|391|AP	-2.32074	0.034184	0.001975	0.591656	0.020301
PGS1|43874|AA	-2.32036	1.34E-13	1.80E-24	0.010017	0.020321
POLR2D|55247|AP	-2.31966	0.001311	4.81E-06	0.35729	0.020359
NSUN5|270161|ES	2.319482	1.02E+20	1262.659	8.23E+36	0.020369
ARMCX4|89650|RI	2.319324	8.737355	1.399133	54.56334	0.020377
SLC25A32|84813|ES	-2.3188	0.000125	6.26E-08	0.248833	0.020406
KANK2|47639|ES	2.318714	187.4044	2.247208	15628.46	0.020411
HDAC10|62801|ES	-2.31803	2.44E-08	8.95E-15	0.066704	0.020448
IQCK|34333|AT	2.317684	1.83E+09	26.89179	1.25E+17	0.020466
SERF1B|72403|AT	-2.31763	5.00E-05	1.15E-08	0.216898	0.020469
AKIP1|14279|ES	2.31737	1008.013	2.905506	349711.7	0.020484
TOLLIP|13825|AP	-2.31661	7.20E-05	2.25E-08	0.230251	0.020525
RGS11|32858|AT	-2.31612	0.00648	9.11E-05	0.460769	0.020552
BBS5|55864|AT	-2.31593	1.28E-05	9.20E-10	0.176909	0.020562
AKAP13|32349|AT	-2.3159	4.67E-17	7.06E-31	0.003091	0.020564
IKBKG|90651|AP	-2.31534	1.19E-05	8.03E-10	0.175377	0.020594
RBMS3|63805|AA	2.313357	112.5946	2.057756	6160.859	0.020703
ASH2L|83369|AP	-2.31292	2.87E-05	4.06E-09	0.202691	0.020727
EMP1|20539|ES	-2.31244	8.29E-09	1.17E-15	0.058636	0.020753
ASH2L|83368|AP	2.312077	34815.79	4.916783	2.47E+08	0.020773
AP4M1|80892|ES	2.311086	858.6714	2.790836	264192	0.020828
ZNF302|48984|ES	-2.31059	0.000239	2.03E-07	0.282158	0.020855
FCER1G|8599|AT	-2.31022	3.42E-05	5.57E-09	0.210348	0.020876
FCER1G|8600|AT	2.310208	29224.16	4.753771	1.8E+08	0.020877

TJP2|86532|AP	-2.30933	0.013155	0.000333	0.519335	0.020925
RBM6|64943|ES	-2.30846	0.012609	0.000308	0.516739	0.020974
SMPD1|14103|AD	-2.30707	0.003919	3.54E-05	0.434391	0.021051
NEK5|25981|AT	2.307001	65.51846	1.875995	2288.209	0.021055
NEK5|25982|AT	-2.307	0.015263	0.000437	0.533051	0.021055
PDHA1|88633|ES	-2.30679	2.30E-11	2.11E-20	0.025152	0.021067
ABCB8|82306|ES	2.305329	18.83611	1.552417	228.5462	0.021148
RIBC1|89220|AT	-2.30502	0.001701	7.52E-06	0.384986	0.021165
ARL17A|42031|ES	-2.30496	0.046177	0.003379	0.631099	0.021169
ECHDC2|3026|ME	-2.30448	0.00495	5.42E-05	0.452217	0.021196
TRIP4|31124|AA	-2.30355	1.80E-11	1.30E-20	0.024977	0.021248
PRMT1|51043|ES	2.302864	4240.939	3.468449	5185477	0.021286
CDKN1C|13930|RI	2.299981	59.14984	1.827917	1914.039	0.021449
ZDHHC8|61145|ES	-2.29922	5.86E-13	2.19E-23	0.015671	0.021492
BAIAP2|44100|ES	-2.29841	0.000966	2.59E-06	0.359783	0.021539
NAGK|117487|ES	2.298275	86.81076	1.929134	3906.472	0.021546
WBP1|54068|ES	2.297683	3814.394	3.360521	4329567	0.02158
POLR3H|62429|AD	2.297537	131602.9	5.651517	3.06E+09	0.021588
ERG|60589|AP	-2.29748	0.037038	0.002226	0.616201	0.021591
UBL7|31724|AD	2.297453	626.8762	2.575714	152568.8	0.021593
PAK6|29956|AP	-2.29745	0.063713	0.006083	0.667339	0.021593
DDX11|20967|ES	2.297177	2.4E+10	33.39601	1.72E+19	0.021609
KCNC3|51173|AT	-2.29711	5.54E-05	1.29E-08	0.237296	0.021612
KCNC3|51172|AT	2.297111	18046.02	4.214145	77277574	0.021612
DECR1|84410|ES	2.29711	8.82E+09	28.81795	2.7E+18	0.021613
C10orf118|13186|AT	-2.29706	6.80E-06	2.65E-10	0.174443	0.021615
C10orf118|13185|AT	2.297055	147090	5.732422	3.77E+09	0.021616
MANBAL|59335|AP	-2.29677	0.000246	2.05E-07	0.295652	0.021632
RFC1|69056|AA	2.296578	1160.493	2.813135	478733.9	0.021643
KRTCAP3|53011|AP	2.296318	59.95506	1.821409	1973.532	0.021658
JKAMP|27751|AA	2.293638	38592.15	4.647666	3.2E+08	0.021811
METAP2|23795|AA	2.293315	186648.9	5.836936	5.97E+09	0.02183
SORT1|4009|AP	2.292955	785.2033	2.632813	234177	0.021851
IQCK|34334|AT	-2.29202	7.65E-10	1.23E-17	0.047785	0.021904
ABCD4|28376|RI	2.292011	8588.826	3.714622	19858801	0.021905
IRF5|81731|ES	-2.29046	7.09E-09	7.53E-16	0.066696	0.021995
KRTCAP3|53012|AP	-2.29011	0.016821	0.00051	0.554929	0.022015
SNAPIN|7752|ES	-2.29006	2.15E-10	1.15E-18	0.040414	0.022018
NSD1|74743|RI	-2.28937	0.008195	0.000134	0.500939	0.022058
LMO7|26069|RI	-2.28895	0.004004	3.55E-05	0.452292	0.022082
GOLGA4|63987|ES	-2.28811	0.068266	0.006849	0.680474	0.022131
ZNF233|50311|AT	-2.28799	0.000599	1.04E-06	0.345152	0.022138
ZNF233|50310|AT	2.287992	1668.703	2.897277	961099.3	0.022138
SLC23A3|57595|AT	2.287716	526.0028	2.453718	112759.1	0.022154
ZBTB8A|1609|AA	2.287294	383.1074	2.342574	62653.85	0.022179
PAQR4|33338|ES	-2.28682	1.10E-11	4.42E-21	0.027128	0.022206
EIF3H|84957|ES	2.286468	291.4299	2.248702	37769.05	0.022227
SLC23A3|57594|AT	-2.28622	0.001907	8.89E-06	0.409175	0.022242
ALG2|87057|ES	2.286117	208.6984	2.142487	20329.2	0.022247
DAP3|8121|ES	-2.28581	4.69E-11	6.52E-20	0.033696	0.022266
CCDC148|55662|AT	-2.2858	0.03103	0.00158	0.609548	0.022266
COPRS|40163|AP	-2.28566	3.60E-09	2.07E-16	0.062638	0.022274
MATN2|84632|AP	-2.28558	0.031552	0.001629	0.611174	0.022279
LCN6|88203|AP	2.284911	6.896291	1.316022	36.13832	0.022318
INSIG1|82433|ES	2.284623	210.4732	2.138653	20713.5	0.022335
GGNBP2|40493|AT	-2.28321	3.37E-13	6.61E-24	0.017145	0.022418
RANGAP1|62414|AP	2.28258	2691588	8.106001	8.94E+11	0.022455
PPP4R1|44608|ES	2.282447	185.6472	2.09189	16475.48	0.022463

CTNS|38480|AD	-2.28233	0.003173	2.27E-05	0.443705	0.02247
IQCK|93918|ES	-2.28049	0.03207	0.001668	0.616636	0.022578
TSPAN4|13789|AP	-2.27923	0.001075	3.01E-06	0.383875	0.022654
PHF20L1|85194|ES	2.279025	600.2849	2.448875	147145.9	0.022666
G3BP1|74194|AA	2.278438	12144.19	3.723072	39612785	0.022701
ZNF226|50296|ES	2.278297	2575.251	2.996223	2213427	0.022709
MKS1|42651|AA	2.27824	171.1236	2.05116	14276.45	0.022712
RUVBL2|50869|ES	-2.27823	0.187737	0.044523	0.791617	0.022713
CACTIN|46712|RI	2.276449	9.92E+09	24.53519	4.01E+18	0.022819
TMEM126A|18125|AD	-2.27528	1.57E-08	2.99E-15	0.082924	0.022889
SEC31A|69732|ES	-2.27428	0.054128	0.004384	0.668274	0.022949
SUSD4|9910|AT	-2.27395	0.002883	1.86E-05	0.445924	0.022969
CXorf23|88652|AD	2.273824	41.60354	1.672984	1034.591	0.022977
GPR61|4039|AT	-2.27238	0.001259	3.97E-06	0.399315	0.023064
NEK8|39970|ES	-2.27189	9.16E-12	2.75E-21	0.030514	0.023093
PPP3CB|12152|AP	-2.27064	0.002106	1.03E-05	0.430313	0.023169
DLG4|38844|AP	2.26974	316.6564	2.194231	45697.68	0.023223
YIPF2|47605|AD	-2.26859	3.26E-05	4.32E-09	0.245202	0.023293
TBC1D5|63665|ME	2.267519	3836.807	3.062753	4806489	0.023359
GIT2|24378|ES	-2.26721	1.13E-05	5.99E-10	0.213645	0.023378
MRPL48|17725|ES	2.266782	4004.358	3.073389	5217328	0.023404
BDH1|68299|ES	-2.26649	0.001954	8.88E-06	0.430157	0.023422
MYO1C|38306|AP	-2.26646	0.014504	0.000373	0.564126	0.023423
NCBP2|68271|AD	2.266305	224020.2	5.286993	9.49E+09	0.023433
NOL3|36955|ES	2.266143	806.942	2.470269	263596.9	0.023443
CLINT1|74394|AA	-2.26608	0.000394	4.48E-07	0.346825	0.023447
GSTT1|61365|ES	-2.26603	0.009354	0.000164	0.532044	0.02345
CENPV|39431|AA	-2.2651	0.012352	0.000276	0.553274	0.023507
SCML4|77137|AT	-2.26507	0.033083	0.001732	0.631814	0.023508
DLG4|38845|AP	-2.26407	0.003185	2.20E-05	0.461989	0.02357
ZKSCAN3|75740|ES	-2.26334	0.007197	0.0001	0.516149	0.023615
ARFGAP2|15645|ES	-2.26249	8.07E-07	4.25E-12	0.153203	0.023667
NSMF|88309|AT	-2.26164	0.001942	8.67E-06	0.434798	0.02372
MYO5B|45495|AP	2.261606	8.036504	1.320424	48.91261	0.023722
NFYC|2017|AA	2.260243	42583315	10.31723	1.76E+14	0.023806
METTL23|43631|AD	2.259765	54.53689	1.699828	1749.749	0.023836
DNPEP|57689|AD	2.258052	174.4561	1.97664	15397.31	0.023942
MTMR1|90351|RI	-2.25738	2.73E-12	2.49E-22	0.029956	0.023984
DDX31|87987|AA	-2.25731	2.77E-05	3.06E-09	0.251031	0.023989
PRKAG1|21509|ES	-2.257	9.59E-12	2.59E-21	0.035477	0.024008
TCEANC2|3123|AP	-2.2561	1.07E-09	1.71E-17	0.066416	0.024064
SH3KBP1|88642|AP	-2.256	0.009297	0.00016	0.541259	0.024071
FHL2|54831|AD	-2.25595	0.026012	0.001092	0.619534	0.024073
PFDN5|93147|ES	2.255749	248.0436	2.060547	29858.87	0.024086
TAF1C|37838|ES	-2.25545	2.57E-08	6.54E-15	0.101331	0.024105
C14orf80|29660|AP	2.255147	32.55552	1.577583	671.8262	0.024124
ARL6IP4|25030|ES	-2.25467	0.001849	7.79E-06	0.439318	0.024154
MYO5B|45493|AP	-2.25279	0.12503	0.020483	0.763176	0.024272
DIP2A|60938|AT	-2.25216	1.57E-05	1.03E-09	0.23804	0.024312
RHOG|14028|AP	-2.25188	1.97E-05	1.58E-09	0.245463	0.02433
ZNF222|50267|ES	-2.25153	0.001362	4.36E-06	0.42547	0.024352
DCAKD|41931|AT	-2.24904	2.15E-16	4.79E-30	0.009689	0.02451
ARL6IP4|25032|AD	-2.24879	1.62E-05	1.08E-09	0.242498	0.024526
CSAD|21968|ES	-2.24713	0.000284	2.29E-07	0.352164	0.024632
SORT1|4008|AP	-2.24683	0.00139	4.47E-06	0.431752	0.024651
FAM195B|44165|AP	-2.2463	1.13E-08	1.33E-15	0.097104	0.024685
MRPL21|17344|AD	2.245025	11617.34	3.282206	41119445	0.024766
NDUFS8|17276|AP	-2.24295	4.47E-14	9.67E-26	0.02069	0.0249

TMEM127|54517|AP	-2.24281	3.68E-07	8.76E-13	0.154356	0.024909
GLS2|22441|AA	2.242721	23.00449	1.48489	356.3945	0.024915
HKR1|49500|ES	-2.24272	0.001422	4.62E-06	0.437585	0.024915
SNCG|12440|AA	2.242104	433495.9	5.120861	3.67E+10	0.024955
SEMA4B|32469|AP	-2.24167	0.015805	0.000421	0.593811	0.024983
GHRL|63319|ES	-2.24136	0.029756	0.001376	0.643226	0.025003
CCDC25|83176|ES	-2.23972	7.93E-11	1.15E-19	0.054746	0.025109
COPS5|84054|AP	-2.23936	1.46E-14	1.14E-26	0.018791	0.025133
MFSD9|54806|AA	2.23927	664.5421	2.249341	196331.4	0.025138
ANP32E|7431|AA	-2.23853	5.91E-08	2.77E-14	0.126043	0.025187
RRP8|14161|ES	-2.23755	1.23E-06	8.19E-12	0.184848	0.02525
MYCL|1975|AT	-2.23734	2.11E-05	1.69E-09	0.263201	0.025264
MYCL|1976|AT	2.237275	47412.72	3.798213	5.92E+08	0.025268
NARG2|30959|AT	-2.23536	6.27E-07	2.29E-12	0.172133	0.025394
NARG2|30962|AT	2.235346	1593716	5.809024	4.37E+11	0.025395
NBPF10|4441|AP	2.235196	32870.77	3.598975	3E+08	0.025405
NBPF10|4439|AP	-2.23518	3.04E-05	3.33E-09	0.277877	0.025405
RFXANK|48609|AA	2.234312	105313.2	4.137176	2.68E+09	0.025463
RRNAD1|8313|RI	2.23284	533.3462	2.154114	132053.4	0.025559
SFI1|61871|ES	-2.23263	9.36E-18	1.05E-32	0.008324	0.025574
MROH7|3155|AP	-2.23157	0.014296	0.000343	0.596304	0.025643
MRPL47|67700|ES	-2.23141	3.87E-11	2.77E-20	0.054126	0.025654
C5orf45|74948|RI	2.230977	134.036	1.813049	9909.077	0.025683
SNCA|69932|AD	2.230534	42.92767	1.577812	1167.937	0.025712
SLC25A10|44158|AP	2.230145	4944.719	2.802488	8724476	0.025738
SLC25A10|44160|AP	-2.23014	0.000202	1.15E-07	0.356829	0.025738
TENC1|21923|AP	-2.22903	0.030131	0.001386	0.655246	0.025812
C14orf93|26685|RI	-2.22853	1.08E-05	4.66E-10	0.252141	0.025845
RAD1|71743|ES	-2.22828	0.000105	3.35E-08	0.331991	0.025862
CPNE1|59198|ES	2.227309	47.11581	1.587929	1397.984	0.025927
EFCAB11|28799|AT	2.227298	538366.8	4.873925	5.95E+10	0.025927
ASCC3|77091|AT	2.225866	44605374	8.19966	2.43E+14	0.026023
DMKN|49133|AP	2.224612	12.98435	1.356615	124.2751	0.026107
PPP6R3|92707|AA	2.224564	51.97847	1.599882	1688.726	0.026111
KCNH6|42933|AT	-2.22449	0.009711	0.000164	0.576308	0.026116
SPATA13|25473|AP	-2.22419	0.000652	1.02E-06	0.418339	0.026136
USB1|36625|AT	-2.22196	2.99E-11	1.56E-20	0.057427	0.026286
METTL3|26599|AA	-2.22026	3.36E-07	6.50E-13	0.174226	0.026401
ANKRD44|56672|AT	2.219138	411.2087	2.019747	83719.69	0.026477
LILRB1|51921|ES	-2.21868	1.37E-06	9.00E-12	0.207074	0.026508
IL32|33432|ES	-2.21849	1.42E-10	2.83E-19	0.071191	0.026522
MIF4GD|43426|ES	2.218476	32123.22	3.35093	3.08E+08	0.026522
DFFA|616|AT	-2.21778	4.60E-05	6.76E-09	0.313201	0.02657
DFFA|617|AT	2.217781	21724.55	3.192788	1.48E+08	0.02657
C11orf48|16385|AP	2.217566	7860705	6.324229	9.77E+12	0.026584
C11orf48|16384|AP	-2.21756	1.27E-07	1.02E-13	0.158125	0.026585
MTX2|56124|ES	2.217108	62.97127	1.616843	2452.545	0.026616
KLF3|69021|AT	-2.21636	0.000436	4.65E-07	0.408508	0.026667
ZNF701|51473|ES	2.215971	11.84713	1.330554	105.4859	0.026694
EFCAB11|28797|AT	-2.21546	1.74E-06	1.40E-11	0.216656	0.026729
POLR2H|67948|ES	-2.21497	3.82E-15	6.68E-28	0.021886	0.026762
C5orf45|74949|ES	-2.21492	6.77E-17	3.33E-31	0.013764	0.026766
AMACR|71701|RI	-2.2146	3.10E-16	5.83E-30	0.016475	0.026788
F11R|8516|AP	-2.21441	0.002377	1.13E-05	0.499435	0.0268
C20orf196|58657|AT	-2.21433	0.007172	9.07E-05	0.567119	0.026806
C20orf196|58656|AT	2.214326	139.4282	1.76329	11024.97	0.026806
F11R|8517|AP	2.214096	420.4808	2.00056	88377.3	0.026822
ATRIP|64659|ES	-2.21395	2.12E-05	1.54E-09	0.290887	0.026832

RNH1|13664|AP	-2.21386	0.003959	2.96E-05	0.530248	0.026838
RMND5B|74842|ES	2.213844	285631.4	4.223397	1.93E+10	0.02684
SNX1|31090|AT	-2.21308	2.06E-14	1.56E-26	0.027209	0.026892
LYSMD4|32723|ES	2.212302	29.42022	1.470672	588.54	0.026946
R3HDM1|55444|ES	-2.21176	0.016988	0.000459	0.6288	0.026983
GYG1|67206|ES	2.211296	2.72E+27	1312.762	5.64E+51	0.027015
TMEM184C|70795|AT	-2.21071	5.53E-13	7.50E-24	0.040708	0.027056
CEP78|86656|AT	-2.21064	0.008621	0.000127	0.583315	0.027061
CEP78|86655|AT	2.210625	115.9973	1.714296	7848.924	0.027062
PLXNB1|64637|AP	2.209548	167.4317	1.783192	15720.89	0.027137
CHTF18|33021|AA	-2.209	5.06E-08	1.70E-14	0.150465	0.027174
EXOC3|71443|ES	-2.20862	1.77E-09	3.02E-17	0.103415	0.027201
STAT3|41041|AA	2.208484	58.96193	1.582129	2197.362	0.027211
RSPH1|60716|ES	2.208207	50758.6	3.380542	7.62E+08	0.02723
C19orf60|48491|ES	-2.20817	0.006294	7.00E-05	0.56571	0.027233
ZNF720|36291|ES	2.208116	213.8003	1.827472	25013	0.027236
BOP1|85552|AT	-2.20617	9.65E-07	4.37E-12	0.213141	0.027372
TACC2|13341|ES	2.206047	24.97182	1.431808	435.5277	0.027381
SF3A1|61730|ES	2.206037	162.1871	1.764073	14911.32	0.027381
GEMIN6|53288|AD	-2.20522	4.43E-07	9.97E-13	0.196487	0.027439
PHKG2|36179|AA	2.204935	3333566	5.305231	2.09E+12	0.027459
C1orf50|2112|AA	-2.20476	1.74E-06	1.32E-11	0.229393	0.027471
ZSCAN18|52399|AP	2.204552	1167107	4.711136	2.89E+11	0.027486
ZSCAN18|52400|AP	-2.20454	8.57E-07	3.46E-12	0.212278	0.027486
RPL28|52096|AT	2.203652	10838.49	2.793846	42047026	0.027549
SEPT7|79241|AD	-2.20327	0.00459	3.82E-05	0.551821	0.027576
PFDN5|22011|ES	-2.20229	0.000346	2.87E-07	0.416034	0.027645
ZBTB7B|7877|AP	2.202211	31.36118	1.46085	673.2545	0.02765
CDK7|72327|ES	-2.20201	0.000302	2.23E-07	0.410339	0.027665
RBM4|17097|AD	2.201889	4.32E+09	11.44687	1.63E+18	0.027673
RASGEF1B|69694|AT	-2.20094	9.69E-08	5.51E-14	0.170646	0.02774
RASGEF1B|69695|AT	2.200907	10316292	5.858706	1.82E+13	0.027743
NUP50|62643|ES	2.199231	104.7924	1.658836	6619.968	0.027861
PLA2G6|62211|AD	2.198587	102.7086	1.653214	6380.935	0.027907
TCIRG1|17286|AD	-2.19844	4.76E-10	2.33E-18	0.097452	0.027918
ACOT8|59634|ES	-2.19836	3.23E-07	5.28E-13	0.197752	0.027923
PBLD|11926|AT	-2.19835	1.99E-06	1.64E-11	0.24082	0.027924
LARP7|70390|AD	-2.19834	1.91E-06	1.52E-11	0.2398	0.027925
MPPE1|44652|ES	-2.19816	0.000141	5.20E-08	0.382592	0.027938
RFC5|24685|ES	2.197169	24.8017	1.414319	434.9263	0.028008
CAPN3|30147|AP	-2.19705	0.00997	0.000163	0.608187	0.028017
MRPL2|76236|AT	2.196666	24899.02	2.976542	2.08E+08	0.028044
MRPL2|76235|AT	-2.19666	4.02E-05	4.80E-09	0.335976	0.028045
ZNHIT3|40469|AT	-2.19652	1.76E-08	2.13E-15	0.146216	0.028055
USP28|18808|ES	2.196475	54360.85	3.235074	9.13E+08	0.028058
ZNHIT3|40468|AT	2.196448	56665350	6.835537	4.7E+14	0.02806
CCDC36|64847|AT	-2.1958	0.008356	0.000117	0.598154	0.028106
CCDC36|64848|AT	2.195591	119.6355	1.671059	8565.01	0.028121
GUCD1|61412|ES	-2.19442	0.028491	0.001187	0.683754	0.028205
SLC39A13|15739|RI	2.193342	25284.54	2.940872	2.17E+08	0.028283
FAM184A|77361|AT	2.192612	67240.19	3.252656	1.39E+09	0.028335
SEMA4A|8191|ES	-2.1925	1.20E-10	1.63E-19	0.088685	0.028343
TMEM80|13726|AD	-2.19237	1.86E-08	2.28E-15	0.15151	0.028353
NPEPL1|59991|AP	-2.19234	0.000219	1.18E-07	0.409442	0.028355
CFLAR|56790|AT	2.192234	513433.9	4.02747	6.55E+10	0.028363
SCAMP5|31817|AP	2.191525	46.98747	1.501983	1469.939	0.028414
FHL1|90188|AP	-2.18968	0.00016	6.37E-08	0.39964	0.028548
DTD2|27119|RI	2.189133	557.4399	1.938589	160291.5	0.028587

TADA2A|40518|AT	-2.18874	0.000112	3.26E-08	0.386477	0.028616
NECAB3|59001|RI	2.188718	312.4854	1.822835	53568.85	0.028617
TADA2A|40520|AT	2.188254	8900.567	2.582403	30676888	0.028651
SERF1A|72416|AT	2.188037	6305.817	2.489265	15973924	0.028667
IRX2|71501|RI	2.187592	21.73268	1.377628	342.8425	0.028699
C14orf79|29592|ES	-2.18736	3.61E-06	4.81E-11	0.271808	0.028716
GORASP1|64152|ES	-2.1869	2.81E-12	1.24E-22	0.063281	0.02875
RFFL|40240|ES	2.186869	54.38905	1.513817	1954.113	0.028752
C2orf81|54050|AP	2.186794	51.1699	1.504082	1740.835	0.028758
C2orf81|54051|AP	-2.18679	0.019543	0.000574	0.664867	0.028758
LCN6|88202|AP	-2.18557	0.152296	0.028167	0.823445	0.028847
UBE2Q2|31898|ES	-2.18526	0.001048	2.23E-06	0.492953	0.02887
UAP1|8751|ES	-2.18503	0.0462	0.00293	0.728538	0.028887
SHF|30416|ES	-2.18481	0.003337	2.00E-05	0.556044	0.028902
PPIG|55876|AD	2.184136	5.430599	1.189658	24.78981	0.028952
SLC9B2|70167|AP	-2.18402	0.021274	0.000672	0.673687	0.028961
VPS28|85598|AP	-2.18318	9.40E-10	7.40E-18	0.119412	0.029022
VPS28|85599|AP	2.183069	1.06E+09	8.365867	1.35E+17	0.029031
SUPT4H1|42666|AD	2.183002	33670575	5.875734	1.93E+14	0.029036
MBD4|66720|AD	-2.18265	4.95E-05	6.74E-09	0.363712	0.029062
OVOL1|16917|AP	-2.18209	0.008427	0.000115	0.614955	0.029103
HAUS3|68549|ES	-2.18144	0.001248	3.07E-06	0.507223	0.029151
TMEM194B|56574|ES	-2.1811	8.23E-05	1.76E-08	0.385362	0.029176
FAM184A|77360|AT	-2.18087	1.54E-05	7.26E-10	0.325423	0.029193
CAD|52954|ES	-2.18058	0.000366	2.99E-07	0.449097	0.029215
CASC1|20784|AA	2.17997	704100.6	3.891812	1.27E+11	0.02926
RAMP2|41123|ES	-2.17689	1.11E-11	1.52E-21	0.08096	0.029489
FLNB|65419|ES	-2.1757	0.010327	0.000168	0.635436	0.029578
C17orf75|40200|AA	-2.17532	8.42E-05	1.79E-08	0.394987	0.029606
PTPN6|20029|AA	-2.17479	0.010357	0.000168	0.636713	0.029646
FAM65B|75540|AT	2.174311	2347.112	2.149181	2563272	0.029682
EIF6|59071|AP	-2.1741	1.21E-05	4.48E-10	0.327895	0.029697
EIF6|59072|AP	2.174099	82534.51	3.049698	2.23E+09	0.029698
PTRH2|42790|AP	-2.17397	0.000483	4.94E-07	0.471555	0.029708
PTRH2|42789|AP	2.173954	2072.205	2.120551	2024961	0.029709
FGF12|68129|AP	-2.1734	0.106014	0.01401	0.802206	0.02975
TECPR2|29416|AT	2.173282	77130.48	3.017897	1.97E+09	0.029759
PPP4R1|44609|ES	-2.17313	0.032884	0.001512	0.715362	0.02977
UBOX5|58571|ES	-2.17287	0.001647	5.08E-06	0.533683	0.02979
DCAF10|86447|ES	-2.1727	0.000542	6.14E-07	0.478881	0.029803
TECPR2|29415|AT	-2.1727	1.30E-05	5.08E-10	0.332341	0.029803
ANAPC13|66872|AD	2.172309	16029.35	2.57652	99723724	0.029832
FN1|57398|ES	-2.17223	0.018383	0.000499	0.676706	0.029838
YBEY|60921|AD	2.171604	1395.741	2.025285	961885.3	0.029886
POLR2H|67947|ES	-2.17141	0.03396	0.001603	0.719362	0.0299
LGMN|29005|ES	2.17139	21.13809	1.345924	331.9795	0.029902
SETD3|29218|ES	-2.17065	4.31E-08	9.64E-15	0.192804	0.029958
TM2D3|32771|ES	-2.16942	0.00089	1.56E-06	0.507557	0.030051
RPL39L|68070|AP	2.169133	24.8337	1.363087	452.438	0.030073
RPL39L|68071|AP	-2.16911	0.040271	0.002211	0.733661	0.030075
KIAA1407|66238|AD	2.168922	80.79957	1.526733	4276.17	0.030089
SUSD4|9911|AT	2.168311	205.9889	1.66852	25430.59	0.030135
DMKN|49132|AP	-2.16822	0.079734	0.008106	0.784337	0.030142
TMEM9B|14289|ES	-2.1682	7.73E-14	1.08E-25	0.055043	0.030143
ARL13B|65699|ES	-2.16762	0.083402	0.008825	0.788227	0.030188
ANKS1B|23885|AT	2.167318	795.8764	1.894666	334317.1	0.030211
ANKS1B|23886|AT	-2.16732	0.001256	2.99E-06	0.5278	0.030211
FAM71D|28039|AT	-2.16627	0.094108	0.011092	0.798458	0.03029

DYNLT1|78273|AP	2.165558	78263.3	2.914647	2.1E+09	0.030345
DYNLT1|78274|AP	-2.16556	1.28E-05	4.76E-10	0.343095	0.030345
MYEF2|30479|AT	2.165346	13973.3	2.472746	78962122	0.030361
ZNF235|50306|AT	2.164731	63859.24	2.847984	1.43E+09	0.030408
SLC43A3|15833|AP	2.164337	125.8811	1.578681	10037.52	0.030438
LMBR1L|21521|AD	2.163073	2.95E+08	6.241953	1.4E+16	0.030536
MTFR1L|1212|AA	-2.16259	1.71E-07	1.26E-13	0.232263	0.030573
TM7SF2|16763|ES	-2.16244	1.04E-15	2.76E-29	0.039559	0.030584
RNF213|44046|AT	-2.16218	4.05E-05	4.22E-09	0.388304	0.030605
ANKRD26|11053|AP	-2.16207	0.017336	0.000439	0.684516	0.030613
AUH|86823|AT	-2.16138	7.09E-06	1.52E-10	0.331235	0.030666
AUH|86822|AT	2.161321	140933.6	3.017879	6.58E+09	0.030671
TMC6|43765|ES	-2.16011	1.67E-06	9.59E-12	0.291574	0.030764
GPR107|87896|ES	-2.15886	0.001056	2.10E-06	0.531867	0.030861
TMEM44|68161|ES	2.158633	106.0336	1.536071	7319.409	0.030879
PARP3|65117|AA	-2.15858	0.023431	0.000775	0.707949	0.030883
ZNF784|52117|AD	2.15844	713.5991	1.829721	278306.7	0.030894
RTEL1|60145|AT	2.158433	135.8107	1.570811	11742.05	0.030894
RTEL1|60144|AT	-2.15843	0.007363	8.52E-05	0.636616	0.030894
TMEM260|27646|ES	-2.15762	0.004651	3.54E-05	0.611402	0.030957
PACRGL|68883|ES	-2.15732	0.009156	0.000129	0.65093	0.030981
MLK4|10329|AT	-2.15708	1.04E-08	5.77E-16	0.186381	0.030999
ASCC3|77092|AT	-2.15664	2.88E-08	4.04E-15	0.205271	0.031033
BZW2|78864|ES	-2.15664	2.18E-11	4.47E-21	0.106609	0.031034
SLC25A35|39149|AT	-2.15663	0.000744	1.07E-06	0.518436	0.031035
SLC25A35|39151|AT	2.156627	1344.866	1.928879	937677.1	0.031035
UBE2D4|79369|AT	-2.15647	1.31E-07	7.32E-14	0.236023	0.031047
UBE2D4|79371|AT	2.156428	7605208	4.235572	1.37E+13	0.03105
KAT8|36242|RI	2.155384	2287377	3.772041	1.39E+12	0.031132
CCDC84|19052|AD	2.155276	127.6528	1.55185	10500.52	0.03114
CDC42|1006|ES	2.154319	11729.89	2.328717	59084189	0.031215
BTN3A1|75662|AA	2.152952	50337.88	2.6392	9.6E+08	0.031322
ZC3H11A|9454|AP	2.152618	25.59946	1.336698	490.2624	0.031349
SESN1|77155|AP	2.152081	85.20934	1.487084	4882.464	0.031391
PHC3|67605|AA	-2.15169	0.00091	1.55E-06	0.535851	0.031422
EPN1|52138|AA	2.151387	47197864	4.817258	4.62E+14	0.031446
STK11IP|57739|AD	2.151285	71.70535	1.462245	3516.275	0.031454
EEF1E1|75282|AT	-2.15108	3.59E-07	4.82E-13	0.267546	0.03147
STARD10|17647|AD	2.150003	116.5838	1.522895	8924.967	0.031555
MED11|38579|AA	2.149943	865.5456	1.817828	412123.2	0.03156
STAU2|84155|AT	2.148718	224.802	1.609139	31405.57	0.031657
GCNT2|75296|AP	-2.14859	0.03868	0.001991	0.751611	0.031667
LETMD1|21754|ES	-2.14848	0.0105	0.000164	0.670462	0.031676
SLC25A17|62379|ES	-2.14797	1.31E-09	1.03E-17	0.166974	0.031716
GLI4|85408|AA	-2.14772	0.000925	1.57E-06	0.542953	0.031736
MVP|35966|AA	2.147397	2550.161	1.983074	3279414	0.031762
TTLL3|63216|AA	2.147272	15.01168	1.266545	177.9255	0.031772
TAB1|62302|AT	-2.14726	2.12E-10	3.13E-19	0.143263	0.031773
LYRM1|34404|AP	-2.14697	0.003136	1.62E-05	0.605238	0.031796
TMEM136|19161|AA	2.145904	22.67593	1.310563	392.3488	0.031881
PDE4D|72140|AP	-2.14543	0.060535	0.00467	0.784705	0.031918
PPP1R1A|22195|AT	-2.14465	0.039636	0.002074	0.757312	0.031981
PRICKLE3|89098|AT	2.144584	9637.46	2.202753	42165704	0.031986
PRICKLE3|89099|AT	-2.14446	0.000104	2.38E-08	0.454251	0.031996
CDPF1|62699|AD	-2.14387	0.000632	7.52E-07	0.531598	0.032043
PPP1R1A|22194|AT	2.143678	25.20989	1.318607	481.9774	0.032059
USP2|19141|ES	-2.14288	0.012229	0.000218	0.686653	0.032123
EXOC6B|53943|AT	-2.14287	1.47E-14	3.28E-27	0.065969	0.032123

APTX|86073|AP	2.142648	575.3341	1.719149	192542.5	0.032141
GAB1|70700|ES	-2.14212	0.017425	0.000428	0.708653	0.032183
GNRH1|83105|RI	-2.14198	0.000601	6.79E-07	0.532483	0.032195
HOMER3|48536|AP	2.14187	6383.389	2.10454	19361781	0.032204
ANKMY1|58265|AD	-2.14186	0.053072	0.003614	0.779305	0.032204
ZNF611|51548|ES	-2.14139	0.002515	1.05E-05	0.602247	0.032243
FAH|32179|RI	2.140256	24.44824	1.309013	456.6161	0.032334
ZDHHC15|89528|ES	-2.1402	0.006641	6.73E-05	0.655545	0.032339
SYT9|14193|AT	2.139516	31.15346	1.334558	727.2355	0.032394
RNF7|67078|ES	2.138825	190.5123	1.551175	23398.35	0.03245
IL17RC|63262|ES	2.138777	11829.05	2.190387	63882036	0.032454
APCDD1|44629|ES	-2.13873	3.76E-05	3.30E-09	0.426683	0.032458
COL1A1|402674|ES	2.138614	621.3594	1.711367	225601.8	0.032467
MYEF2|30481|AT	-2.13838	0.000572	6.09E-07	0.536317	0.032486
MAT2B|74448|AP	2.138155	15892.26	2.239342	1.13E+08	0.032504
MAT2B|74449|AP	-2.13815	6.29E-05	8.87E-09	0.446574	0.032505
THOC5|61617|ES	2.137864	53.05756	1.391626	2022.888	0.032528
ZNF415|51678|ES	2.137862	35.90685	1.347135	957.0692	0.032528
ACADM|3492|ES	-2.13778	5.07E-05	5.86E-09	0.43932	0.032535
ASCC1|12079|ES	2.137501	94.40358	1.458946	6108.545	0.032557
LRRFIP2|63981|AD	-2.13733	0.000114	2.76E-08	0.470743	0.032572
ZNF726|48832|AT	2.136786	786.9287	1.736373	356638.1	0.032615
DAPK2|31076|ES	2.136667	757.434	1.730307	331563.3	0.032625
NPRL2|65036|AA	-2.13644	0.000103	2.24E-08	0.468243	0.032643
TCTN1|24462|AA	-2.13628	1.52E-10	1.49E-19	0.154754	0.032656
NQO1|37304|ES	2.136229	3.38E+12	10.80808	1.05E+24	0.032661
PET100|47118|AT	-2.13607	4.67E-12	1.87E-22	0.116374	0.032674
SLAMF7|8485|ES	-2.13603	0.000235	1.10E-07	0.502247	0.032677
LAMA4|77295|AT	-2.1349	1.76E-10	1.95E-19	0.158747	0.032769
DMKN|49200|ES	-2.13464	0.006184	5.80E-05	0.65957	0.03279
MAEA|68467|AP	-2.13398	1.12E-14	1.72E-27	0.07284	0.032844
DCTD|96837|ES	2.133212	5763.937	2.020343	16444227	0.032907
ZCCHC11|3009|AA	2.133137	104.5927	1.458639	7499.891	0.032913
ACP5|47750|ES	-2.13312	0.018021	0.00045	0.721794	0.032915
NVL|9942|ME	2.132942	16.15359	1.253108	208.2331	0.032929
NMRAL1|33740|AD	2.132768	36.86892	1.339481	1014.81	0.032944
PDCD11|13007|ES	2.132447	12.76821	1.228767	132.6755	0.03297
SH3KBP1|88643|AP	2.132367	18.28066	1.264831	264.2111	0.032977
EIF4E3|65610|AP	-2.13191	0.002986	1.43E-05	0.625699	0.033015
MXI1|13082|ES	2.131806	3.84E+11	8.586619	1.72E+22	0.033023
LCK|1583|ES	-2.13173	0.000921	1.49E-06	0.56936	0.033029
ANGPTL1|9095|ES	-2.12963	0.012636	0.000226	0.705916	0.033202
KIAA0226L|25827|AP	-2.12956	0.077488	0.007361	0.815722	0.033208
NT5DC2|65224|AP	2.12954	238.0957	1.546185	36664.14	0.03321
NT5DC2|65225|AP	-2.12954	0.0042	2.73E-05	0.646753	0.03321
ZNF574|50117|AP	-2.1292	0.006073	5.53E-05	0.666527	0.033238
ZSCAN31|75730|AP	-2.12881	0.157126	0.028592	0.86348	0.03327
RILP|38360|ES	2.127505	9.36E+09	6.098566	1.44E+19	0.033378
TSR1|38389|ES	-2.12744	0.000203	8.07E-08	0.512107	0.033384
EPOR|47694|ES	-2.12661	0.002005	6.54E-06	0.614605	0.033453
PHYHD1|87799|ES	-2.12619	7.94E-05	1.32E-08	0.478041	0.033488
IL15RA|10678|AA	2.125546	586.8661	1.643129	209607.3	0.033541
APOC1|50357|AP	-2.12413	0.014352	0.000286	0.720365	0.033659
ACADM|3510|ES	-2.12252	0.001788	5.19E-06	0.615986	0.033794
ERN1|43052|AT	2.122168	161.5572	1.474991	17695.52	0.033824
ERN1|43053|AT	-2.12217	0.00619	5.65E-05	0.677972	0.033824
IER2|47930|AP	2.121888	133.0811	1.452425	12193.8	0.033847
IER2|47929|AP	-2.12189	0.007514	8.20E-05	0.688504	0.033847

CNDP2|45811|AP	-2.12107	0.00068	8.04E-07	0.574642	0.033916
RAG1|15433|AT	2.120729	9.898287	1.189789	82.34748	0.033945
DYRK1B|49843|AT	-2.12038	2.28E-19	1.34E-36	0.038876	0.033974
COPS4|69764|ES	2.120268	5.29E+13	10.90395	2.57E+26	0.033983
CLSPN|1730|AT	-2.11986	0.021187	0.0006	0.747715	0.034017
CLSPN|1731|AT	2.119849	47.19597	1.337367	1665.555	0.034019
MCFD2|53472|AP	2.119621	3619.34	1.853739	7066595	0.034038
HS1BP3|52778|AT	-2.11836	6.09E-05	7.68E-09	0.483975	0.034144
RAG1|15432|AT	-2.11783	0.101218	0.012153	0.843045	0.03419
WDR27|78474|ES	2.117667	13.13743	1.211422	142.4707	0.034203
ANKRD10|26274|ES	-2.11742	0.024359	0.000782	0.758625	0.034224
FAM21A|11563|ES	-2.11729	0.017781	0.000427	0.741248	0.034235
MFF|57798|ES	-2.117	0.003497	1.86E-05	0.657338	0.03426
METTL23|43632|ES	-2.11698	0.09395	0.010519	0.839116	0.034262
SCAP|64519|ES	2.116411	1.36E+18	21.90216	8.47E+34	0.03431
GSTT1|61384|ES	-2.11553	2.58E-06	1.71E-11	0.388191	0.034384
STAU2|84157|AT	-2.1153	0.005558	4.52E-05	0.68297	0.034404
FUZ|51084|AD	-2.11484	6.08E-06	8.90E-11	0.414935	0.034443
CCDC41|23730|ES	2.113966	2254.658	1.754978	2896608	0.034518
UBXN4|55450|ES	-2.1138	0.0304	0.001192	0.775511	0.034532
IFI27|29088|AA	2.113617	28160.69	2.106078	3.77E+08	0.034548
CCT7|53961|ES	-2.11352	0.076407	0.007037	0.829578	0.034557
PIGQ|32903|ES	-2.11299	0.052767	0.003445	0.808107	0.034601
IMMP1L|14819|ES	-2.11283	0.00288	1.27E-05	0.654918	0.034616
SERF1B|72405|AT	2.111578	11406.68	1.955733	66528736	0.034723
STX16|59984|ES	-2.11114	0.010507	0.000153	0.721638	0.03476
ATG4B|58404|ES	2.109741	12539.8	1.954115	80469484	0.034881
CHMP7|83076|AD	-2.10956	0.000374	2.45E-07	0.571448	0.034896
TET2|70187|AT	-2.10949	0.006099	5.34E-05	0.69665	0.034902
TET2|70188|AT	2.109489	163.9505	1.435435	18725.88	0.034902
CSTF3|14887|ME	-2.10948	0.00272	1.12E-05	0.65791	0.034903
RHEB|82378|AP	2.108106	4385.84	1.802752	10670130	0.035022
GOLGA7|83513|AA	2.107703	30160.39	2.060558	4.41E+08	0.035057
RASSF3|22860|ES	-2.10763	7.42E-06	1.26E-10	0.437141	0.035063
HLTF|67209|AA	-2.10715	0.011719	0.000187	0.733015	0.035105
TLE1|86673|AT	2.10655	2327.467	1.715091	3158492	0.035157
UBB|39435|AD	2.10649	13.05785	1.195692	142.6015	0.035162
CD44|15056|ES	-2.10646	0.040925	0.002092	0.800699	0.035164
TENC1|21925|AP	2.106292	32.27729	1.272993	818.4053	0.035179
FPGT-TNNI3K|3457|AT	2.106035	9.113527	1.165634	71.25424	0.035201
FAM204A|13246|ES	-2.10544	9.56E-12	5.27E-22	0.17321	0.035253
C11orf54|18326|ES	2.105391	29.82271	1.264306	703.4638	0.035257
BCL2L1|58902|AA	-2.1053	0.000377	2.45E-07	0.580308	0.035265
NAT9|43294|ES	-2.10517	0.040881	0.002084	0.802104	0.035277
ZNF587B|52344|AP	2.105076	47.79737	1.305476	1750.005	0.035285
NDUFB10|33166|RI	2.10499	1808.993	1.676584	1951860	0.035292
BRMS1L|27244|AT	2.104484	1.13E+47	1703.185	7.50E+90	0.035336
GEMIN7|50398|ES	2.104059	42.32568	1.2924	1386.152	0.035373
JUP|40932|RI	2.103523	5411.634	1.797993	16288038	0.03542
ZC3H11A|9455|AP	-2.10349	0.040404	0.002032	0.803369	0.035423
DNAJB12|12088|AP	-2.10344	2.17E-10	2.15E-19	0.219196	0.035427
CHTOP|7751|AA	-2.10341	2.56E-05	1.34E-09	0.486207	0.03543
ZNF397|45149|AD	2.103055	9.536109	1.165832	78.00211	0.035461
YIPF1|3074|ES	2.102989	4635.187	1.775543	12100502	0.035467
NSRP1|40075|AT	-2.10295	5.06E-11	1.28E-20	0.199498	0.03547
OXSR1|64042|ES	2.101733	4468.224	1.762843	11325468	0.035577
SAC3D1|16732|RI	2.101725	25.19983	1.243151	510.824	0.035577
FEZ2|53197|ES	2.10108	4.01E+08	3.783039	4.26E+16	0.035634

ZNF268|25351|ES	-2.10084	0.003128	1.44E-05	0.679264	0.035655
EPS15L1|48161|ES	2.100025	113.6537	1.371187	9420.431	0.035727
ZNF177|47313|ES	-2.09981	0.021821	0.000614	0.775119	0.035745
SCIN|78817|ES	-2.09965	0.000112	2.32E-08	0.546113	0.03576
EIF4G1|67892|ES	-2.09918	0.064065	0.004925	0.833406	0.035801
NACA|22488|AD	2.099168	57.3886	1.308081	2517.773	0.035802
CES4A|36915|ES	-2.09913	2.17E-06	1.12E-11	0.421188	0.035805
ALS2CL|64466|RI	2.098106	199.6737	1.417283	28131.01	0.035896
ZNF675|48822|AT	2.097895	311761.2	2.297227	4.23E+10	0.035914
DMKN|49142|AD	2.097749	2050.572	1.65018	2548112	0.035927
DNAJC17|30040|AA	2.097052	5.11E+08	3.709055	7.03E+16	0.035989
ADAM15|7897|ES	-2.09704	0.01933	0.000484	0.772643	0.03599
LRRC23|20001|ES	2.096878	4566.826	1.733609	12030335	0.036004
RAB28|265743|ME	2.096127	1896.563	1.632809	2202922	0.036071
PRR14|36165|AP	2.095659	1762.586	1.622518	1914746	0.036112
PRR14|36164|AP	-2.09565	0.000567	5.22E-07	0.616343	0.036113
DHRS4|26788|ES	2.095549	17.13972	1.201827	244.4363	0.036122
CEACAM1|50164|ME	-2.09553	0.005434	4.14E-05	0.713649	0.036124
MMAB|24331|ES	2.095261	1.78E+14	8.320968	3.80E+27	0.036148
DDO|77229|ES	2.095217	72.04911	1.318002	3938.593	0.036152
HAX1|7823|ES	-2.09483	0.002359	8.22E-06	0.677414	0.036186
RUVBL2|50864|AD	2.094475	44.64542	1.276298	1561.715	0.036218
TSTD1|8526|ES	-2.0941	0.009731	0.000127	0.743242	0.036251
TFDP2|67090|AA	2.094091	199.5153	1.403827	28355.59	0.036252
SEPT2|58377|ES	-2.09354	4.89E-07	6.04E-13	0.395682	0.036301
C18orf32|45473|AP	-2.09354	1.28E-13	1.10E-25	0.150482	0.036301
ZMYND8|59711|AP	2.092933	112.9493	1.350283	9448.047	0.036355
GCNT1|86638|ES	-2.0928	0.008707	0.000102	0.740006	0.036367
GRK6|74764|AT	-2.09246	2.37E-10	2.28E-19	0.245746	0.036398
NFE2L1|42157|AP	-2.09235	0.009339	0.000117	0.744005	0.036407
CSF2RA|88365|ES	-2.09147	0.000285	1.35E-07	0.598472	0.036486
SNX5|58748|RI	2.09075	41.61888	1.262683	1371.787	0.03655
NUDT18|82937|RI	2.090638	106.7192	1.338981	8505.709	0.03656
ACP6|7383|AT	-2.08846	0.026835	0.0009	0.800432	0.036757
ACP6|7384|AT	2.088436	37.26402	1.249279	1111.527	0.036759
DDX17|62239|AD	-2.08791	0.00014	3.38E-08	0.58055	0.036806
CREM|11244|AD	-2.08755	0.024165	0.000733	0.796499	0.036839
LTBP4|49936|ES	2.086965	1046.439	1.526734	717239.9	0.036891
TRIM14|87027|AP	-2.08681	3.38E-07	2.82E-13	0.404211	0.036905
PTBP1|46317|ES	-2.08658	5.06E-06	5.38E-11	0.477162	0.036926
FAM110A|58466|AP	-2.08651	0.004727	3.09E-05	0.72271	0.036932
ZNF302|48983|AA	2.086256	49.66968	1.266698	1947.645	0.036955
MATN2|84633|AP	2.085962	17.25736	1.187727	250.7449	0.036982
LUC7L|32842|RI	2.085806	4845607	2.531297	9.28E+12	0.036996
PQLC1|46259|ES	-2.08535	0.00091	1.26E-06	0.656391	0.037037
TARDBP|634|ES	-2.08509	6.01E-09	1.13E-16	0.321104	0.037061
RHEB|82377|AP	-2.08448	0.000238	9.29E-08	0.607456	0.037117
MFSD10|68614|RI	2.084317	6270.457	1.684814	23337070	0.037131
VKORC1|36233|ES	2.084291	1.01E+17	10.33409	9.85E+32	0.037134
TLE1|86675|AT	-2.08419	0.000478	3.61E-07	0.634021	0.037143
ABCD4|28386|ES	-2.08415	0.002118	6.47E-06	0.692877	0.037146
BECN1|41142|AD	-2.0841	0.000829	1.05E-06	0.655331	0.037151
RABL3|66379|ES	-2.08351	9.59E-09	2.75E-16	0.334597	0.037204
EVI5L|47191|ES	2.082833	385.6406	1.420895	104665.5	0.037266
RIBC1|89219|AT	2.082828	161.6803	1.349852	19365.48	0.037267
P4HA2|73262|ES	-2.08114	0.008703	9.98E-05	0.758643	0.037421
PTBP1|46318|ES	-2.08102	1.61E-05	4.90E-10	0.52613	0.037432
FAM173B|71547|ES	-2.08076	0.004287	2.52E-05	0.728678	0.037456

BCAT2|50816|ES	2.079857	52.62314	1.256657	2203.621	0.037539
HNRNPH3|11928|AD	-2.07984	6.34E-06	8.00E-11	0.501642	0.03754
LSR|49088|ES	-2.07914	0.000242	9.44E-08	0.620476	0.037605
GNB2L1|127737|ES	2.079059	22.61207	1.195586	427.6611	0.037612
SULT1A2|35758|AA	2.079053	810.4693	1.46762	447568.6	0.037612
CACTIN|46714|ES	-2.07894	0.015222	0.000294	0.787016	0.037623
SMAGP|21829|AP	-2.07889	0.002772	1.08E-05	0.714021	0.037627
TAF6|80899|AD	2.077961	5427.791	1.629559	18079073	0.037713
ZNF419|52268|AT	2.077858	791694.3	2.161089	2.9E+11	0.037722
ZNF419|52269|AT	-2.07785	1.26E-06	3.45E-12	0.462741	0.037723
WDR91|81880|AP	-2.0776	9.33E-05	1.47E-08	0.591297	0.037746
RBMS2|22465|ME	2.077502	6047.128	1.636619	22343472	0.037755
CYP51A1|80417|AP	-2.0771	0.017218	0.000373	0.795277	0.037792
TRIM9|27505|AT	2.076525	28.4398	1.206738	670.2554	0.037845
HCFC1R1|33354|ES	-2.07604	8.89E-06	1.51E-10	0.521889	0.03789
CRYGN|152609|AD	2.076014	184.0394	1.338474	25305.32	0.037893
ISLR|31676|AP	2.075973	22.75936	1.190805	434.9904	0.037896
ETV4|41711|AP	2.075532	239.0056	1.356538	42109.89	0.037937
ELOF1|47744|ES	2.074869	1.38E+12	4.702836	4.07E+23	0.037999
AKAP8L|48085|ES	-2.0748	0.001102	1.77E-06	0.685949	0.038005
CACNB1|40629|ES	-2.07459	0.003628	1.80E-05	0.733113	0.038025
DNAH9|39292|AT	-2.07437	0.018643	0.000433	0.802814	0.038045
ANKDD1A|31138|AT	-2.07415	0.000135	2.97E-08	0.612288	0.038065
ANKDD1A|31137|AT	2.074142	7411.177	1.633169	33631277	0.038066
VSTM4|11499|AT	2.073591	1651.773	1.500843	1817883	0.038117
VSTM4|11498|AT	-2.07357	0.000605	5.50E-07	0.666341	0.038119
TMEM14B|75312|AT	-2.07326	6.88E-06	9.05E-11	0.522259	0.038148
RPS15|46490|RI	2.072975	31.64856	1.207241	829.6861	0.038175
ARAP3|73825|ES	-2.07218	0.002244	7.00E-06	0.718703	0.038249
LSM14A|48951|ES	2.072032	8613.166	1.632442	45445188	0.038262
ISLR|31677|AP	-2.07164	0.044079	0.002299	0.845114	0.038299
C18orf8|44832|ES	-2.07109	4.41E-13	8.95E-25	0.217293	0.03835
CREBRF|74575|AT	-2.07061	5.89E-13	1.56E-24	0.22206	0.038395
EPOR|47693|ES	-2.06924	0.129402	0.018654	0.897637	0.038523
AP1G1|37491|ES	-2.06892	4.32E-05	3.17E-09	0.589065	0.038553
TGFBR3|3742|AP	-2.06883	0.000309	1.46E-07	0.653586	0.038562
ZNF415|51679|ES	2.068313	15.22895	1.153333	201.0875	0.038611
NDEL1|39184|AP	-2.06826	1.28E-09	4.80E-18	0.342285	0.038615
NCBP2|68263|AP	-2.06808	9.45E-10	2.64E-18	0.337459	0.038633
ZNF585B|101324|ME	2.067359	15064.27	1.648301	1.38E+08	0.0387
MVK|24347|ES	-2.06713	0.002958	1.18E-05	0.739401	0.038721
CHI3L2|4146|AP	2.067005	8.675672	1.118383	67.30008	0.038734
METTL23|43633|ES	-2.06694	6.45E-05	6.86E-09	0.606912	0.038739
MRPL55|10069|ES	-2.06681	6.94E-05	7.91E-09	0.60958	0.038752
FN1|57392|AA	-2.06662	4.56E-06	3.92E-11	0.530094	0.03877
CABIN1|61390|AD	-2.06652	0.000591	5.13E-07	0.681618	0.038779
FCF1|28425|AD	-2.06644	0.00012	2.28E-08	0.627945	0.038787
ALG10|21066|AD	-2.06625	0.004634	2.83E-05	0.758472	0.038805
NPAS3|27142|AT	-2.06555	0.00029	1.27E-07	0.659384	0.038871
SETMAR|62996|AA	2.065316	121.9181	1.277645	11633.92	0.038893
DKFZP761J1410|47687|A	2.064966	295.5048	1.335445	65388.75	0.038926
ACTN1|28118|ES	-2.0649	1.38E-05	3.37E-10	0.566304	0.038932
KIAA1598|13243|ES	-2.06481	0.000468	3.23E-07	0.677521	0.038941
ANKS3|33838|ES	2.06473	18.81045	1.16055	304.884	0.038949
IL1RAP|68106|AT	2.063687	547.9616	1.372941	218699.8	0.039047
HNRNPUL1|50034|AA	-2.06334	0.000524	4.01E-07	0.684941	0.03908
GRAMD1A|49013|ES	-2.06324	4.45E-15	1.04E-28	0.191265	0.03909
ZSCAN32|33556|ES	-2.06288	0.050905	0.003006	0.86195	0.039124

SLC39A9|28148|ES	-2.06257	8.44E-06	1.27E-10	0.559245	0.039154
MRPL35|54418|RI	2.062232	1.28E+11	3.554719	4.60E+21	0.039186
LINS|32734|AT	-2.06207	0.002422	7.90E-06	0.742112	0.039201
LINS|32733|AT	2.062072	412.9245	1.347505	126535	0.039201
VWA9|31210|ES	2.061761	44.27168	1.205801	1625.461	0.03923
C7orf31|79030|AD	2.060856	1199.648	1.414946	1017109	0.039317
BCL2L13|60990|ES	-2.06002	4.34E-08	4.30E-15	0.438931	0.039396
NKIRAS1|63722|AA	2.059977	2175385	2.030916	2.33E+12	0.039401
GTF2H3|25106|ES	-2.0599	1.85E-10	1.01E-19	0.337131	0.039408
ZNF101|48677|RI	2.05942	589.6592	1.360822	255505.9	0.039454
EFCAB11|28801|AA	-2.05917	1.23E-07	3.25E-14	0.464583	0.039478
C11orf54|18325|AA	-2.05875	0.003212	1.36E-05	0.759217	0.039518
CREM|11231|AP	-2.05818	0.004478	2.60E-05	0.772514	0.039573
ZNF587B|52345|AP	-2.05769	0.023276	0.000648	0.836446	0.03962
ZNF200|33465|AD	2.057491	197838.8	1.78259	2.2E+10	0.039639
SMARCD1|21641|AP	-2.05733	3.30E-05	1.78E-09	0.613674	0.039655
MRPS17|79761|AP	-2.05729	3.58E-05	2.08E-09	0.616073	0.039658
BLOC1S1|22229|AP	2.057082	7.21E+22	11.99933	4.33E+44	0.039678
GSTM2|4061|AT	-2.05661	5.19E-09	6.60E-17	0.408021	0.039724
TAZ|90586|ES	-2.05656	0.000664	6.21E-07	0.709127	0.039728
CIZ1|87714|RI	-2.05625	4.40E-10	5.31E-19	0.364654	0.039759
ZNF302|48994|AD	2.05612	13.83731	1.130738	169.3328	0.039771
PARP10|85520|AD	-2.05595	6.26E-10	1.05E-18	0.371786	0.039787
DISP1|9903|AT	-2.05544	4.73E-12	7.52E-23	0.297803	0.039836
CNTROB|39079|AA	2.053711	308.8483	1.299124	73424.29	0.040004
CENPN|37745|ES	-2.05353	0.000172	4.41E-08	0.673783	0.040022
WDR11|13290|ES	2.053191	6.39E+22	10.85168	3.76E+44	0.040054
C2orf74|53688|AP	-2.05254	2.35E-06	9.93E-12	0.557366	0.040117
LETMD1|21761|ES	2.052536	2143.54	1.413313	3251060	0.040118
SMIM7|48189|AD	2.052189	573.7503	1.330383	247439.5	0.040151
KLHL21|478|AP	-2.05142	1.65E-08	6.03E-16	0.449776	0.040226
ICOSLG|60809|RI	-2.05116	2.71E-05	1.17E-09	0.626557	0.040251
CEP70|66998|AT	2.051061	2741.898	1.421348	5289349	0.040261
C14orf159|28860|ES	2.050664	205.8824	1.265698	33489.49	0.0403
ZNF385A|22176|AP	-2.05025	0.00732	6.65E-05	0.805314	0.040341
TPM1|30987|AD	-2.05013	0.060398	0.004127	0.883872	0.040352
PLA2G6|62210|ES	-2.04804	5.03E-06	4.28E-11	0.591764	0.040556
GJA9|1857|AT	2.047531	224.999	1.260654	40157.35	0.040606
ZNF280B|61274|RI	-2.04744	0.005559	3.86E-05	0.801049	0.040615
DNAJC28|60425|AD	2.047319	9.241979	1.09953	77.68245	0.040627
KIAA0226L|25828|AP	2.046809	16.63168	1.126688	245.5097	0.040677
GIPC1|47980|ES	2.046538	126856.9	1.643935	9.79E+09	0.040703
TFPT|51789|AA	-2.04558	2.20E-14	1.80E-27	0.268124	0.040797
SYT9|14192|AT	-2.04555	0.03568	0.001464	0.869823	0.0408
IYD|78144|ES	2.044718	47857.28	1.563091	1.47E+09	0.040883
ZNF235|50305|AT	2.044615	2728.758	1.387575	5366286	0.040893
GIT2|24381|ES	-2.04458	0.015705	0.000293	0.84206	0.040896
SIDT2|18892|ES	2.044046	11215.86	1.467544	85718350	0.040949
CDC42SE1|7556|ES	-2.04403	0.000714	6.86E-07	0.742323	0.040951
BBS9|79220|ES	-2.04381	2.69E-11	1.96E-21	0.368424	0.040972
WDR52|66200|AT	-2.04303	0.007163	6.27E-05	0.818072	0.04105
WDR52|66199|AT	2.043025	139.6185	1.222375	15947.09	0.04105
ANKRD17|69480|AP	2.042672	41.9466	1.16333	1512.483	0.041085
ZBTB44|19492|RI	2.04258	217781.5	1.644014	2.88E+10	0.041094
MIB2|192|ES	-2.04246	1.08E-18	6.24E-36	0.188082	0.041106
LRRC2|64455|AP	-2.04215	0.049194	0.002732	0.885839	0.041136
TCEA1|83859|ES	-2.04215	1.49E-10	5.50E-20	0.402243	0.041137
HPS1|12762|ES	2.041885	562579.7	1.700975	1.86E+11	0.041163

C11orf48|16388|RI	-2.04166	0.005459	3.67E-05	0.811803	0.041185
PLEC|85512|AP	-2.04114	0.00016	3.64E-08	0.706435	0.041236
ALKBH6|49326|RI	2.040446	21.3427	1.128315	403.7091	0.041306
FOXRED1|19378|ES	2.040237	18336082	1.930965	1.74E+14	0.041327
PARL|67810|AD	-2.04003	2.15E-05	7.02E-10	0.655803	0.041347
IDH1|57208|AP	2.039463	1161607	1.723533	7.83E+11	0.041404
IL4R|35690|ES	-2.03933	0.00096	1.21E-06	0.763059	0.041418
ACAD8|19559|ES	2.038852	2994.379	1.363033	6578205	0.041465
PNPLA6|47109|AP	2.038755	2377.854	1.350451	4186893	0.041474
IRF3|51011|ES	-2.03864	0.021088	0.000516	0.861627	0.041486
ARHGEF1|50100|ES	-2.03805	0.000185	4.77E-08	0.719461	0.041545
EPN2|39700|ES	2.037712	61475.14	1.52304	2.48E+09	0.041579
TRMT61B|53080|ES	-2.0376	7.29E-07	9.11E-13	0.583688	0.04159
PRMT1|51044|ES	2.037514	13527.97	1.436278	1.27E+08	0.041599
MLLT4|78458|AA	2.037228	139.3908	1.205932	16111.86	0.041627
FICD|24213|AA	2.03681	509.7199	1.265157	205361.3	0.041669
LRRC2|64456|AP	2.036231	20.33671	1.119441	369.4538	0.041727
ERCC1|50447|AD	-2.03622	0.003874	1.85E-05	0.812225	0.041728
DNAH9|39293|AT	2.035803	50.53443	1.157345	2206.54	0.04177
ARL16|44152|AD	-2.03564	2.73E-06	1.20E-11	0.621084	0.041786
DUSP15|58908|AT	-2.03464	2.43E-06	9.49E-12	0.622196	0.041887
UCKL1|60177|AP	-2.03441	2.03E-06	6.66E-12	0.619003	0.04191
CHD7|83952|ES	-2.03414	7.04E-05	7.03E-09	0.705656	0.041938
MLLT4|78446|AT	-2.03386	8.95E-11	1.86E-20	0.431443	0.041966
SUPT20H|25661|ES	2.033682	330.3412	1.23398	88433.59	0.041984
RPLP0|24736|AD	-2.03356	0.010656	0.000134	0.848438	0.041996
PTK2B|83150|AP	-2.03341	0.06864	0.00519	0.907769	0.042011
TYMP|96247|ES	-2.03322	0.003517	1.52E-05	0.815804	0.04203
AKAP8L|48081|RI	2.033004	1980555	1.68355	2.33E+12	0.042052
RTN4|53592|ES	2.032273	7.559136	1.074623	53.17262	0.042126
CIC|50146|AA	2.032179	75540.57	1.490565	3.83E+09	0.042135
STRADA|42976|ES	-2.03138	0.032296	0.001177	0.886317	0.042217
RREB1|75249|AP	2.03038	7.34E+08	2.029862	2.65E+17	0.042318
C3orf18|65065|AD	2.030217	1.24E+09	2.063685	7.43E+17	0.042334
GEMIN7|50399|AD	2.029857	22.0775	1.112438	438.1514	0.042371
ELMO2|59684|ES	-2.02898	0.010376	0.000126	0.856085	0.04246
MBD1|45507|AA	-2.02869	0.00243	7.24E-06	0.815512	0.04249
DHPS|47837|AD	-2.02805	2.69E-14	2.07E-27	0.350309	0.042556
CD2BP2|36096|AP	-2.02685	5.79E-07	5.38E-13	0.622547	0.042678
ACACB|24283|ES	2.026444	86.01344	1.157353	6392.444	0.042719
MEN1|16706|AD	-2.02603	0.040886	0.001855	0.900996	0.042761
OAZ3|7653|AP	-2.0253	2.45E-06	9.08E-12	0.659148	0.042837
PHF11|25893|ES	2.024909	93664055	1.80166	4.87E+15	0.042877
NEK1|71147|ES	2.024735	24.15555	1.107242	526.9765	0.042895
GGCT|79138|ES	-2.02462	6.64E-05	5.99E-09	0.735489	0.042906
RANBP3|46996|ES	2.02401	17.26751	1.094333	272.4645	0.042969
TULP3|19733|RI	-2.02395	1.57E-10	5.02E-20	0.489806	0.042975
C8orf31|85393|ES	-2.02367	0.000471	2.83E-07	0.785745	0.043004
LAT2|80064|AT	-2.02354	0.004438	2.34E-05	0.843486	0.043017
LAT2|80065|AT	2.023542	225.3026	1.185556	42816.43	0.043017
SHC4|30508|AP	-2.02334	0.027544	0.000849	0.893594	0.043038
GPR137|16617|AP	2.023202	1858.939	1.265277	2731145	0.043052
MPV17|52966|AT	-2.02317	4.76E-16	6.81E-31	0.332133	0.043056
MUS81|16927|AP	-2.02259	2.01E-05	5.63E-10	0.715398	0.043116
SH2B1|35878|AD	-2.02258	1.18E-06	2.13E-12	0.655399	0.043117
MUS81|16926|AP	2.022468	49820.53	1.396926	1.78E+09	0.043128
RAB40C|32913|ES	2.022008	32.14427	1.112357	928.8867	0.043176
FAM179B|27390|ES	-2.02172	6.53E-08	7.07E-15	0.603264	0.043205

ARL6IP4|25029|AD	-2.02147	0.00025	8.07E-08	0.776999	0.043231
APOL3|61991|RI	2.020829	33.49155	1.11155	1009.117	0.043298
MRPL35|54419|RI	2.020427	17506.58	1.339619	2.29E+08	0.043339
ARHGEF10|82561|AA	-2.01997	0.001177	1.69E-06	0.81845	0.043387
LFNG|78616|AP	-2.01983	0.000374	1.77E-07	0.791461	0.043401
MOV10|4226|AA	2.019795	29.7566	1.105736	800.7839	0.043405
SETMAR|63000|ES	-2.01942	0.048416	0.002563	0.914703	0.043443
SUOX|22342|ES	2.01914	16.95544	1.086496	264.6002	0.043473
APTX|86070|AP	2.019027	2285.028	1.253887	4164131	0.043484
COX20|10474|ES	2.01884	3215.929	1.265567	8171994	0.043504
MANBAL|59344|ES	-2.01868	0.000276	9.70E-08	0.787945	0.04352
SULF2|59728|ES	2.018646	209.8256	1.168145	37689.49	0.043524
FKTN|87133|RI	-2.01811	2.42E-14	1.44E-27	0.405187	0.043579
ZSWIM7|39398|ES	2.017842	3412.025	1.262812	9219041	0.043608
MAP2K3|39818|ES	-2.01747	7.26E-07	7.89E-13	0.668358	0.043646
RAD51D|40267|ES	-2.01743	0.040097	0.001762	0.912448	0.04365
RNH1|13662|AP	2.01648	186.9815	1.157901	30194.34	0.04375
ETV7|75974|ES	-2.01625	0.007617	6.65E-05	0.872703	0.043774
ENOSF1|44476|AD	2.016094	27.94699	1.097154	711.8733	0.04379
MFF|57809|ES	-2.016	3.11E-07	1.47E-13	0.659396	0.0438
PLEKHA5|20644|AP	-2.01568	0.000207	5.42E-08	0.790979	0.043833
IFFO1|19883|ES	-2.0152	0.003854	1.73E-05	0.858679	0.043884
TGFBR1|87053|ES	-2.0148	0.001667	3.31E-06	0.840201	0.043925
VAMP7|90683|ES	-2.01472	2.48E-06	8.72E-12	0.704105	0.043934
RUVBL2|50865|ES	-2.01448	0.012633	0.00018	0.888432	0.043959
FAM3A|90630|AA	-2.01429	1.07E-07	1.78E-14	0.648683	0.043979
ZNF180|50335|ES	-2.01393	0.036282	0.001439	0.914971	0.044017
PDHA1|88629|AP	-2.01345	1.53E-05	3.16E-10	0.744934	0.044067
ITSN1|60468|AT	-2.01335	9.86E-08	1.49E-14	0.651961	0.044078
NBPF10|4448|ES	-2.01313	0.063704	0.004364	0.929868	0.044101
SLC47A1|39745|ES	-2.01301	1.51E-05	3.06E-10	0.746394	0.044113
PIK3R1|72291|AP	-2.01235	0.105071	0.011707	0.943035	0.044183
SEC22A|66465|AA	-2.01231	3.86E-16	3.74E-31	0.397215	0.044187
PTK2B|83149|AP	2.012065	12.61966	1.067852	149.1367	0.044213
JMJD7|30119|AA	2.011975	39.44707	1.09966	1415.048	0.044223
SEC24C|12178|ES	-2.01162	3.11E-06	1.34E-11	0.722067	0.04426
COL8A1|65802|ES	2.011107	80.96232	1.118221	5861.896	0.044314
GEMIN2|27354|ES	-2.01063	6.13E-05	4.79E-09	0.783148	0.044365
PCDHA2|73769|AT	2.010319	16.45305	1.072666	252.3643	0.044397
SF3A1|61729|ES	2.009824	1.01E+19	2.961528	3.48E+37	0.04445
RWDD3|3828|ES	-2.00948	0.010783	0.00013	0.894389	0.044486
TMEM234|1575|ES	2.009124	831.1905	1.178802	586084.3	0.044524
FOXP1|65601|AT	-2.00906	2.05E-12	8.09E-24	0.518058	0.044531
SNRNP200|54521|ES	2.006531	14430.06	1.248897	1.67E+08	0.0448
TNFRSF10C|83064|AT	-2.00639	0.000723	6.17E-07	0.84589	0.044814
SLC34A2|68967|AP	-2.0051	0.002682	8.22E-06	0.875214	0.044953
ANKRD10|26270|AT	-2.00506	0.00069	5.61E-07	0.848989	0.044957
ANKRD10|26271|AT	2.005046	1449.484	1.177817	1783811	0.044958
ATPIF1|1381|RI	2.004721	1116.065	1.169611	1064969	0.044993
MINA|65746|AD	2.004534	198.3109	1.124813	34963.34	0.045013
TTLL4|57561|AA	-2.00329	6.14E-06	4.89E-11	0.771404	0.045146
NECAB3|59008|AA	-2.00319	7.87E-06	7.98E-11	0.776	0.045157
HNRNPK|86708|AP	-2.00308	2.59E-05	8.39E-10	0.796632	0.045169
HNRNPK|86709|AP	2.003077	38682.37	1.255278	1.19E+09	0.045169
GSDMD|85422|AP	2.002482	35008.65	1.248776	9.81E+08	0.045233
MRPL51|19868|AP	2.00232	1.76E+11	1.729234	1.78E+22	0.04525
MRPL51|19869|AP	-2.00228	5.70E-12	5.62E-23	0.57858	0.045255
TBC1D3|40560|AP	2.001664	46017.19	1.250666	1.69E+09	0.045321

FARP1|26163|AP	2.001508	124.6613	1.10535	14059.29	0.045338
POLM|79464|AD	-2.00099	0.008808	8.55E-05	0.907526	0.045393
CBWD2|55055|ES	2.000755	657.2802	1.141427	378488.9	0.045419
ZNF208|48802|ES	2.000681	28.75031	1.070743	771.9689	0.045427
LEKR1|67370|AT	1.999864	32.73474	1.072079	999.5191	0.045515
FHL2|54823|AT	-1.99985	9.34E-07	1.15E-12	0.758117	0.045516
TPGS2|45266|AT	1.999716	2.45E+08	1.468167	4.1E+16	0.045531
AGO3|1740|AT	1.998483	545.243	1.129134	263290.1	0.045664
FBXO10|86409|AT	-1.99848	3.52E-07	1.65E-13	0.750969	0.045665
FBXO10|86408|AT	1.998444	2843021	1.331271	6.07E+12	0.045669
ZNF177|47304|AA	-1.99797	0.016695	0.000301	0.925102	0.04572
SNAPC3|85915|RI	-1.99756	7.94E-09	8.96E-17	0.703985	0.045765
PDCD5|48877|AP	-1.99739	2.02E-05	4.99E-10	0.816632	0.045783
PDCD5|48878|AP	1.997382	49557.14	1.224493	2.01E+09	0.045784
DNAJC14|22262|AP	-1.99711	0.009278	9.39E-05	0.916639	0.045814
DNAJC14|22260|AP	1.996853	107.7252	1.090294	10643.65	0.045841
CNOT2|23366|AT	1.996102	8765.748	1.178641	65192347	0.045923
CITED1|89454|ES	-1.99592	0.010253	0.000114	0.920801	0.045943
EFCAB13|99855|ES	1.995781	30.00085	1.062941	846.7551	0.045958
PSPH|79781|AA	1.995435	541.2363	1.118379	261929.7	0.045995
MED14|98321|ES	1.995406	7.43E+11	1.624995	3.40E+23	0.045999
GBA|8041|AP	1.99501	109.0622	1.085915	10953.49	0.046042
GBA|8040|AP	-1.995	0.009169	9.13E-05	0.920913	0.046043
BACH1|60323|AP	-1.99479	0.036008	0.001374	0.943625	0.046066
MAGOHB|20472|ES	1.994785	762.5374	1.122826	517857.2	0.046066
SGSM2|38394|ES	1.994148	188.0364	1.093919	32322.05	0.046136
SFMBT2|10709|AT	1.994125	4957531	1.302255	1.89E+13	0.046138
VWA9|31215|ES	1.993745	69.6	1.074533	4508.152	0.04618
SARDH|88080|AT	1.99364	38.38156	1.063551	1385.119	0.046191
HNRNPF|11322|AP	1.993563	39945.92	1.195506	1.33E+09	0.0462
TMEM8B|86319|AP	1.99347	1703.46	1.133214	2560660	0.04621
RANGRF|39168|RI	1.992749	13261.48	1.169031	1.5E+08	0.046289
UBAP1|86154|ES	-1.99271	0.122416	0.015512	0.966071	0.046293
POC1A|65180|ES	-1.9927	0.007546	6.17E-05	0.922854	0.046294
DNAH7|56630|AT	1.992581	238.2615	1.093732	51903.55	0.046307
ELOVL5|76490|AP	-1.99252	1.62E-05	3.16E-10	0.835111	0.046314
NCOR1|39419|AD	-1.99215	7.34E-08	7.03E-15	0.766902	0.046355
PTPN18|55344|ES	1.992017	102.0338	1.077264	9664.212	0.046369
MACF1|1881|ES	-1.99086	0.003128	1.07E-05	0.914373	0.046496
FAM213B|293|AA	1.990601	7258.637	1.146625	45950333	0.046525
NDUFB8|12824|AT	-1.99009	1.89E-15	5.97E-30	0.598618	0.046582
IDUA|68443|ES	-1.99008	3.44E-09	1.59E-17	0.744602	0.046582
NDUFA7|47216|AT	1.989993	158.442	1.079434	23256.5	0.046592
USP19|64839|AD	-1.98998	1.18E-06	1.71E-12	0.813933	0.046593
BCAT2|50811|AP	1.989797	5605.922	1.138159	27611581	0.046613
HKR1|49495|ES	-1.98976	0.063313	0.004178	0.959519	0.046618
BCAT2|50810|AP	-1.98962	0.000178	3.62E-08	0.87929	0.046633
RBBP4|1626|ES	-1.98953	0.198207	0.040243	0.976232	0.046642
PRICKLE4|76141|AA	-1.98904	0.076138	0.006019	0.963054	0.046697
PDHB|65453|AD	1.988848	5712.499	1.133863	28780053	0.046718
NFE2L1|42158|AP	1.987923	84.6869	1.064422	6737.808	0.04682
USMG5|13004|ES	1.987806	2.13E+12	1.488281	3.05E+24	0.046833
ATXN3|28978|ES	-1.98778	1.03E-05	1.26E-10	0.851605	0.046836
THTPA|26760|RI	1.987719	1757.421	1.109963	2782552	0.046843
PGS1|43878|ES	-1.98764	1.03E-10	1.45E-20	0.725954	0.046851
SRP54|27180|ES	1.987236	8.89E+20	1.938652	4.08E+41	0.046896
SFTA3|27254|ES	1.987234	47.32087	1.054352	2123.829	0.046897
KMT2C|82413|AP	-1.98696	0.022537	0.000535	0.949786	0.046927

BCCIP|13433|AT	-1.98686	0.000683	5.15E-07	0.906044	0.046938
KMT2C|82412|AP	1.986853	44.35784	1.052662	1869.183	0.046939
TMEM108|66830|AA	1.986168	70.42158	1.057736	4688.502	0.047015
PCDH11X|89581|AT	-1.98591	0.014596	0.000225	0.946272	0.047043
ZNF227|50299|AA	-1.9855	0.001195	1.56E-06	0.917098	0.047089
RIC8A|13587|AA	1.984911	4.58E+10	1.361411	1.54E+21	0.047154
EYA3|1370|ES	-1.98442	0.000872	8.28E-07	0.91684	0.047209
STEAP4|80362|AT	1.98379	85.1564	1.054829	6874.68	0.047279
STEAP4|80361|AT	-1.98379	0.011743	0.000145	0.948028	0.04728
STK33|14240|AP	-1.98362	3.69E-05	1.54E-09	0.885363	0.047298
DNAJA4|32073|AT	-1.98349	1.08E-10	1.54E-20	0.761766	0.047313
GGA1|62120|AP	1.983054	1055.952	1.08444	1028212	0.047361
CYP51A1|80416|AP	1.983049	50.56249	1.046729	2442.434	0.047362
SYS1|59527|AT	-1.98298	1.37E-18	3.04E-36	0.620359	0.047369
GGA1|62121|AP	-1.98266	0.000948	9.73E-07	0.923411	0.047406
NAP1L1|23493|ES	-1.9826	0.067536	0.004704	0.969695	0.047412
PACRGL|68908|ES	1.982339	52955.33	1.130627	2.48E+09	0.047441
SNAP23|30166|AP	-1.9823	4.75E-11	2.95E-21	0.765029	0.047446
HYI|2182|AT	-1.98224	1.33E-05	2.01E-10	0.881458	0.047452
ZNF567|49414|AP	1.981451	6.729762	1.02089	44.36295	0.047541
FARP1|26162|AP	-1.98122	0.008273	7.21E-05	0.949856	0.047566
TRIM9|27503|AT	-1.98116	0.038762	0.001556	0.965827	0.047574
TSGA10|54665|AA	-1.98091	0.035436	0.001301	0.9653	0.047601
ZNF175|51359|AT	-1.9804	0.002066	4.55E-06	0.938202	0.047659
REPIN1|82247|ES	1.979669	17.15127	1.028693	285.9609	0.047741
TBC1D9B|74991|ES	1.979348	1.41E+08	1.201766	1.66E+16	0.047777
MLH1|63953|AD	1.978797	170.3865	1.050117	27646.01	0.047839
STRA13|44263|AD	1.978661	3.74E+08	1.205057	1.16E+17	0.047854
C14orf159|28870|ES	-1.97865	0.002751	8.00E-06	0.945839	0.047855
MAPK10|69824|AA	1.977747	120.2226	1.044005	13844.25	0.047957
DNAH7|56629|AT	-1.97739	0.004297	1.94E-05	0.953117	0.047998
CKLF|36731|ES	-1.9771	8.37E-09	8.23E-17	0.8511	0.04803
METTL23|43648|AD	1.975998	12.87082	1.020948	162.259	0.048155
TGFBR3|3739|AP	1.975463	16.64992	1.022311	271.1697	0.048216
OS9|22697|AA	1.974942	276820.4	1.099696	6.97E+10	0.048275
ZNF669|10513|AD	1.974803	56.88817	1.030831	3139.472	0.048291
EIF3M|14855|ES	-1.97434	0.00223	5.20E-06	0.956528	0.048344
STXBP6|27043|AD	1.972921	559.0642	1.042421	299833.4	0.048505
SZT2|2175|AT	1.972176	1.7E+08	1.124526	2.58E+16	0.04859
CNTN6|62939|AT	-1.97157	2.41E-05	6.16E-10	0.939297	0.048658
RFC4|68062|ES	-1.97089	2.59E-11	7.70E-22	0.873649	0.048737
CADM1|18849|ES	-1.97053	0.001042	1.13E-06	0.963861	0.048778
NAT16|81045|AT	1.970348	47.13835	1.020515	2177.357	0.048798
EXOSC10|645|RI	1.970177	90.80118	1.023647	8054.392	0.048818
EGLN3|27148|AT	1.969972	3982.975	1.043014	15209848	0.048842
CNTRL|87411|AT	-1.96972	2.40E-08	6.26E-16	0.916731	0.04887
EGLN3|27149|AT	-1.96963	0.000251	6.58E-08	0.960156	0.048881
GFM2|72503|ES	-1.96952	5.16E-05	2.79E-09	0.953221	0.048893
MTFR1L|1197|AP	1.969514	166326.5	1.060022	2.61E+10	0.048894
PPFIA3|50895|RI	-1.96914	0.006323	4.09E-05	0.976678	0.048937
GMEB2|60137|AP	-1.96908	0.059049	0.003533	0.986993	0.048944
ZNF789|80652|ES	1.968763	37.15284	1.016288	1358.211	0.04898
SYVN1|16795|AA	1.968346	675.2789	1.028133	443523.9	0.049028
SUV420H1|17300|ES	-1.96808	7.10E-07	5.34E-13	0.943316	0.049059
ZDHHC4|78749|ES	1.967413	10.14268	1.008811	101.9754	0.049136
ERBB2IP|72267|ES	-1.9669	0.017844	0.000323	0.98591	0.049195
LARGE|61939|AP	-1.96685	0.127566	0.016391	0.992813	0.0492
PPHLN1|21222|ES	-1.96682	0.036522	0.001349	0.988535	0.049204

U2AF1|60778|ES	1.966403	1079.03	1.023131	1137983	0.049252
AKAP6|27135|AT	1.966381	4185.793	1.027588	17050479	0.049255
AKAP6|27136|AT	-1.96636	0.000239	5.86E-08	0.973244	0.049257
CAPN10|58269|AT	-1.9663	2.53E-08	6.77E-16	0.945191	0.049264
C1D|53822|ES	1.96551	557.398	1.018001	305198.7	0.049355
LIPA|12487|AD	1.965277	2747.952	1.021636	7391320	0.049382
COPZ1|22171|ES	-1.96523	0.016792	0.000285	0.989118	0.049388
FAM213A|12362|AP	-1.96457	0.010911	0.00012	0.989471	0.049464
EPN1|52137|AP	1.963492	962.5072	1.012419	915055.9	0.049589
EPN1|52136|AP	-1.96349	0.001039	1.09E-06	0.987748	0.04959
SHC4|30507|AP	1.963052	33.0346	1.005518	1085.296	0.04964
ZNF446|52471|AD	1.962603	230.7578	1.007343	52861	0.049692
ZMAT5|61664|ES	-1.96255	0.000629	4.00E-07	0.990325	0.049698
ENOSF1|44465|ES	-1.9613	8.97E-07	8.12E-13	0.990572	0.049844
OPTN|215727|ES	1.961281	9.85E+17	1.028222	9.44E+35	0.049846
EFCAB13|42070|AT	1.961184	79.36628	1.002724	6281.893	0.049858
C11orf74|15440|ES	1.960911	50452.02	1.005241	2.53E+09	0.049889
MRPL36|71494|AD	1.960909	3405.555	1.003926	11552455	0.04989
UBXN11|101231|ES	1.96081	188.4736	1.002262	35442.12	0.049901
SERPINB6|75146|AP	-1.96062	0.000171	2.93E-08	0.997081	0.049923
COL14A1|85018|AT	-1.96034	0.001153	1.33E-06	0.998713	0.049956
UBXN11|1254|ES	1.960061	136.3873	1.000243	18596.96	0.049989
