# Supplementary material for: Alternative splicing events implicated in carcinogenesis and prognosis of thyroid gland cancer
Source: Sci Rep. 2021 Mar 1;11:4841. doi: 10.1038/s41598-021-84403-6 (PMC7921437; doi:10.1038/s41598-021-84403-6)
Supplement: Supplementary file 3 — Supplementary Information 3. [file 41598_2021_84403_MOESM3_ESM.pdf]

SF	AS	cor	pvalue	Regulation
HSPB1	CABIN1-61386-AP	0.603480679	4.88E-50	postive
HSPB1	RARA-40856-AP	0.672332229	7.15E-66	postive
ZC3H11A	KIAA2013-694-AT	-0.6003872	2.05E-49	negative
NOSIP	CABIN1-61386-AP	0.650386806	2.24E-60	postive
NOSIP	CREBZF-18139-RI	-0.612260621	7.67E-52	negative
NOSIP	HAS3-37253-AT	-0.602187974	8.90E-50	negative
NOSIP	HAS3-37254-AT	0.602190695	8.89E-50	postive
NOSIP	RARA-40856-AP	0.697930364	6.51E-73	postive
SNRPB	CABIN1-61386-AP	0.636095182	4.90E-57	postive
SNRPF	RARA-40856-AP	0.639168339	9.70E-58	postive
SNRPF	EMP1-20539-ES	-0.691481848	4.54E-71	negative
WDR83	RARA-40856-AP	0.638101289	1.71E-57	postive
ZNF346	PCNA-58649-AP	-0.620666092	1.27E-53	negative
ZNF346	PCNA-58648-AP	0.620662181	1.27E-53	postive
ZNF346	LGALS3-27617-AT	-0.610771929	1.56E-51	negative
ZNF346	IL1RAP-68106-AT	0.703842116	1.20E-74	postive
THOC6	CABIN1-61386-AP	0.611130818	1.32E-51	postive
THOC6	RARA-40856-AP	0.612396257	7.18E-52	postive
FAM50A	NUPL2-78961-AT	0.615747358	1.42E-52	postive
FAM50A	NUPL2-78960-AT	-0.615750583	1.42E-52	negative
FAM50A	RARA-40856-AP	0.648784182	5.41E-60	postive
CLK1	DYNLT1-78273-AP	0.637690348	2.12E-57	postive
CLK1	DYNLT1-78274-AP	-0.637690348	2.12E-57	negative
CLK1	BCAT2-50816-ES	0.664077378	9.47E-64	postive
CLK1	EPOR-47693-ES	0.612906576	5.62E-52	postive
CLK1	EXOSC10-645-RI	0.706558302	1.86E-75	postive
ACIN1	DAPK2-31076-ES	0.610592631	1.70E-51	postive
ACIN1	NDUFB8-12824-AT	-0.664939932	5.72E-64	negative
ACIN1	EXOSC10-645-RI	0.603441555	4.97E-50	postive
TIA1	DYNLT1-78273-AP	0.699178655	2.83E-73	postive
TIA1	DYNLT1-78274-AP	-0.699178655	2.83E-73	negative
TIA1	DAPK2-31076-ES	0.66816928	8.57E-65	postive
TIA1	BCAT2-50816-ES	0.657850288	3.41E-62	postive
TIA1	EPOR-47693-ES	0.620028489	1.74E-53	postive
TIA1	NDUFB8-12824-AT	-0.615923506	1.30E-52	negative
TIA1	EXOSC10-645-RI	0.736492672	4.71E-85	postive
FUBP1	C2orf68-54308-AT	-0.644429961	5.80E-59	negative
FUBP1	C2orf68-54309-AT	0.644426377	5.81E-59	postive
FUBP1	MPV17-52966-AT	-0.626462224	6.98E-55	negative
MAGOH	RARA-40856-AP	0.633222617	2.19E-56	postive
DDX3X	HAS3-37253-AT	0.620920564	1.12E-53	postive
DDX3X	HAS3-37254-AT	-0.620919556	1.12E-53	negative
DDX3X	WDR20-29338-AT	0.624093098	2.30E-54	postive
SRSF11	BOP1-85554-RI	0.604476086	3.07E-50	postive
SRSF11	TPM1-30985-AT	-0.601904245	1.02E-49	negative
SRSF11	C5orf45-74948-RI	0.612018109	8.61E-52	postive
SRSF11	DYNLT1-78273-AP	0.641393422	2.97E-58	postive
SRSF11	DYNLT1-78274-AP	-0.641393422	2.97E-58	negative
SRSF11	PRICKLE3-89098-AT	0.60160938	1.16E-49	postive
SRSF11	PRICKLE3-89099-AT	-0.601628574	1.15E-49	negative
SRSF11	DAPK2-31076-ES	0.670411815	2.26E-65	postive
SRSF11	BCAT2-50816-ES	0.622157457	6.05E-54	postive
SRSF11	NDUFB8-12824-AT	-0.656993743	5.54E-62	negative
SRSF11	EXOSC10-645-RI	0.721430403	4.56E-80	postive
DDX17	MPV17-52966-AT	-0.603782511	4.24E-50	negative
PPWD1	NDUFB8-12824-AT	-0.631192154	6.25E-56	negative
LUC7L	STXBP2-47123-AP	0.640142083	5.78E-58	postive

LUC7L	STXBP2-47122-AP	-0.64014397	5.78E-58	negative
LUC7L	ZNF331-51722-AP	0.692274656	2.71E-71	postive
LUC7L	VCL-12254-ES	0.609443393	2.95E-51	postive
LUC7L	DYNLT1-78273-AP	0.694687079	5.58E-72	postive
LUC7L	DYNLT1-78274-AP	-0.694687079	5.58E-72	negative
LUC7L	DAPK2-31076-ES	0.613837018	3.59E-52	postive
LUC7L	ANKRD10-26274-ES	0.612669798	6.30E-52	postive
LUC7L	BCAT2-50816-ES	0.702585125	2.84E-74	postive
LUC7L	EPOR-47693-ES	0.662583461	2.25E-63	postive
LUC7L	PDHA1-88629-AP	-0.639728771	7.20E-58	negative
LUC7L	EXOSC10-645-RI	0.739425341	4.59E-86	postive
LUC7L	FAM213A-12362-AP	-0.6658798	3.30E-64	negative
SRRM2	USP4-64850-AT	-0.636716699	3.53E-57	negative
SRRM2	INO80C-45173-AT	-0.68314116	9.33E-69	negative
SRRM2	INO80C-45172-AT	0.68314853	9.28E-69	postive
SRRM2	TTLL5-28518-AT	-0.675064281	1.37E-66	negative
SRRM2	USP4-64851-AT	0.735535962	1.00E-84	postive
SRRM2	TRIM11-10208-AT	-0.602551814	7.52E-50	negative
SRRM2	PTK2B-83155-AT	-0.650775651	1.80E-60	negative
SRRM2	RHBDF1-32789-AT	-0.602058565	9.45E-50	negative
SRRM2	C2orf68-54308-AT	-0.744165867	9.97E-88	negative
SRRM2	C2orf68-54309-AT	0.744162893	1.00E-87	postive
SRRM2	CCDC124-48386-AP	0.605553072	1.85E-50	postive
SRRM2	CCDC124-48385-AP	-0.605551738	1.85E-50	negative
SRRM2	HSP90AA1-29333-AT	-0.659973672	1.01E-62	negative
SRRM2	KCNC3-51173-AT	-0.620366626	1.47E-53	negative
SRRM2	KCNC3-51172-AT	0.620364079	1.47E-53	postive
SRRM2	PET100-47118-AT	-0.627335207	4.48E-55	negative
SRRM2	NACA-22488-AD	0.6438389	7.98E-59	postive
SRRM2	MPV17-52966-AT	-0.817118519	4.61E-119	negative
SRRM2	SNRNP200-54521-ES	0.630831028	7.52E-56	postive
SRRM2	CNOT2-23366-AT	0.678609384	1.56E-67	postive
SRRM2	NDUFB8-12824-AT	-0.690263896	9.99E-71	negative
LENG1	CREBZF-18139-RI	-0.62658748	6.55E-55	negative
LENG1	RARA-40856-AP	0.677223741	3.67E-67	postive
ZFR	NUPL2-78961-AT	-0.605802949	1.65E-50	negative
ZFR	NUPL2-78960-AT	0.605799014	1.65E-50	postive
ZFR	KIAA2013-694-AT	-0.605331726	2.06E-50	negative
PRPF4B	PTCD2-72456-AT	-0.640392988	5.06E-58	negative
PRPF4B	PTCD2-72458-AT	0.640387326	5.07E-58	postive
PRPF4B	C2orf68-54308-AT	-0.655378115	1.38E-61	negative
PRPF4B	C2orf68-54309-AT	0.655378999	1.38E-61	postive
PRPF4B	KIAA2013-694-AT	-0.675546491	1.02E-66	negative
PRPF4B	MPV17-52966-AT	-0.640643678	4.43E-58	negative
PRPF4B	CNOT2-23366-AT	0.62149272	8.43E-54	postive
SF3B1	C2orf68-54308-AT	-0.652305353	7.72E-61	negative
SF3B1	C2orf68-54309-AT	0.652301455	7.73E-61	postive
RNF40	CABIN1-61386-AP	-0.610839053	1.52E-51	negative
RNF40	RARA-40856-AP	-0.608697774	4.20E-51	negative
RNF40	KCNC3-51173-AT	-0.620858343	1.15E-53	negative
RNF40	KCNC3-51172-AT	0.620863467	1.15E-53	postive
LUC7L3	CCDC53-24023-ES	0.606679699	1.09E-50	postive
LUC7L3	DYNLT1-78273-AP	0.648938589	4.97E-60	postive
LUC7L3	DYNLT1-78274-AP	-0.648938589	4.97E-60	negative
LUC7L3	DAPK2-31076-ES	0.621517939	8.32E-54	postive
LUC7L3	BCAT2-50816-ES	0.61205	8.48E-52	postive
LUC7L3	NDUFB8-12824-AT	-0.610457077	1.82E-51	negative
LUC7L3	EXOSC10-645-RI	0.725284853	2.60E-81	postive

PRPF31	RARA-40856-AP	0.610027752	2.23E-51	postive
PNN	USP4-64851-AT	0.633332155	2.07E-56	postive
PNN	PTK2B-83155-AT	-0.601492947	1.23E-49	negative
PNN	HSP90AA1-29333-AT	-0.611202282	1.27E-51	negative
PNN	MPV17-52966-AT	-0.635729329	5.93E-57	negative
PNN	NDUFB8-12824-AT	-0.667612264	1.19E-64	negative
CWC15	ZNF517-85650-AT	-0.605434521	1.96E-50	negative
CWC15	RARA-40856-AP	0.692644246	2.13E-71	postive
CDK10	ZNF331-51722-AP	0.626109742	8.34E-55	postive
SREK1	USP4-64850-AT	-0.66266774	2.15E-63	negative
SREK1	INO80C-45173-AT	-0.629530763	1.47E-55	negative
SREK1	INO80C-45172-AT	0.629537891	1.46E-55	postive
SREK1	TTLL5-28518-AT	-0.62364474	2.88E-54	negative
SREK1	USP4-64851-AT	0.735269019	1.23E-84	postive
SREK1	PTK2B-83155-AT	-0.650971688	1.62E-60	negative
SREK1	CCDC124-48386-AP	0.628944629	1.98E-55	postive
SREK1	CCDC124-48385-AP	-0.62895169	1.97E-55	negative
SREK1	HSP90AA1-29333-AT	-0.60536175	2.03E-50	negative
SREK1	MPV17-52966-AT	-0.646700368	1.69E-59	negative
SREK1	SNRNP200-54521-ES	0.604698055	2.77E-50	postive
SREK1	CNOT2-23366-AT	0.606212594	1.36E-50	postive
SNRNP70	BOP1-85554-RI	0.627088856	5.08E-55	postive
SNRNP70	CCDC53-24023-ES	0.616901888	8.09E-53	postive
SNRNP70	ZNF331-51722-AP	0.646653811	1.74E-59	postive
SNRNP70	DYNLT1-78273-AP	0.646958864	1.47E-59	postive
SNRNP70	DYNLT1-78274-AP	-0.646958864	1.47E-59	negative
SNRNP70	EXOSC10-645-RI	0.640489276	4.81E-58	postive
DDX46	CABIN1-61386-AP	-0.606117064	1.42E-50	negative
DDX46	IFIH1-55768-AT	-0.60545203	1.94E-50	negative
DDX46	IFIH1-55767-AT	0.605434907	1.96E-50	postive
DDX46	PTCD2-72456-AT	-0.704552043	7.41E-75	negative
DDX46	PTCD2-72458-AT	0.704546917	7.43E-75	postive
DDX46	NUPL2-78961-AT	-0.637754205	2.05E-57	negative
DDX46	NUPL2-78960-AT	0.637752342	2.05E-57	postive
DDX46	HAS3-37253-AT	0.638887303	1.13E-57	postive
DDX46	HAS3-37254-AT	-0.638888549	1.12E-57	negative
DDX46	KIAA2013-694-AT	-0.723657787	8.77E-81	negative
DDX46	KCNC3-51173-AT	-0.617137802	7.21E-53	negative
DDX46	KCNC3-51172-AT	0.617145117	7.18E-53	postive
DDX46	F11R-8516-AP	-0.604613918	2.88E-50	negative
DDX46	F11R-8517-AP	0.60461248	2.88E-50	postive
DDX46	ANKS1B-23885-AT	0.604720613	2.74E-50	postive
DDX46	ANKS1B-23886-AT	-0.604716371	2.74E-50	negative
DDX46	SZT2-2175-AT	0.603606368	4.60E-50	postive
RAVER1	TM9SF1-26891-AP	-0.632642278	2.96E-56	negative
RAVER1	TM9SF1-26892-AP	0.63264155	2.96E-56	postive
PAXBP1	TPM1-30985-AT	-0.60333141	5.23E-50	negative
PAXBP1	DYNLT1-78273-AP	0.672239393	7.56E-66	postive
PAXBP1	DYNLT1-78274-AP	-0.672239393	7.56E-66	negative
PAXBP1	DAPK2-31076-ES	0.632949835	2.52E-56	postive
PAXBP1	BCAT2-50816-ES	0.649659751	3.34E-60	postive
PAXBP1	EXOSC10-645-RI	0.724433869	4.92E-81	postive
CCDC12	CABIN1-61386-AP	0.646408112	1.98E-59	postive
CCDC12	PTCD2-72456-AT	0.601141853	1.44E-49	postive
CCDC12	PTCD2-72458-AT	-0.60114582	1.44E-49	negative
CCDC12	CREBZF-18139-RI	-0.631199306	6.22E-56	negative
CCDC12	NUPL2-78961-AT	0.611689803	1.01E-51	postive
CCDC12	NUPL2-78960-AT	-0.611698706	1.00E-51	negative

CCDC12	HAS3-37253-AT	-0.672052201	8.46E-66	negative
CCDC12	HAS3-37254-AT	0.672051068	8.47E-66	postive
CCDC12	DENND1B-9304-AT	0.622841947	4.30E-54	postive
CCDC12	DENND1B-9305-AT	-0.622840332	4.31E-54	negative
CCDC12	RARA-40856-AP	0.766296014	5.22E-96	postive
CCDC12	TGFBR3-3739-AP	-0.603027238	6.03E-50	negative
RBM25	USP4-64850-AT	-0.636142377	4.78E-57	negative
RBM25	INO80C-45173-AT	-0.615848001	1.35E-52	negative
RBM25	INO80C-45172-AT	0.615846042	1.35E-52	postive
RBM25	TTLL5-28518-AT	-0.633175136	2.24E-56	negative
RBM25	USP4-64851-AT	0.683749785	6.36E-69	postive
RBM25	PTK2B-83155-AT	-0.619276242	2.52E-53	negative
RBM25	TPM1-30985-AT	-0.607827884	6.34E-51	negative
RBM25	CCDC124-48386-AP	0.608977065	3.68E-51	postive
RBM25	CCDC124-48385-AP	-0.608983678	3.67E-51	negative
RBM25	HSP90AA1-29333-AT	-0.634126765	1.37E-56	negative
RBM25	C5orf45-74948-RI	0.691431638	4.69E-71	postive
RBM25	PTRH2-42790-AP	-0.617105209	7.32E-53	negative
RBM25	PTRH2-42789-AP	0.617103059	7.33E-53	postive
RBM25	PRICKLE3-89098-AT	0.638955137	1.09E-57	postive
RBM25	PRICKLE3-89099-AT	-0.638966528	1.08E-57	negative
RBM25	DAPK2-31076-ES	0.693473332	1.24E-71	postive
RBM25	NACA-22488-AD	0.625816586	9.67E-55	postive
RBM25	MPV17-52966-AT	-0.686321134	1.25E-69	negative
RBM25	SNRNP200-54521-ES	0.610620759	1.68E-51	postive
RBM25	NDUFB8-12824-AT	-0.705345464	4.29E-75	negative
RBM25	EXOSC10-645-RI	0.648457602	6.47E-60	postive
CLK4	DYNLT1-78273-AP	0.654724443	1.99E-61	postive
CLK4	DYNLT1-78274-AP	-0.654724443	1.99E-61	negative
CLK4	BCAT2-50816-ES	0.617771176	5.29E-53	postive
CLK4	EXOSC10-645-RI	0.711499114	5.90E-77	postive
SNRNP25	IL1RAP-68106-AT	0.626129355	8.26E-55	postive
EIF3A	CABIN1-61386-AP	-0.622887986	4.21E-54	negative
EIF3A	PTCD2-72456-AT	-0.676531152	5.60E-67	negative
EIF3A	PTCD2-72458-AT	0.676531631	5.60E-67	postive
EIF3A	ARHGEF26-67329-AP	-0.635740439	5.90E-57	negative
EIF3A	FADS1-16298-AP	-0.614497788	2.61E-52	negative
EIF3A	NUPL2-78961-AT	-0.637598745	2.22E-57	negative
EIF3A	NUPL2-78960-AT	0.637595676	2.23E-57	postive
EIF3A	HAS3-37253-AT	0.609002919	3.64E-51	postive
EIF3A	HAS3-37254-AT	-0.609005052	3.63E-51	negative
EIF3A	ARHGEF26-67328-AP	0.631748177	4.69E-56	postive
EIF3A	DENND1B-9304-AT	-0.633227261	2.18E-56	negative
EIF3A	DENND1B-9305-AT	0.633224828	2.19E-56	postive
EIF3A	RARA-40856-AP	-0.695526377	3.21E-72	negative
EIF3A	TGFBR3-3739-AP	0.632351117	3.44E-56	postive
EIF3A	SZT2-2175-AT	0.610235897	2.02E-51	postive
SNRPD2	CABIN1-61386-AP	0.605318499	2.07E-50	postive
SNRPD2	HAS3-37253-AT	-0.642574314	1.58E-58	negative
SNRPD2	HAS3-37254-AT	0.642573499	1.58E-58	postive
SNRPD2	RARA-40856-AP	0.709628499	2.20E-76	postive
SNRPD2	TENC1-21923-AP	0.6016974	1.12E-49	postive
PRPF39	BOP1-85554-RI	0.639618128	7.64E-58	postive
PRPF39	DYNLT1-78273-AP	0.629826028	1.26E-55	postive
PRPF39	DYNLT1-78274-AP	-0.629826028	1.26E-55	negative
PRPF39	DAPK2-31076-ES	0.670698045	1.90E-65	postive
PRPF39	EXOSC10-645-RI	0.717611835	7.44E-79	postive
NELFE	RARA-40856-AP	0.612813295	5.88E-52	postive

DDX39B	USP4-64850-AT	-0.619604386	2.15E-53	negative
DDX39B	BOP1-85554-RI	0.62351354	3.08E-54	postive
DDX39B	IDH3G-90494-RI	0.627419757	4.30E-55	postive
DDX39B	RHBDF1-32789-AT	-0.667265237	1.46E-64	negative
DDX39B	CLDND1-65747-AP	0.618405792	3.87E-53	postive
DDX39B	CCDC53-24023-ES	0.623879739	2.56E-54	postive
DDX39B	HSP90AA1-29333-AT	-0.628029094	3.15E-55	negative
DDX39B	VCL-12254-ES	0.612858416	5.75E-52	postive
DDX39B	C5orf45-74948-RI	0.775198858	1.33E-99	postive
DDX39B	PTRH2-42790-AP	-0.635882193	5.48E-57	negative
DDX39B	PTRH2-42789-AP	0.635872898	5.50E-57	postive
DDX39B	DYNLT1-78273-AP	0.70079866	9.51E-74	postive
DDX39B	DYNLT1-78274-AP	-0.70079866	9.51E-74	negative
DDX39B	PRICKLE3-89098-AT	0.664652971	6.77E-64	postive
DDX39B	PRICKLE3-89099-AT	-0.664658988	6.74E-64	negative
DDX39B	DAPK2-31076-ES	0.765753475	8.55E-96	postive
DDX39B	PET100-47118-AT	-0.642284486	1.84E-58	negative
DDX39B	NACA-22488-AD	0.605124759	2.26E-50	postive
DDX39B	BCAT2-50816-ES	0.605393365	2.00E-50	postive
DDX39B	MPV17-52966-AT	-0.681545709	2.53E-68	negative
DDX39B	NDUFB8-12824-AT	-0.803398842	3.53E-112	negative
DDX39B	EXOSC10-645-RI	0.757772442	1.03E-92	postive
UBL5	CABIN1-61386-AP	0.605178853	2.21E-50	postive
UBL5	PTCD2-72456-AT	0.664610169	6.94E-64	postive
UBL5	PTCD2-72458-AT	-0.664611983	6.93E-64	negative
UBL5	ARHGEF26-67329-AP	0.637988927	1.81E-57	postive
UBL5	NUPL2-78961-AT	0.64401474	7.26E-59	postive
UBL5	NUPL2-78960-AT	-0.644015383	7.26E-59	negative
UBL5	ARHGEF26-67328-AP	-0.635657992	6.16E-57	negative
UBL5	DENND1B-9304-AT	0.66262838	2.20E-63	postive
UBL5	DENND1B-9305-AT	-0.662625108	2.20E-63	negative
UBL5	ZNF517-85650-AT	-0.607638507	6.94E-51	negative
UBL5	RARA-40856-AP	0.787972151	4.63E-105	postive
RBM39	DYNLT1-78273-AP	0.607714151	6.70E-51	postive
RBM39	DYNLT1-78274-AP	-0.607714151	6.70E-51	negative
RBM39	NDUFB8-12824-AT	-0.624469854	1.90E-54	negative
RBM39	EXOSC10-645-RI	0.684393244	4.24E-69	postive
CCDC130	ZNF331-51722-AP	0.664014483	9.82E-64	postive
CCDC130	DYNLT1-78273-AP	0.619430335	2.34E-53	postive
CCDC130	DYNLT1-78274-AP	-0.619430335	2.34E-53	negative
CCDC130	EXOSC10-645-RI	0.603951623	3.92E-50	postive
LSM4	PTCD2-72456-AT	0.629175326	1.76E-55	postive
LSM4	PTCD2-72458-AT	-0.629174209	1.76E-55	negative
LSM4	DENND1B-9304-AT	0.647217889	1.28E-59	postive
LSM4	DENND1B-9305-AT	-0.647217541	1.28E-59	negative
LSM4	RARA-40856-AP	0.651170277	1.45E-60	postive
SDE2	PTCD2-72456-AT	-0.608898687	3.82E-51	negative
SDE2	PTCD2-72458-AT	0.60889427	3.83E-51	postive
SDE2	NUPL2-78961-AT	-0.619556076	2.20E-53	negative
SDE2	NUPL2-78960-AT	0.619563836	2.19E-53	postive
SDE2	HAS3-37253-AT	0.642483931	1.65E-58	postive
SDE2	HAS3-37254-AT	-0.642485107	1.65E-58	negative
DDX21	PTCD2-72456-AT	-0.619313009	2.48E-53	negative
DDX21	PTCD2-72458-AT	0.619311608	2.48E-53	postive
PQBP1	CABIN1-61386-AP	0.627986919	3.22E-55	postive
PQBP1	HAS3-37253-AT	-0.604573644	2.93E-50	negative
PQBP1	HAS3-37254-AT	0.604577051	2.93E-50	postive
PQBP1	RARA-40856-AP	0.7195159	1.86E-79	postive

PQBP1	EMP1-20539-ES	-0.637116313	2.87E-57	negative
CLK2	DYNLT1-78273-AP	0.643161503	1.15E-58	postive
CLK2	DYNLT1-78274-AP	-0.643161503	1.15E-58	negative
CLK2	NDUFB8-12824-AT	-0.618757555	3.26E-53	negative
CLK2	EXOSC10-645-RI	0.676160654	7.03E-67	postive
DHX34	DYNLT1-78273-AP	0.602437272	7.93E-50	postive
DHX34	DYNLT1-78274-AP	-0.602437272	7.93E-50	negative
RBBP6	USP4-64851-AT	0.638513318	1.37E-57	postive
RBBP6	MPV17-52966-AT	-0.641238498	3.22E-58	negative
DHX36	PTCD2-72456-AT	-0.626161445	8.12E-55	negative
DHX36	PTCD2-72458-AT	0.626157325	8.14E-55	postive
DHX36	NUPL2-78961-AT	-0.639220212	9.43E-58	negative
DHX36	NUPL2-78960-AT	0.639219873	9.44E-58	postive
DHX36	HAS3-37253-AT	0.614034202	3.26E-52	postive
DHX36	HAS3-37254-AT	-0.614036279	3.26E-52	negative
SNRPG	RARA-40856-AP	0.658965362	1.81E-62	postive
ISY1	CABIN1-61386-AP	0.63808323	1.72E-57	postive
ISY1	RARA-40857-AP	-0.646055606	2.40E-59	negative
ISY1	CREBZF-18139-RI	-0.608747952	4.10E-51	negative
ISY1	HAS3-37253-AT	-0.614606421	2.47E-52	negative
ISY1	HAS3-37254-AT	0.614607794	2.47E-52	postive
ISY1	RARA-40856-AP	0.725754208	1.83E-81	postive
ISY1	TGFBR3-3739-AP	-0.616553304	9.59E-53	negative
CPSF6	MPV17-52966-AT	-0.624413154	1.96E-54	negative
PRPF38B	NDUFB8-12824-AT	-0.610482668	1.80E-51	negative
DHX9	KIAA2013-694-AT	-0.611794229	9.59E-52	negative
SRRM1	USP4-64851-AT	0.605511269	1.89E-50	postive
SRRM1	C2orf68-54308-AT	-0.656374047	7.87E-62	negative
SRRM1	C2orf68-54309-AT	0.656366651	7.90E-62	postive
SRRM1	MPV17-52966-AT	-0.6995745	2.17E-73	negative
SRRM1	NDUFB8-12824-AT	-0.633725695	1.69E-56	negative
RBM42	RARA-40856-AP	0.612788001	5.95E-52	postive
LSM7	CABIN1-61386-AP	0.670246149	2.49E-65	postive
LSM7	CREBZF-18139-RI	-0.601378154	1.30E-49	negative
LSM7	HAS3-37253-AT	-0.635805642	5.70E-57	negative
LSM7	HAS3-37254-AT	0.635806707	5.70E-57	postive
LSM7	RARA-40856-AP	0.742258632	4.70E-87	postive
TCERG1	C5orf45-74948-RI	0.691107643	5.78E-71	postive
TCERG1	PTRH2-42790-AP	-0.622613053	4.82E-54	negative
TCERG1	PTRH2-42789-AP	0.622614786	4.82E-54	postive
TCERG1	DYNLT1-78273-AP	0.638547918	1.35E-57	postive
TCERG1	DYNLT1-78274-AP	-0.638547918	1.35E-57	negative
TCERG1	DAPK2-31076-ES	0.703954219	1.12E-74	postive
TCERG1	BCAT2-50816-ES	0.621283188	9.35E-54	postive
TCERG1	NDUFB8-12824-AT	-0.675219189	1.25E-66	negative
TCERG1	EXOSC10-645-RI	0.713831021	1.13E-77	postive
HNRNPH1	USP4-64850-AT	-0.62515865	1.35E-54	negative
HNRNPH1	IDH3G-90494-RI	0.609454156	2.93E-51	postive
HNRNPH1	TTLL5-28518-AT	-0.644115495	6.88E-59	negative
HNRNPH1	USP4-64851-AT	0.664587387	7.03E-64	postive
HNRNPH1	C5orf45-74948-RI	0.665417428	4.33E-64	postive
HNRNPH1	DYNLT1-78273-AP	0.617077939	7.42E-53	postive
HNRNPH1	DYNLT1-78274-AP	-0.617077939	7.42E-53	negative
HNRNPH1	PET100-47118-AT	-0.621800142	7.23E-54	negative
HNRNPH1	MPV17-52966-AT	-0.727600458	4.54E-82	negative
HNRNPH1	NDUFB8-12824-AT	-0.733151346	6.43E-84	negative
SNRPA	HAS3-37253-AT	-0.619304006	2.49E-53	negative
SNRPA	HAS3-37254-AT	0.619308051	2.48E-53	postive

SNRPA	RARA-40856-AP	0.638524346	1.36E-57	postive
SNRPA	ANKRD10-26274-ES	0.604366353	3.23E-50	postive
ARGLU1	BOP1-85554-RI	0.609904007	2.37E-51	postive
ARGLU1	ZNF331-51722-AP	0.609821408	2.46E-51	postive
ARGLU1	DYNLT1-78273-AP	0.638681857	1.25E-57	postive
ARGLU1	DYNLT1-78274-AP	-0.638681857	1.25E-57	negative
ARGLU1	DAPK2-31076-ES	0.626031849	8.67E-55	postive
ARGLU1	BCAT2-50816-ES	0.601769577	1.08E-49	postive
ARGLU1	EPOR-47693-ES	0.604319368	3.30E-50	postive
ARGLU1	NDUFB8-12824-AT	-0.614628078	2.45E-52	negative
ARGLU1	EXOSC10-645-RI	0.707245239	1.16E-75	postive
SART1	CABIN1-61386-AP	0.613436674	4.35E-52	postive
SART1	HAS3-37253-AT	-0.601433653	1.26E-49	negative
SART1	HAS3-37254-AT	0.601437262	1.26E-49	postive
TRA2A	EXOSC10-645-RI	0.625639503	1.06E-54	postive
YBX3	SHROOM4-89139-AP	-0.651228068	1.40E-60	negative
YBX3	SHROOM4-89138-AP	0.662359362	2.57E-63	postive
YBX3	CACNB3-21469-AP	-0.629110277	1.82E-55	negative
YBX3	TMEM79-8218-AP	-0.602238489	8.70E-50	negative
YBX3	TMEM79-8217-AP	0.608857544	3.90E-51	postive
SNRNP200	PTCD2-72456-AT	-0.625927478	9.14E-55	negative
SNRNP200	PTCD2-72458-AT	0.625923815	9.16E-55	postive
SNRNP200	WDR20-29338-AT	0.602616	7.30E-50	postive
SNRNP200	KIAA2013-694-AT	-0.608313799	5.04E-51	negative
THOC1	ZNF331-51722-AP	0.643164068	1.15E-58	postive
THOC1	DYNLT1-78273-AP	0.640582845	4.57E-58	postive
THOC1	DYNLT1-78274-AP	-0.640582845	4.57E-58	negative
THOC1	DAPK2-31076-ES	0.637066095	2.94E-57	postive
THOC1	BCAT2-50816-ES	0.638054565	1.75E-57	postive
THOC1	EXOSC10-645-RI	0.733197781	6.21E-84	postive
RBM5	BOP1-85554-RI	0.604615308	2.87E-50	postive
RBM5	CCDC53-24023-ES	0.660686787	6.73E-63	postive
RBM5	C5orf45-74948-RI	0.603578915	4.66E-50	postive
RBM5	DYNLT1-78273-AP	0.724304889	5.41E-81	postive
RBM5	DYNLT1-78274-AP	-0.724304889	5.41E-81	negative
RBM5	DAPK2-31076-ES	0.64640614	1.99E-59	postive
RBM5	BCAT2-50816-ES	0.651220627	1.41E-60	postive
RBM5	NDUFB8-12824-AT	-0.688920129	2.37E-70	negative
RBM5	EXOSC10-645-RI	0.737819106	1.65E-85	postive
PRPF3	DYNLT1-78273-AP	0.631023838	6.81E-56	postive
PRPF3	DYNLT1-78274-AP	-0.631023838	6.81E-56	negative
PRPF3	NDUFB8-12824-AT	-0.634619723	1.06E-56	negative
PRPF3	EXOSC10-645-RI	0.636130908	4.81E-57	postive
SAP30BP	RARA-40856-AP	0.605252127	2.13E-50	postive
SPEN	PTCD2-72456-AT	-0.638006784	1.79E-57	negative
SPEN	PTCD2-72458-AT	0.638004095	1.79E-57	postive
SPEN	NUPL2-78961-AT	-0.607215097	8.48E-51	negative
SPEN	NUPL2-78960-AT	0.607213295	8.48E-51	postive
CELF2	PTS-18764-ES	-0.610401381	1.87E-51	negative
SRSF6	EXOSC10-645-RI	0.601370931	1.30E-49	postive
DHX15	PTCD2-72456-AT	-0.646299007	2.11E-59	negative
DHX15	PTCD2-72458-AT	0.646294993	2.11E-59	postive
DHX15	KIAA2013-694-AT	-0.629464931	1.52E-55	negative
TTC14	BOP1-85554-RI	0.626906584	5.57E-55	postive
TTC14	TPM1-30985-AT	-0.64425037	6.39E-59	negative
TTC14	CCDC53-24023-ES	0.60452701	3.00E-50	postive
TTC14	HSP90AA1-29333-AT	-0.628405169	2.60E-55	negative
TTC14	DYNLT1-78273-AP	0.670310753	2.40E-65	postive

TTC14	DYNLT1-78274-AP	-0.670310753	2.40E-65	negative
TTC14	DAPK2-31076-ES	0.64631633	2.09E-59	postive
TTC14	NDUFB8-12824-AT	-0.651768959	1.04E-60	negative
TTC14	EXOSC10-645-RI	0.713098339	1.90E-77	postive
CDC40	CABIN1-61386-AP	-0.609224938	3.27E-51	negative
CDC40	PTCD2-72456-AT	-0.627381577	4.38E-55	negative
CDC40	PTCD2-72458-AT	0.627379024	4.39E-55	postive
CDC40	NUPL2-78961-AT	-0.633170988	2.25E-56	negative
CDC40	NUPL2-78960-AT	0.633171393	2.25E-56	postive
CDC40	KIAA2013-694-AT	-0.630293267	9.91E-56	negative
CDC40	RARA-40856-AP	-0.600866903	1.64E-49	negative
CDC40	ANKS1B-23885-AT	0.634595765	1.07E-56	postive
CDC40	ANKS1B-23886-AT	-0.634591	1.07E-56	negative
CLASRP	ZNF331-51722-AP	0.652300039	7.74E-61	postive
CLASRP	DYNLT1-78273-AP	0.657422688	4.34E-62	postive
CLASRP	DYNLT1-78274-AP	-0.657422688	4.34E-62	negative
CLASRP	BCAT2-50816-ES	0.627191767	4.82E-55	postive
CLASRP	EPOR-47693-ES	0.606738912	1.06E-50	postive
CLASRP	EXOSC10-645-RI	0.658999877	1.77E-62	postive
RALY	CABIN1-61386-AP	0.6074004	7.77E-51	postive
RALY	RARA-40856-AP	0.62747801	4.17E-55	postive
RNPC3	BOP1-85554-RI	0.618284115	4.11E-53	postive
RNPC3	PTK2B-83155-AT	-0.60832364	5.02E-51	negative
RNPC3	HSP90AA1-29333-AT	-0.621846576	7.07E-54	negative
RNPC3	DAPK2-31076-ES	0.619032303	2.84E-53	postive
RNPC3	MPV17-52966-AT	-0.639685227	7.37E-58	negative
RNPC3	NDUFB8-12824-AT	-0.660125856	9.29E-63	negative
RNPC3	EXOSC10-645-RI	0.643861025	7.89E-59	postive
INTS3	FNTA-83754-AD	0.603183221	5.61E-50	postive
INTS3	USP4-64850-AT	-0.698577458	4.23E-73	negative
INTS3	INO80C-45173-AT	-0.636052259	5.01E-57	negative
INTS3	INO80C-45172-AT	0.636055186	5.00E-57	postive
INTS3	TTLL5-28518-AT	-0.667857167	1.03E-64	negative
INTS3	USP4-64851-AT	0.719125572	2.47E-79	postive
INTS3	PTK2B-83155-AT	-0.671889019	9.33E-66	negative
INTS3	ITFG2-19715-AT	-0.623858199	2.59E-54	negative
INTS3	C5orf45-74948-RI	0.62890859	2.01E-55	postive
INTS3	DAPK2-31076-ES	0.625394566	1.20E-54	postive
INTS3	MPV17-52966-AT	-0.672652081	5.90E-66	negative
INTS3	NDUFB8-12824-AT	-0.643182094	1.14E-58	negative
SRSF5	DYNLT1-78273-AP	0.615645912	1.49E-52	postive
SRSF5	DYNLT1-78274-AP	-0.615645912	1.49E-52	negative
SRSF5	EPOR-47693-ES	0.629381692	1.58E-55	postive
SRSF5	NDUFB8-12824-AT	-0.622117692	6.17E-54	negative
SRSF5	EXOSC10-645-RI	0.645443983	3.35E-59	postive
SNRNP35	RARA-40856-AP	0.713845557	1.12E-77	postive
SNRNP35	EMP1-20539-ES	-0.614154116	3.08E-52	negative
GPATCH8	PTCD2-72456-AT	-0.665896793	3.27E-64	negative
GPATCH8	PTCD2-72458-AT	0.6658927	3.28E-64	postive
GPATCH8	NUPL2-78961-AT	-0.63760763	2.21E-57	negative
GPATCH8	NUPL2-78960-AT	0.637605496	2.21E-57	postive
GPATCH8	C2orf68-54308-AT	-0.694888584	4.89E-72	negative
GPATCH8	C2orf68-54309-AT	0.694894841	4.87E-72	postive
GPATCH8	KIAA2013-694-AT	-0.682632473	1.28E-68	negative
GPATCH8	KCNC3-51173-AT	-0.64382351	8.05E-59	negative
GPATCH8	KCNC3-51172-AT	0.643829991	8.02E-59	postive
GPATCH8	MYEF2-30481-AT	-0.615074061	1.97E-52	negative
GPATCH8	MPV17-52966-AT	-0.664536001	7.25E-64	negative

GPATCH8	CNOT2-23366-AT	0.639270832	9.18E-58	postive
ZC3H13	PTCD2-72456-AT	-0.645434962	3.37E-59	negative
ZC3H13	PTCD2-72458-AT	0.645430292	3.38E-59	postive
ZC3H13	NUPL2-78961-AT	-0.606471436	1.20E-50	negative
ZC3H13	NUPL2-78960-AT	0.606469367	1.20E-50	postive
ZC3H13	KIAA2013-694-AT	-0.614513813	2.58E-52	negative
